# All-photonic kinase inhibitors: light-controlled release-and-report inhibition†

**DOI:** 10.1039/d4sc00390j

**Published:** 2024-04-12

**Authors:** Cassandra L. Fleming, Carlos Benitez-Martin, Elin Bernson, Yongjin Xu, Linnea Kristenson, Tord Inghardt, Thomas Lundbäck, Fredrik B. Thorén, Morten Grøtli, Joakim Andréasson

**Affiliations:** a Department of Chemistry and Chemical Engineering, Physical Chemistry, Chalmers University of Technology SE-41296 Göteborg Sweden a-son@chalmers.se; b Department of Chemistry and Molecular Biology, University of Gothenburg Box 462 SE-40530 Göteborg Sweden grotli@chem.gu.se; c TIMM Laboratory at Sahlgrenska Centre for Cancer Research, Department of Obstetrics and Gynecology, Institute of Clinical Sciences, Sahlgrenska Academy, University of Gothenburg SE-41296 Göteborg Sweden; d TIMM Laboratory, Sahlgrenska Centre for Cancer Research, Department of Infectious Diseases, Institute of Biomedicine, Sahlgrenska Academy, University of Gothenburg SE-41296 Göteborg Sweden; e Cardiovascular, Renal and Metabolism, Innovative Medicines and Early Development, AstraZeneca SE-43183 Mölndal Sweden; f Mechanistic and Structural Biology, Discovery Sciences, R&D, AstraZeneca SE-43183 Mölndal Sweden; g TIMM Laboratory, Sahlgrenska Centre for Cancer Research, Department of Medical Biochemistry and Cell Biology, Institute of Biomedicine, Sahlgrenska Academy, University of Gothenburg SE-41296 Göteborg Sweden

## Abstract

Light-responsive molecular tools targeting kinases affords one the opportunity to study the underlying cellular function of selected kinases. In efforts to externally control lymphocyte-specific protein tyrosine kinase (LCK) activity, the development of release-and-report LCK inhibitors is described, in which (i) the release of the active kinase inhibitor can be controlled externally with light; and (ii) fluorescence is employed to report both the release and binding of the active kinase inhibitor. This introduces an unprecedented all-photonic method for users to both control and monitor real-time inhibitory activity. A functional cellular assay demonstrated light-mediated LCK inhibition in natural killer cells. The use of coumarin-derived caging groups resulted in rapid cellular uptake and non-specific intracellular localisation, while a BODIPY-derived caging group predominately localised in the cellular membrane. This concept of release-and-report inhibitors has the potential to be extended to other biorelevant targets where both spatiotemporal control in a cellular setting and a reporting mechanism would be beneficial.

## Abstract 2

An all-photonic method is described, in which (i) the release of an active kinase inhibitor is controlled externally with light; and (ii) fluorescence is employed to report both the release and binding of the inhibitor to its corresponding target.

## Graphical abstract

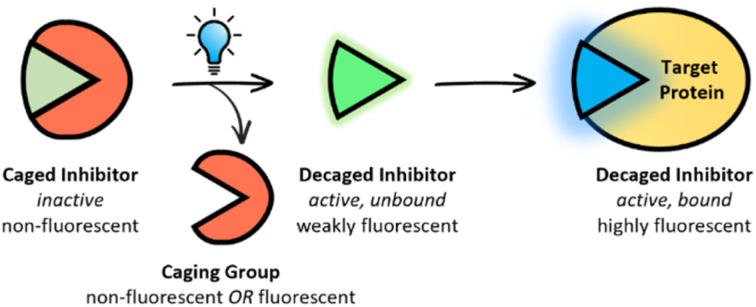

## Introduction

Light-responsive chemical probes have the potential to unveil the fundamental molecular events of complex cellular processes. In this context, light serves as a valuable non-invasive external stimulus and enables control of when and where drug activation occurs. This, in turn, addresses some of the underlying challenges associated with the poor selectivity of conventional drugs.^1–3^

The external control of kinase inhibition has gained increasing attention of late, in which both reversible and irreversible photoactivation of kinase inhibition have been achieved.^4–12^ Photocaging has been a popular approach whereby a photolabile protecting moiety is strategically introduced onto a kinase inhibitor to prevent key interactions with their target enzyme. Inhibitory activity is only restored upon exposure to light and concomitant liberation of the active compound. The majority of photocaged kinase inhibitors reported to date are reliant on UV light to achieve photoactivation, which limits their use in cellular contexts.^4–9^ While the use of fluorescence caging groups that exhibit OFF–ON diagnostic changes in emission upon decaging have been employed to monitor the photorelease of caged substrates in a cellular environment,^13–17^ the use of a ‘reporting mechanism’ for both photoactivation and binding of the decaged active inhibitor to the target kinase has yet to be fully explored.

The lymphocyte-specific protein tyrosine kinase (LCK) belongs to the Src family of non-receptor tyrosine kinases.^18,19^ LCK plays an essential role in natural killer (NK)-cell and T-cell signalling, in which LCK inhibition has shown potential for the therapeutic treatment of leukaemia and autoimmune diseases, such as rheumatoid arthritis.^20–23^ More recently, LCK has been identified as an important regulator of neuronal morphology and memory functions.^24^ However, the involvement of LCK activity in these critical processes within the central nervous system remains poorly understood. Given the cell and tissue-specific activity of LCK, molecular tools allowing to probe the role of LCK in both health and disease states with high spatiotemporal resolution are needed. The ability to manipulate the activity of LCK using light would result in spatiotemporal control of enzymatic activity, thus serving as a valuable tool to study LCK function. Furthermore, the visualisation of LCK inhibitors in live cells allows the user to monitor their therapeutic efficiency in real-time, providing further insight into *e.g.*, their action mechanisms and the favoured subcellular localisation.

**Fig. 1:**
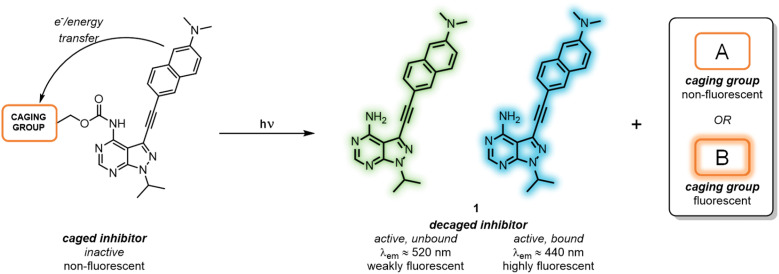
Working principle of proposed caged inhibitors. Biologically active LCK inhibitor 1 is released by means of light, and only shows strong emission upon binding to LCK enzyme. Different caging groups are incorporated, classifying them as (A) non-fluorescent or (B) fluorescent.

As a means to externally control LCK activity, as well as monitoring in real-time the photoactivation and the subsequent binding to the enzyme, we report the development of a series of caged LCK inhibitors where light is employed to: (i) trigger the release of the LCK inhibitor with high spatiotemporal resolution; (ii) monitor the decaging process, and; (iii) report the binding of the ‘active’ inhibitor to the LCK enzyme (Fig. 1). This has been achieved through the introduction of a photolabile caging moiety onto the previously reported solvatochromic LCK inhibitor 1, in which diagnostic changes in emission are observed upon binding the kinase enzyme (Fig. 1).^25^ In doing so, the caged form serves as a poor inhibitor of LCK while the intrinsic fluorescence of the LCK inhibitor is quenched by electron and/or energy transfer mechanisms. Upon exposure to a certain wavelength of light, the caging group is cleaved to liberate the unbound form of the inhibitor which exhibits weak fluorescence in aqueous solution. Only upon binding to the ATP-binding site of the LCK enzyme does the solvatochromic inhibitor exhibit a substantial increase in emission intensity and a blue-shift of the emission maxima — reporting back to the user that the inhibitor is bound to its target enzyme. More specifically, two different classes of caging groups have been installed that are either (A) non-fluorescent, or (B) fluorescent (Fig. 1). While the former are ‘spectroscopically silent’ and simply mask the activity of 1 at both biological and photophysical levels, the incorporation of fluorescent caging groups is envisioned to further assist the reporting of the release process. It was anticipated that the fluorescence attributed to the caging group would be strongly quenched in the caged inhibitors. However, when exposed to light, the release of a highly emissive caging unit would allow for the convenient monitoring of the decaging process *via* a fluorescence readout.

## Results and discussion

### Design and synthesis of release-and-report kinase inhibitors

We have previously reported the development of the fluorescent LCK inhibitor 1, in which diagnostic changes in emission was observed upon binding to LCK.^25^ The pyrazolo[3,4-*d*]pyrimidine heterocycle commonly features in a number of type I kinase inhibitors, whereby the exocyclic amino moiety partakes in key binding interactions in the ATP-binding site.^26–28^ Therefore, the inclusion of a caging group at this position was pursued as it should prevent the formation of these key hydrogen bond interactions, rendering inhibitor 1 ‘inactive’.

Among the caging groups described in the literature,^29,30^ those fulfilling the following criteria were selected: (i) applicability in biological contexts; (ii) can be cleaved at wavelengths that will not induce cellular damage (*λ* > 320 nm); and (iii) the corresponding decaged product is non-toxic. As BODIPY- and coumarin-derived caging groups fulfil these requirements,^29–34^ the inclusion of such caging groups onto LCK inhibitor 1*via* a carbamate linker was investigated. Due to the hydrophobic nature of coumarin-derived caging groups, the dimethylester-coumarin variant was also included in this study (compound 3c), as it was envisaged that the methylesters would serve as valuable synthetic handles for further functionalisation to tailor *e.g.*, water solubility if required. Furthermore, the popular *o*-nitrobenzyl caging group was also included as despite their reliance on UV-light and the formation of a reactive decaged product, they have been widely utilised in a number of biological applications.^32,34^ The presence of a methyl group at the benzylic position of the *o*-nitrobenzyl caging group was also considered (compound 4b), as this would afford a ketone as the decaged product rather than the more reactive aldehyde. Thereby, the effect of the benzylic methyl group on the performance of the decaging reaction was of interest.

**Scheme 1:**
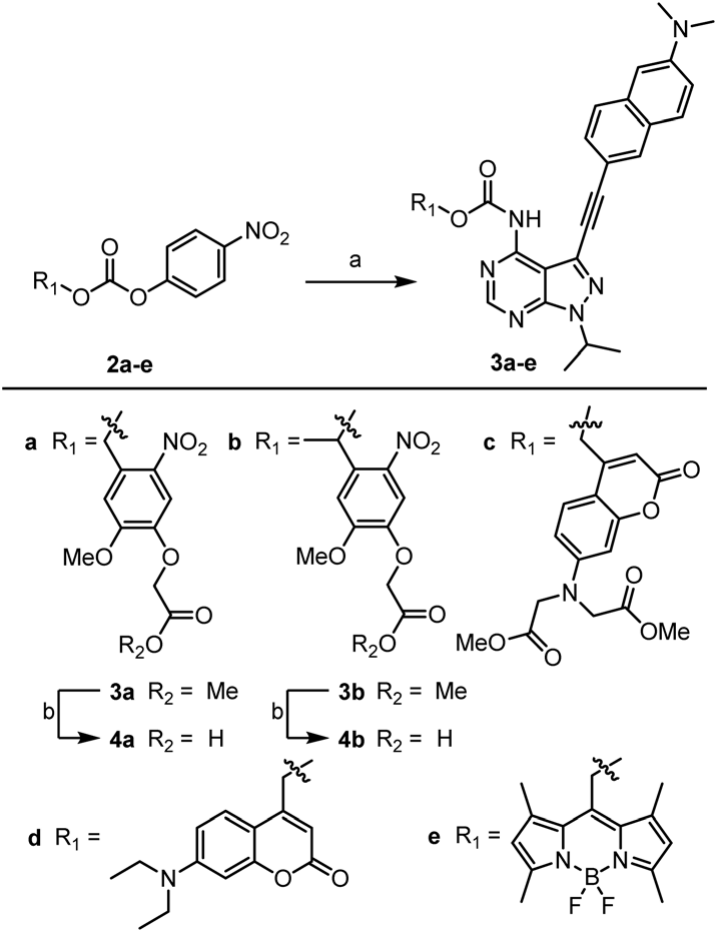
Synthesis of caged LCK inhibitors. Reagents and conditions: (a) compound 1, HOBt, CH_2_Cl_2_, 40 °C, 4–7 days; (b) LiOH·H_2_O, (1 : 1) THF/H_2_O, 21 °C, 45 min.

As shown in Scheme 1, the caged derivatives were synthesised by stirring activated carbonate building blocks 2a–e, LCK inhibitor 1 and HOBt in CH_2_Cl_2_ at 40 °C (see ESI† for full synthetic details). Hydrolysis of the methyl ester in carbamates 3a and 3b with LiOH·H_2_O in THF/H_2_O (1 : 1) afforded caged derivatives 4a and 4b, respectively.

### Photophysical evaluation of photocaged LCK inhibitors

The spectroscopic/photophysical data of 4a, 4b and 3c–e are summarised in Table 1. The emission properties of compounds 4a and 4b are comparable to those of 1 in terms of both spectral position and fluorescence quantum yield. This shows that the caging groups used in these analogues are not significantly affecting the spectroscopic properties of the caged conjugate. In contrast, caged inhibitors 3c–e equipped with intrinsically fluorescent coumarin and BODIPY caging groups display weak fluorescence, which originates from these building blocks rather than from the inhibitor itself. This is evident from the fluorescent quantum yields, as these are slightly higher than that observed from 1 (Table 1). However, when compared to the corresponding coumarin and BODIPY model monomers (compounds S12, S16 and S20; Table S1†), the fluorescence in compounds 3c–e is indeed efficiently quenched.

**Table 1:** Spectroscopic data for compound 1 and caged derivatives 4a, 4b and 3c–e in aqueous solution (10% DMSO in H_2_O)^a^

Compound	*λ*_abs_ b (nm)	*ε*_max_ c (M^−1^ cm^−1^)	*λ*_em_ d (nm)	*ϕ*_F_ e	*ϕ*_dc_ f	*ε*·*ϕ*_dc_^g^ (M^−1^ cm^−1^)
1	347	27 300	517	0.001	N/A	N/A
4a	307^h^, 338	25 100	nd	nd	0.001^i^	20^i^
4b	339, 365^h^	27 100	nd	nd	0.008^i^	2 × 10^2^^i^
3c	366	39 300	455	0.07	0.06^i^	2 × 10^3^^i^
3d	377	41 100	492	0.006	0.03^k^	8 × 10^2^^j^
3e	384, 531	65 900	439, 520	0.003	0.006^k^	4 × 10^2^^k^

a Samples were prepared at a concentration of *ca.* 10 μM. N/A = not applicable. nd = not determined.

b Wavelength of absorption maximum.

c Due to poor solubility in aqueous solution at high concentrations, the molar absorption coefficient was determined in DMSO.

d Wavelength of emission maximum.

e Fluorescence quantum yields were determined by taking 1,9-diphenylanthracene (DPA) in cyclohexane (*ϕ*_F_ = 0.97) as a reference. Excitation wavelength at 350 nm.

f Decaging quantum yields determined from UV/vis absorption measurements.

g Decaging cross section (the product of the molar absorption coefficient at the irradiation wavelength and the decaging quantum yield).

h Shoulder.

i Decaging wavelength at 365 nm.

j Decaging wavelength at 405 nm.

k Decaging wavelength at 523 nm.

To investigate whether photoinduced electron transfer (PET) can contribute to the fluorescence quenching in the caged compounds 3c–e, electrochemical data of the model monomers were determined in acetonitrile or DMSO. The cyclic voltammograms are shown in Fig. S21.† All compounds were characterised by non-reversible or quasi-reversible electrochemical processes. The analysis according to the Rehm–Weller equation revealed that PET is thermodynamically favourable in the BODIPY- and coumarin-derived monomers (compounds S12, S16 and S20 in ESI†), thus fulfilling the design criteria detailed in Fig. 1.

The decaging reactions were evaluated in aqueous solution, in which DMSO was required to aid solubility (10% DMSO in H_2_O). Samples were irradiated with LEDs at 365 nm, 405 nm, or 523 nm depending on the absorption spectrum of the respective cage and the reactions were monitored by recording absorption spectra at regular intervals.

Upon analysis of the decaging reactions by UV/vis spectroscopy, it is evident that the overall kinetics are not always described by mono-exponential processes (Fig. S1 and S2†). This observation is ascribed to the photo- or thermal-decomposition of the released products, including the decaged inhibitor 1 and the reactive species derived from the *o*-nitrobenzyl group. The notion of photodecomposition of the decaged inhibitor 1 is supported by the significant spectral changes observed when LCK inhibitor 1 alone is irradiated with 365 nm (*ca*. 30 mW cm^−2^) for 10 minutes (Fig. S5†). This would imply that the absorption spectra recorded during photoirradiation of compounds 4a and 4b capture both the decaging and subsequent photodecomposition processes. It is therefore important to note that the reported decaging quantum yields may not be reflective of a clean photolysis reaction and that these values will also be influenced by any photodecomposition that may have occurred during these measurements. The inclusion of the methyl moiety on the benzylic position of the *o*-nitrobenzyl caging group substantially increases the decaging cross section (*ε*·*ϕ*_dc_, the product of molar absorption coefficient at the irradiation wavelength and decaging quantum yield). This is clearly seen when comparing the values for 4b (2 × 10^2^ M^−1^ cm^−1^) and 4a (20 M^−1^ cm^−1^, see Table 1). It is also interesting to note that the thermal stability increased dramatically with the inclusion of the methyl group (4b, Fig. S10†). The decaging cross section further increased for the coumarin-derived caged inhibitor 3c (2 × 10^3^ M^−1^ cm^−1^) when compared to the *o*-nitrobenzyl derivatives. Due to the efficient nature of this decaging reaction, minimal photodegradation of active LCK inhibitor 1 was observed (Fig. S3†).

**Fig. 2:**
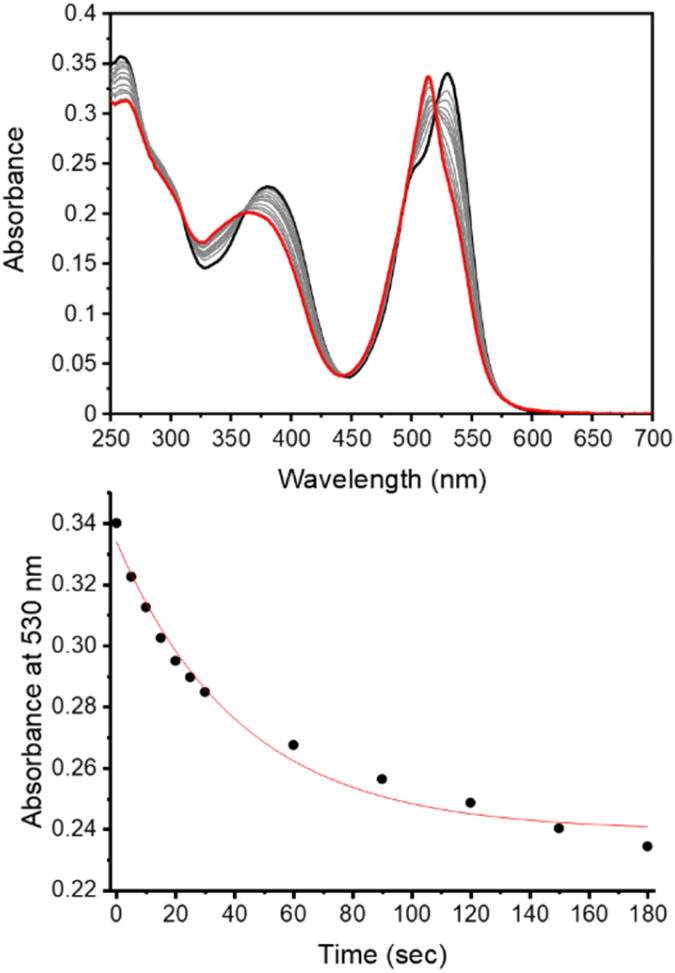
Monitoring the decaging reaction of 3e using UV/vis absorption spectroscopy. (top) Absorption spectra of 3e in aqueous solution before irradiation (black) and after irradiation at 523 nm for 180 seconds (red, power ≈ 8 mW cm^−2^); (bottom) changes in absorbance at 530 nm upon irradiation at 523 nm (0–180 seconds).

The observed photodegradation of the active LCK inhibitor 1 upon continuous irradiation with 365 nm stresses the need to utilise caging groups that employ longer wavelengths of light for the photolytic decaging reaction. Given the harmful effects of UV light towards biological samples and its poor tissue penetration, the use of longer wavelengths of light would also impart enhanced biocompatibility. The absorption spectrum of the coumarin caged inhibitor 3d shows strong absorption at 400 nm and a rapid decrease in the absorbance maximum of 3d was observed upon irradiation with 405 nm (*ca*. 27 mW cm^−2^, Fig. S4†). The absorption of the BODIPY caged inhibitor 3e shows two well-defined bands at 384 nm and 531 nm, corresponding to the inhibitor and BODIPY caging group, respectively (Fig. 2). Upon irradiation at 523 nm (*ca*. 8 mW cm^−2^) a rapid decrease at 530 nm was observed. Importantly, photodecomposition of 1 was not observed upon continuous irradiation with 523 nm (Fig. S7†).

Given the fluorescent nature of the coumarin and BODIPY decaged products (*ϕ*_F_ > 0.07, Table S1†), the decaging reaction of these caged substrates (3c–e) could also be monitored from the increase in emission arising from the generation of the fluorescent decaged product (Fig. S22–S24†). Due to the weak fluorescent properties of 1 in aqueous solution, minimal changes in the emission occurring from the LCK inhibitor at 517 nm was observed upon decaging. From the UV/vis experiments, decaging cross sections of 8 × 10^2^ M^−1^ cm^−1^ and 4 × 10^2^ M^−1^ cm^−1^ were determined for 3d and 3e, respectively (Table 1).

### IC_50_ determination of LCK inhibitor 1 and caged inhibitors 3c–e

As no significant photodecomposition was observed of the decaged LCK inhibitor 1 during the photoinduced decaging reaction for 3c, 3d and 3e, the inhibitory activity for LCK of these caged substrates and the uncaged inhibitor 1 was assessed using the ADP-Glo™ Kinase Assay from Promega. Under these assay conditions, an IC_50_ of 2.4 μM was obtained for compound 1 (Fig. S30†), while no inhibitory activity against LCK was observed for all caged substrates (Fig. S31–S33†). This clearly shows that attachment of the selected caging groups efficiently blocks the inhibitory action of inhibitor 1.

### Fluorescence titration studies of caged inhibitor 3e with LCK

**Fig. 3:**
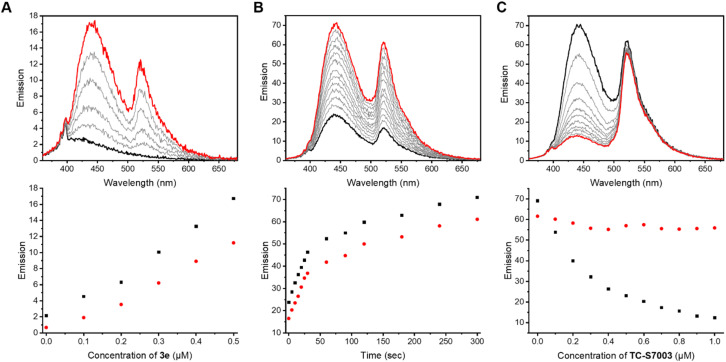
Fluorescence titration studies of LCK with BODIPY-derived caged inhibitor 3e. (A) Fluorescence titration of LCK (1 μM) with caged inhibitor 3e in Tris buffer, pH 7.5, (top) emission spectra before (black) and after the addition of 0.5 μM (red) of 3e, employing 350 nm as the excitation wavelength; (bottom) monitoring the changes in fluorescence intensity at 442 nm (black) and 520 nm (red) upon increasing concentration of 3e (0–0.5 μM). (B) (top) emission spectra of 3e (0.5 μM) with LCK (1 μM) in tris buffer, pH 7.5, before irradiation (black) and after irradiation at 523 nm (red), employing 350 nm as the excitation wavelength for emission readout; (bottom) monitoring the changes in fluorescence intensity at 442 nm (black) and 520 nm (red) upon irradiation of caged inhibitor 3e with 523 nm. (C) Displacement fluorescence titration, displacing decaged 1 from the ATP-binding site of LCK with the ATP-competitive inhibitor TC-S7003, (top) emission spectra before (black) and after the addition of 1.0 μM (red) of TC-S7003, employing 350 nm as the excitation wavelength; (bottom) changes in the fluorescence intensity at 442 nm (black) and 520 nm (red) upon increasing of ATP-competitive LCK inhibitor TC-S7003 (0–1.0 μM).

Previous work demonstrated the solvatochromic properties of LCK inhibitor 1*via* fluorescence titration experiments, in which a dramatic increase in emission intensity and a blue-shift in the emission maximum were clearly observed in concert with binding to LCK.^25^ In order to determine how the inclusion of the caging group on the exocyclic amino moiety of 1 affects these diagnostic changes in fluorescence upon binding, additional fluorescent titration experiments were performed. As the emission from the BODIPY caging group does not overlap with the emission arising from 1, which allows for discrimination of the fluorescence from BODIPY and 1, the caged inhibitor 3e was chosen for this study. The fluorescence titration commenced with the addition of aliquots of the BODIPY-caged inhibitor 3e to a solution of the LCK enzyme (1 μM), until a total of 0.5 μM of 3e was added (Fig. 3A). Over the course of the addition, changes in emission maxima and intensity were monitored at both 442 nm (fluorescence from inhibitor) and 520 nm (fluorescence from BODIPY). The linear nature of the fluorescence intensities *versus* concentration signals that there is no strong specific binding of the caged inhibitor 3e to LCK, and that the BODIPY caging group is preventing the LCK inhibitor from binding to the hydrophobic active site. After the titration, the sample was exposed to 523 nm light. This resulted in a dramatic increase in emission intensity at 442 nm and 520 nm, indicating the release and binding of the ‘active’ LCK inhibitor 1 as well as the release of the fluorescent decaged BODIPY product, respectively (Fig. 3B). It is proposed that specific binding of 1 to the ATP-binding site of the LCK enzyme is responsible for the significant increase in emission at 442 nm. Indeed, inhibitor 1 emits fluorescence centred at 517 nm in aqueous solution. However, binding to the hydrophobic pocket of LCK shifts the emission maxima to 442 nm, as demonstrated in a prior study.^25^

**Fig. 4:**
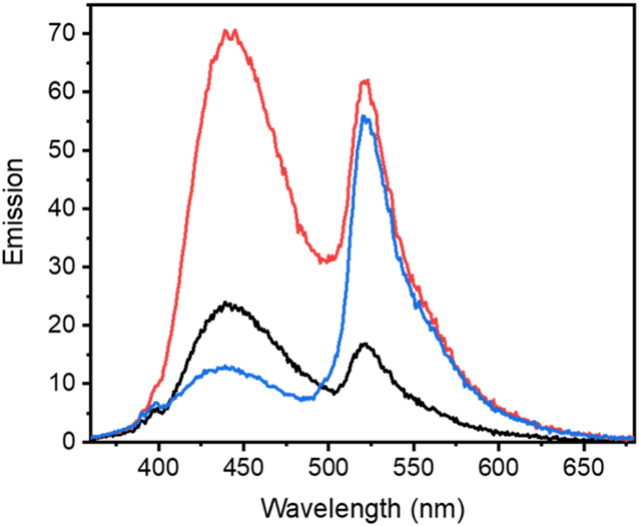
Comparison of emission spectra from fluorescence titration studies of LCK with BODIPY-derived caged inhibitor 3e before irradiation (black), after irradiation (red) with 523 nm where decaged 1 is bound to LCK and after decaged 1 has been displaced from the ATP-binding site (blue).

In efforts to support that the observed increase in fluorescence at 442 nm is due to inhibitor 1 binding to the hydrophobic pocket of LCK, a displacement experiment with the non-fluorescent ATP-competitive LCK inhibitor, TC-S7003 (IC_50_ = 7 nM)^35^ was performed. Upon the addition of TC-S7003 (0–1.0 μM) to the sample containing decaged LCK inhibitor 1, decaged BODIPY S20 and LCK enzyme, a substantial decrease in emission from LCK inhibitor 1 at 442 nm was observed, while minimal changes in emission from the decaged BODIPY product S20 (520 nm) occurred (Fig. 3C). These observations clearly show that fluorescence monitoring allows for tracking of the inhibitory action of this compound. The “extreme” spectra are collected in Fig. 4, where it is seen that ratiometric intensity readings can be used to follow both decaging and binding to the enzyme.

As the fluorescence quantum yield of 1 is much lower in aqueous solution compared to when bound to the LCK enzyme, the abovementioned observations strongly suggests that 1 binds specifically to the enzyme active site upon decaging, and is subsequently displaced by the addition of TC-S7003. Encouraged by these findings, we undertook further studies in live cells.

### Cellular uptake and intracellular decaging of compounds 3c–e in PBMCs

**Fig. 5:**
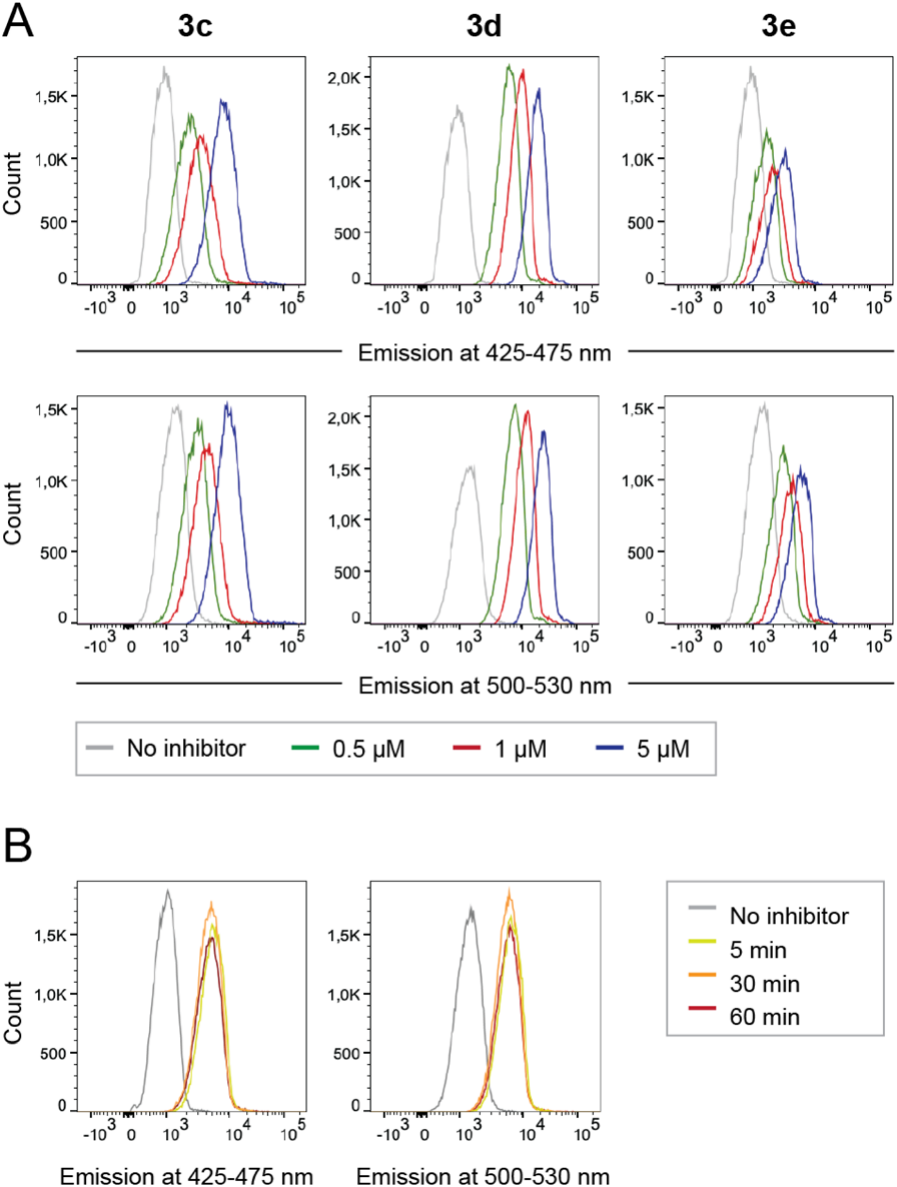
Cellular uptake of caged LCK inhibitors 3c–e. (A) Flow cytometry data show increased emission at wavelengths 425–475 nm and 500–530 nm, upon cellular uptake of caged LCK inhibitor as indicated. (B) Timing of caged LCK inhibitor cellular uptake. Representative flow cytometry data showing cellular uptake of caged 3c after incubation with 5 μL 3c for indicated times.

Peripheral blood mononuclear cells (PBMCs) were incubated with caged inhibitors 3c–e. Using a flow cytometry-based method (5-laser BD LSR Fortessa), cells were exposed to light at 355 nm and emission measured at 425–475 nm and 500–530 nm. As depicted in Fig. 5A, all caged inhibitors were taken up by the cells, as cells emitted light at higher intensity when incubated with the caged LCK inhibitors. The concentration dependence of the fluorescence intensity shown in Fig. 5A further supports this notion. The caged inhibitors were taken up after only 5 minutes of incubation (Fig. 5B). Moreover, cell morphology was kept unchanged, indicating no drastic impact on cell viability (Fig. S34†).

**Fig. 6:**
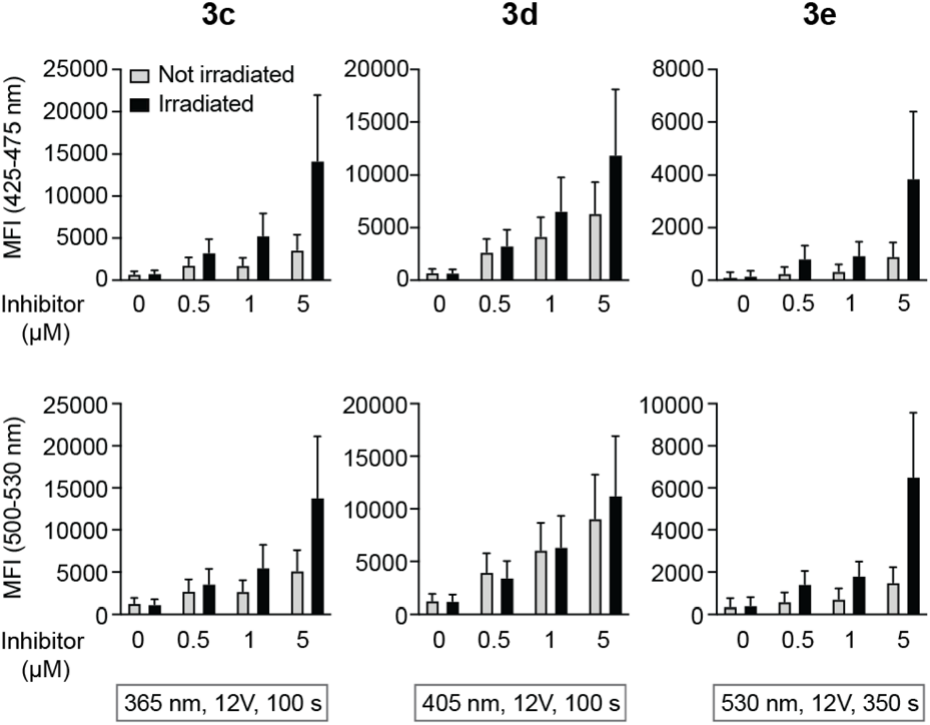
Decaging of caged LCK inhibitors after cellular uptake. Emission at 425–475 or 500–530 nm in lymphocytes that were first incubated with caged LCK inhibitor and subsequently exposed to light at indicated wavelength and time or kept in the dark. Error bars show SD of the fluorescence intensity.

Intracellular decaging was investigated by irradiating cells pre-incubated with the caged inhibitors at 365 nm (100 s), 405 nm (100 s), and 523 nm (350 s) for 3c, 3d, and 3e, respectively. Subsequently, cells were exposed to light at 355 nm for emission readout at 425–475 nm and 500–530 nm, respectively, using flow cytometry. Irradiation of the cells at the indicated wavelengths indeed resulted in the release of the active LCK inhibitor 1, as the emission intensities at 425–475 nm and 500–530 nm were increased as compared to cells not exposed to decaging light (Fig. 6).

### Light-mediated LCK inhibition in NK cell line

NK cells are innate immune cells with an intrinsic capacity to kill target cells upon activation. NK cells are activated *via* a set of activating receptors that upon binding to ligands on encountered cells induce a signalling cascade that leads to NK cell activation. The release of cytotoxic granules (degranulation) and/or cytokine production occurs as a result of NK cell activation, which ultimately leads to NK cell killing of the target cells. Among the activating receptors is NKG2D, for which the activating signalling cascade requires the presence of LCK kinase.^36^

To obtain a functional cellular assay where NK cell responses are dependent on LCK inhibition, we made use of a recently described CRISPR-Cas9-modified K562 cell line, in which ligands to key activating NK receptors other than LCK-dependent NKG2D were knocked-out.^37^ These ligands were B7-H6, which binds to NKp30, as well as PVR and Nectin-2, both of which binds to DNAM-1. These triple knock-out (tKO) K562 cells thus become more dependent on NKG2D signalling, and accordingly on the presence of active LCK kinase.

**Fig. 7:**
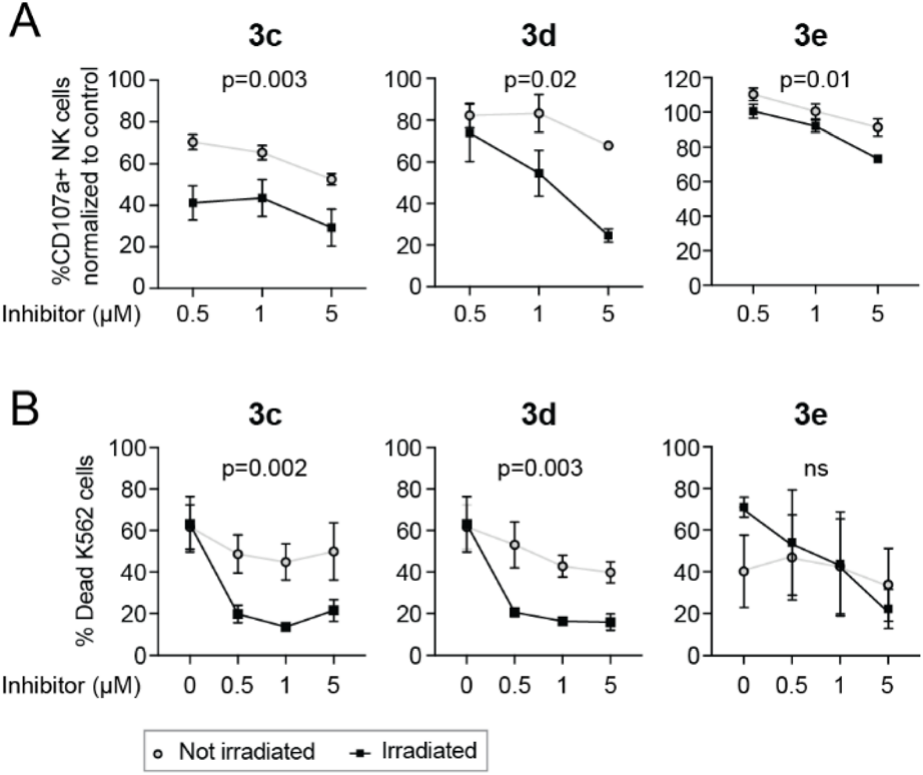
NK cell degranulation (A) and frequency of dead tKO K562 cells (B) after 3 h coincubation of NK cells and tKO K562 target cells, in the presence or absence of caged LCK inhibitor (0.5, 1 and 5 μM), with and without light irradiation for decaging. In (A), data were normalized to control with no inhibitor present. Two-way ANOVA, *p*-values represent impact of irradiation on degranulation (A) or cytotoxicity (B).

NK cells activated overnight in the presence of IL-2 (500 IU mL^−1^; PeproTech) were incubated with caged LCK inhibitors and either exposed to light using a LED-array (irradiation conditions identical to the flow cytometry experiments previously described, Fig. 6) or kept in the dark. tKO K562 cells, stained with the Cell Trace™ CFSE Cell proliferation kit (Invitrogen), were added to the NK cells, and the cells were subsequently co-incubated in the presence of a CD107a-PE antibody (BD Biosciences) for 3 hours at 37 °C, 5% CO_2_. The CD107a antibody allows for detection of activated degranulating NK cells.^38^ Cells were also stained with a LIVE/DEAD® Fixable Near IR Dead stain kit (Invitrogen) to be able to determine the viability of the tKO K562 cells after exposure to NK cells. All analyses were performed using a BD LSR Fortessa SORP flow cytometer. In the absence of tKO K562 cells, the caged inhibitors had no effect on NK cell degranulation. For compounds 3c and 3d, both NK cell degranulation (Fig. 7A) and NK cell lysis of tKO K562 cells (Fig. 7B) were significantly decreased upon irradiation compared to the non-irradiated caged compounds, clearly demonstrating light-induced activation of these compounds. Surprisingly, irradiation of 3e did not result in any significant change in NK cell degranulation or tKO K562 cell viability, suggesting that efficient decaging of compound 3e is not occurring within the NK cells.

### Fluorescence bioimaging of caged inhibitors 3c–e in NK cells

As a means to visualise cellular distribution, NK cells were incubated with 5 μM solutions of 3c–e and then either irradiated at the respective wavelengths for decaging or kept in the dark before analysis *via* fluorescence microscopy.

**Fig. 8:**
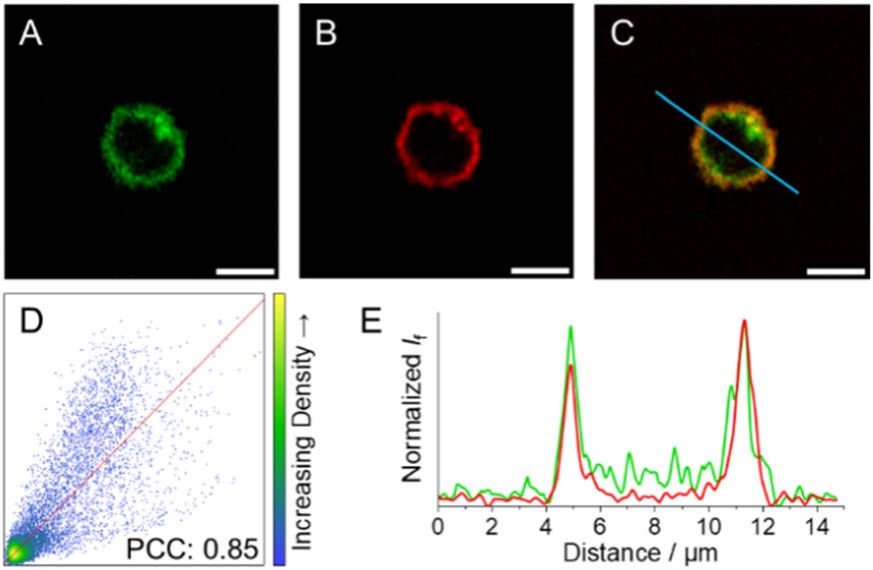
Fluorescence images of NK cells incubated with (A) 3e upon exposure to 523 nm light (green, *λ*_exc_ = 532 nm; refer to ESI† for details on light treatment before bioimaging), and (B) CellMask™ Deep Red (red, *λ*_exc_ = 633 nm). (C) Colocalization-merged images of 3e and CellMask™ Deep Red. (D) Correlation plot and PCC for 3e and CellMask™ Deep Red images. (E) Emission intensity profile plots along the blue line for the different compounds under study. Scale bar: 5 μm.

After incubation and subsequent irradiation with light to initiate the decaging reaction, blue-to-green fluorescence arising from the cytoplasm without any apparent subcellular distribution was observed for compounds 3c and 3d (Fig. S38 and S39†). In contrast, live 3e-treated NK cells exposed to 523 nm light display green fluorescence localised at the cell periphery, pointing to selective accumulation of 3e within the cell membrane (Fig. S40† and 8A). To further evaluate the subcellular localisation of 3e, NK cells were co-incubated with a cell membrane specific probe (CellMask™ Deep Red) as a counterstain to 3e (Fig. 8B). The large degree of colocalisation of 3e and CellMask™ Deep Red illustrated in Fig. 8C supports the proposed subcellular localisation for 3e and hence reduced internalisation within NK cells. The colocalisation is supported by (i) the Pearson correlation coefficient (PCC) against CellMask™ Deep Red, which was calculated to 0.85 for the whole image (Fig. 8D); and (ii) the good alignment between the emission profiles of the two different probes (Fig. 8E).

Colocalisation experiments were also performed with analogues 3c and 3d after irradiation with light (Fig. S41 and S42†). The substantial decrease in the PCCs (0.29 and 0.25, respectively) corroborates that the fluorescence was non-specifically localised within the NK cells and accounts for the efficient uptake of coumarin-caged inhibitors. These results further support the occurrence of the photoinduced release of the LCK inhibitor 1 from compounds 3c and 3d in the subcellular milieu as well as the observed light-mediated cellular activity when employing these caged derivatives.

As expected, NK cells incubated with 3c and 3d and kept in the dark (*i.e.* no decaging) display emission intensities that were substantially lower compared to those detected in cells that were irradiated with UV light for decaging (Fig. S46–S47 *vs.* S38–S39†). Interestingly, this observation does not apply to compound 3e, where the emission levels are similar regardless of whether cells were irradiated with light or kept in the dark (Fig. S48 *vs.* S40†). The Corrected Total Cell Fluorescence (CTCF) plot, which summarizes all different explored scenarios (Fig. S55†), illustrates these findings. Moreover, the subcellular localisation of all compounds was not affected by the exposure to light. That is, under dark conditions, compounds 3c and 3d were non-specifically distributed throughout the cytoplasm, while 3e was entrapped within the cell membrane. This was further corroborated by the visual inspection of the images together with the calculated PCCs against the commercial cell membrane stain (0.31, 0.29, and 0.79 for 3c, 3d, and 3e, respectively; for further details see Fig. S49–S51†).

The fact that similar emission intensities were observed in both scenarios (with and without irradiation at 523 nm) for 3e further supports the notion that photo-induced decaging of compound 3e is not occurring. The decaging mechanism for BODIPY-derived caging groups is reliant on the presence of water to react with the BODIPY cation that forms as a result of photo-induced heterolytic cleavage.^39^ Thus, it was anticipated that the accumulation of 3e in the hydrophobic environment of the membrane prevents the release of the LCK inhibitor 1 when irradiated with light. Furthermore, this explains the lack of biological activity for irradiated 3e in the functional cellular assays. This hypothesis can be further supported by recent studies utilising coumarin-^40–42^ and xanthenium-derived^43^ caging groups, which also undergo the same S_N_1-type decaging mechanism observed for BODIPY derivatives. These recent studies demonstrate the effect of water content on decaging efficiency, in which higher water content results in increased photolysis quantum yields. Such results suggest that one needs to consider the polarity of the intended environment where photolysis is to be performed. In cases where photolysis is to occur in a hydrophobic environment, caging groups that are not reliant on the presence of water for their decaging mechanism, such as *o*-nitrobenzyl^44,45^ and tertiary coumarin^40^ variants, should be considered.

Negative control NK cells, *i.e.*, without any compound treatment, did not show significant background fluorescence when either irradiated with light or kept in the dark (Fig. S43–S45 and S52–S54†).

## Conclusions

The development of release-and-report LCK inhibitors that exhibit ‘OFF–ON’ fluorescent changes in concert with the release and subsequent binding of the LCK inhibitor to the enzyme are described. This has been achieved through the introduction of photolabile caging groups onto a solvatochromic LCK inhibitor. Fluorescence titration experiments of caged inhibitor 3e with LCK demonstrate that the decaging process can be readily monitored by an increase in emission intensity observed from the caging group, while a diagnostic increase in the emission intensity and a blue-shift of the emission maxima of the ‘active’ LCK inhibitor report its binding to the LCK enzyme.

Employing a functional assay whereby NK cells are dependent on NKG2D signalling, photo-induced LCK inhibition was investigated. It was observed that the nature of the caging group influenced cellular uptake and subcellular localisation prior to the release of the ‘active’ LCK inhibitor 1. Coumarin-derived caging groups demonstrated non-specific intracellular localisation and light-dependent LCK inhibition, while the use of a BODIPY-derived caging group resulted in the selective accumulation within the cell membrane and poor photo-induced cellular activity. It is anticipated that the hydrophobic environment of the cell membrane hinders the cellular uptake of 3e, and in turn, the occurrence of efficient decaging within live cells.

Given the need to further our understanding of the role of LCK in both health and disease, it is envisioned that the caged LCK inhibitors will serve as valuable molecular probes to manipulate LCK signalling with high spatiotemporal control. While we have demonstrated the concept of release-and-report inhibitors on kinase enzymes, this approach has the potential to be extended to other biorelevant targets where spatiotemporal control in a cellular setting would be beneficial.

## Data availability

The data that support the findings of this study are available from the corresponding author upon reasonable request.

## Author contributions

C. L. F. conducted all the experimental work regarding the synthesis, photophysical characterisation and titration experiments. C. B.-M. performed all the work for the cyclic voltammetry and fluorescence bioimaging experiments. E. B. and L. K. conducted the cellular experiments. Y. X. performed the cell-free biochemical assays. C. L. F., C. B.-M. and E. B. wrote the manuscript. All authors analysed the experimental data and discussed the results. M. G. and J. A. directed the project.

## Conflicts of interest

There are no conflicts to declare.

## Supplementary Material

SC-015-D4SC00390J-s001
