# Supplementary material for: All-photonic kinase inhibitors: light-controlled release-and-report inhibition
Source: Chem Sci. 2024 Apr 12;15(18):6897–905. doi: 10.1039/d4sc00390j (PMC11077529; doi:10.1039/d4sc00390j)
Supplement: SC-015-D4SC00390J-s001 [file SC-015-D4SC00390J-s001.pdf]

Electronic Supplementary Material (ESI) for Chemical Science.
This journal is © The Royal Society of Chemistry 2024

Table of Contents

General Experimental Information for Synthesis and Compound Characterisation 2

Detailed Synthetic Procedure and Characterisation of Compounds **4a**, **4b** and **3c–e** 3

^{1}H and ^{13}C NMR Spectra of Compounds **4a**, **4b** and **3c–e** 20

Photophysical Characterisation of Compounds **4a**, **4b** and **3c–e** 48

Monitoring Decaging Reactions using UV/vis Absorption Spectroscopy 49

Photostability of Compound **1** 51

Thermal Stability of Compounds **1**, **4a**, **4b** and **3c–e** in Aqueous Solution and DMSO 54

Absorption and Emission Spectra of Compounds **3c–e** 60

Spectroscopic Data for Decaged Products **S12**, **S16** and **S20** 61

Absorption and Emission Spectra of Compounds **S12**, **S16** and **S20** 62

Cyclic Voltammetry 63

Monitoring Decaging Reactions using Fluorescence Spectroscopy 64

Monitoring Decaging Reactions using HPLC 66

IC_{50} Determination of LCK Inhibitor **1** and Caged Inhibitors **3c–e** 68

Titration Experiments 71

Cellular Experiments 71

Fluorescence Bioimaging Experiments 74

NK Cells Irradiated with Light 74

NK Cells Not Irradiated with Light 78

References 83

General Experimental Information for Synthesis and Compound Characterisation

General reagents and solvents for the synthesis of compounds were purchased from commercial sources and used as supplied, unless otherwise stated. When anhydrous THF was required, THF was distilled over Na/benzophenone. Those reactions that employed microwave irradiation were conducted using a Biotage Initiator™ reactor with fixed hold time. Purification by flash column chromatography was performed on a Biotage SP4 Flash® instrument using prefabricated silica columns. All NMR spectra (^{1}H and ^{13}C) were collected on either a Bruker Ascend 400 MHz spectrometer or a Varian 400 MHz spectrometer at 25 °C. Samples were dissolved (0.5 mL) in either deuterated chloroform ($CDCl_3$) or deuterated dimethyl sulfoxide (DMSO-d_6). The residual solvent peaks specific to that to the deuterated solvent was used as an internal reference; $CDCl_3$: 7.26 ppm (^{1}H NMR) and 77.20 ppm (^{13}C NMR), DMSO-d_6: 2.50 ppm (^{1}H NMR) and 39.52 ppm (^{13}C NMR). NMR shifts were assigned using 2D NMR experiments (COSY, HSQC and HMBC). High Resolution Mass Spectra (HRMS) was performed on an Agilent 1290 Infinity LC system equipped with autosampler tandem to an Agilent 6520 Accurate Mass Q-TOF LC/MS with ESI ionisation source.

Compound **1** was prepared according to literature previously reported by Fleming *et. al*.[1]

Methods used for the purification of products obtained from the carbamate formation.

Purification of carbamate substrates (**3a–e**) were performed in two stages according to the methods listed below:

Method A. Firstly, crude material was purified *via* flash column chromatography using $CHCl_3$ as the eluent. The desired product co-eluted with the *p*-nitrophenol by product, therefore further purification using amine functionalised silica (1:1 CH_2Cl_2/pentane) afforded the desired product.

Method B. Firstly, crude material was purified *via* flash column chromatography using amine functionalised silica and CH_2Cl_2 as the eluent. The desired product co-eluted with the *p*-nitrophenol by product, therefore further purification using *via* flash column chromatography using $CHCl_3$ as the eluent afforded the desired product.

Method C. Firstly, crude material was purified *via* flash column chromatography using $CHCl_3$ as the eluent. The desired product co-eluted with the *p*-nitrophenol by product, therefore further purification using amine functionalised silica (1:1 $CHCl_3$/pentane) afforded the desired product.

Method D. Firstly, crude material was purified *via* flash column chromatography using $CHCl_3$ as the eluent. The desired product co-eluted with the *p*-nitrophenol by product, therefore further purification using amine functionalised silica (80% $CHCl_3$ in pentane) afforded the desired product.

Detailed Synthetic Procedure and Characterisation of Compounds 4a, 4b and 3c–e.

Synthesis of compound 4a:

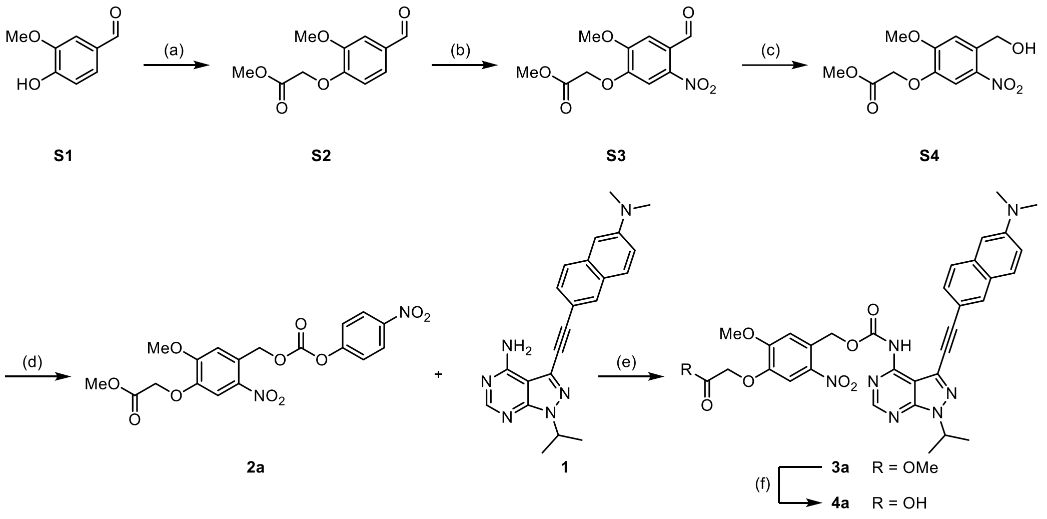

Reagents and Conditions**:** (a) methyl chloroacetate, K_2CO_3, NaI, acetone, 56 °C, 22 h; (b) HNO_3, CH_3CO_3H, 21 °C, 20 h; (c) $NaBH_4$, (1:1) MeOH/CH_2Cl_2, 0 °C, 10 mins; (d) *p*-nitrophenol chloroformate, pyridine, CH_2Cl_2, 21 °C, 24 h; (e) HOBt, CH_2Cl_2, 40 °C, 4 days; (f) $LiOH·H_2O$, (1:1) THF/H_2O, 21 °C, 45 mins.

Methyl 2-(4-formyl-2-methoxyphenoxy)acetate (S2)

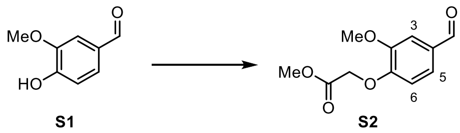

To a suspension of vanillin **S1** (5.00 g, 32.86 mmol) in acetone (anhydrous, 100 mL) were added methyl chloroacetate (3.60 mL, 41.08 mmol), K_2CO_3 (12.00 g, 86.75 mmol) and NaI (395 mg, 2.63 mmol). The resulting reaction mixture was left to stir at 56 °C for 22 hours. The reaction mixture was cooled to 21 °C and excess solvent removed. The crude solid was suspended in H_2O (150 mL) and the precipitate was then collected by vacuum filtration. Purification by recrystallization ($PhCH_3$/Hexane) afforded the title compound **S2** (5.37 g, 73%) as an off-white solid. ^{1}H NMR (400 MHz, $CDCl_3$): δ 9.86 (s, 1H, CHO), 7.45–7.41 (m, 2H, H-3, H-5), 6.87 (d, *J* = 8.1 Hz, 1H, H-6), 4.79 (s, 2H, CH_2CO$_2$CH$_3$), 3.95 (s, 3H, OCH_3), 3.81 (s, 3H, CH$_2$CO$_2$CH_3). ^{13}C NMR (101 MHz, $CDCl_3$): δ 191.0, 168.6, 152.6, 150.1, 131.3, 126.3, 112.4, 110.0, 66.0, 56.2, 52.6. Data is in agreement with the literature.[2]

Methyl 2-(4-formyl-2-methoxy-5-nitrophenoxy)acetate (S3)

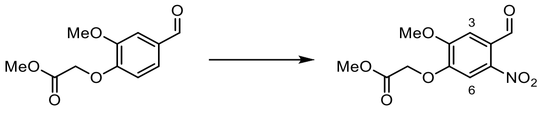

A solution of fuming HNO_3 (2.90 mL) and acetic acid (glacial, 11.20 mL) was added dropwise to methyl 2-(4-formyl-2-methoxy-5-nitrophenoxy)acetate **S2** (1.08 g, 4.82 mmol) at 0 °C. After stirring at 21 °C for 20 hours, the reaction mixture was poured over ice. The resulting aqueous phase was extracted with EtOAc (3 × 50 mL). Combined organic layer was washed with $NaHCO_3$ (sat. solution, 2 × 20 mL), dried ($MgSO_4$), filtered and excess solvent removed to afford the title compound **S3** (1.00 g, 78%) as a pale yellow solid. The product was used without further purification. ^{1}H NMR (400 MHz, $CDCl_3$): δ 10.46 (s, 1H, CHO), 7.54 (s, 1H, H-6), 7.45 (s, 1H, H-3), 4.85 (s, 2H, C*H*$_2$$CO_2CH_3$), 4.04 (s, 3H, OCH_3), 3.84 (s, 3H, CH_2CO_2C*H*$_3$). ^{13}C NMR (101 MHz, $CDCl_3$): δ 187.8, 167.8, 153.8, 150.4, 143.3*, 126.9, 110.8, 109.2, 66.1, 57.0, 52.9. HRMS (ESI/Q-TOF) *m/z*: $[M + H]^+$ Calcd for $C_{11}H_{12}NO_7$ 270.0608; Found: 270.0612. **C5 was identified by HMBC.*

Methyl 2-(4-(hydroxymethyl)-2-methoxy-5-nitrophenoxy)acetate (S4)

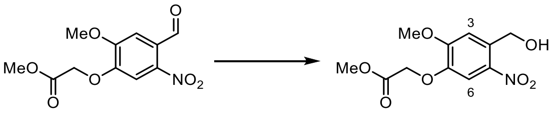

To a solution of methyl 2-(4-formyl-2-methoxy-5-nitrophenoxy)acetate **S3** (854 mg, 3.17 mmol) in MeOH/CH_2Cl_2 (1:1, 20 mL) at 0 °C, was added $NaBH_4$ (57 mg, 1.52 mmol), portion-wise. The reaction was monitored by TLC and after stirring at 0 °C for 10 minutes all starting materials had been consumed. The reaction mixture was then poured onto ice and the pH adjusted to pH 2 using 0.1 M HCl. The aqueous phase was extracted with $CHCl_3$ (3 × 20 mL). Combined organic phase was dried ($MgSO_4$), filtered and excess solvent removed. Trituration with CH_2Cl_2 afforded the title compound **S4** (504.6 mg, 59%) and a pale yellow solid. ^{1}H NMR (400 MHz, $CDCl_3$): δ 7.66 (s, 1H, H-6), 7.24 (s, 1H, H-3), 4.98 (s, 2H, C*H*$_2$OH), 4.77 (s, C*H*$_2$$CO_2CH_3$), 4.02 (s, 3H, OCH_3), 3.83 (s, 3H, CH_2CO_2C*H*$_3$). ^{13}C NMR (101 MHz, $CDCl_3$): δ 168.5, 154.5, 146.0, 139.5, 133.9, 111.6, 110.7, 66.2, 62.8, 56.7, 52.7. HRMS (ESI/Q-TOF) *m/z*: $[M + Na]^+$ Calcd for $C_{11}H_{13}NO_7Na$ 294.0584; Found: 294.0593.

Carbonate 2a

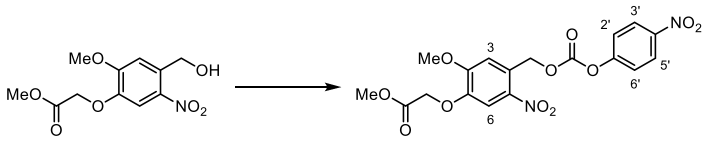

To a solution of 4-nitrophenyl chloroformate (541 mg, 2.68 mmol) in CH_2Cl_2 (4 mL) at 0 °C was added pyridine (223 µL, 2.76 mmol). After stirring at 0 °C for 20 minutes, a suspension of methyl 2-(4-methoxy-5-nitrophenoxy)acetate **S4** (497 mg, 1.84 mmol) in CH_2Cl_2 (4 mL) was slowly added and the resulting reaction mixture was allowed to stir at 21 °C for 24 hours. The reaction mixture was washed with HCl (2 M, 3 × 10 mL) and brine (10 mL). The organic layer was dried ($MgSO_4$), filtered and excess solvent removed. Purification by flash column chromatography (1:1 EtOAc/Pentane) afforded the title compound **2a** (403 mg, 50%, R_f = 0.37) as an off-white solid. The product was used without further purification. ^{1}H NMR (400 MHz, $CDCl_3$): δ 8.27 (d, J = 9.1 Hz, 2H, H-3′, H-5′), 7.69 (s, 1H, H-6), 7.40 (d, J = 9.1 Hz, 2H, H-2′, H-6′), 7.14 (s, 1H, H-3), 5.69 (s, 2H, CH_2), 4.78 (s, 2H, $CH_2CO_2CH_3$), 4.02 (s, 3H, OCH_3), 3.82 (s, 3H, $CH_2CO_2CH_3$). ^{13}C NMR (101 MHz, $CDCl_3$): δ 168.3, 155.4, 154.3, 152.2, 146.9, 145.7, 139.7, 126.7, 125.5 (CH × 2), 121.9 (CH × 2), 111.3, 110.8, 67.7, 66.1, 56.8, 52.7. HRMS (ESI/Q-TOF) m/z: $[M + Na]^+$ Calcd for $C_{18}H_{16}N_2O_{11}Na$ 459.0646; Found: 459.0644.

Carbamate 3a

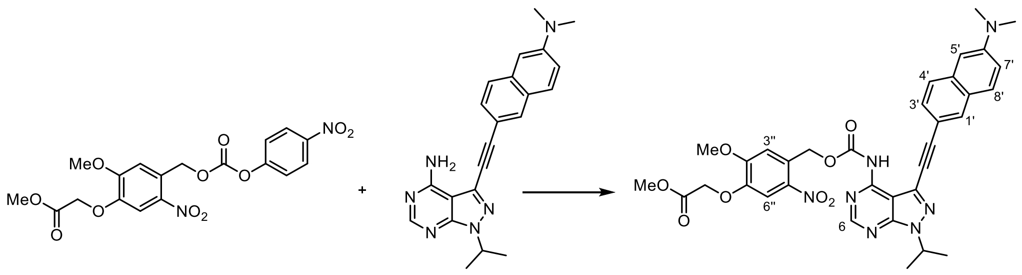

To a solution of compound **1** (104 mg, 0.281 mmol) in CH_2Cl_2 (anhydrous, 2 mL), was added carbonate **2a** (123 mg, 0.281 mmol) and HOBt (38 mg, 0.281 mmol). The resulting reaction mixture was left to at 40 °C for 4 days. Upon cooling to 21 °C, excess solvent was removed. Purification by flash column chromatography ($CHCl_3$) gave an orange residue. Trituration in CH_3CN at 0 °C afforded the title compound **3a** (26 mg, 14%, R_f = 0.23 in 1:40 MeOH/$CHCl_3$) as a yellow solid. ^{1}H NMR (400 MHz, $CDCl_3$): δ 8.77 (s, 1H, H-6), 8.63 (s, 1H, NH), 7.99 (br s, 1H, H-1′), 7.65 (s, 1H, H-6″), 7.64–7.54 (m, 2H, H-4′, H-8′), 7.49 (dd, J = 8.6, 1.4 Hz, 1H, H-3′), 7.23 (s, 1H, H-3″), 7.14 (dd, J = 9.1, 2.2 Hz, 1H, H-7′), 6.85 (d, J = 2.2 Hz, 1H, H-5′), 5.77 (s, 2H, CH_2), 5.22 (sept, J = 6.7 Hz, 1H, $CH(CH_3)_2$), 4.72 (s, 2H, $CH_2CO_2CH_3$), 3.80 (s, 3H, $CH_2CO_2CH_3$), 3.77 (s, 3H, OCH_3), 3.09 (s, 6H, $N(CH_3)_2$), 1.62 (d, J = 6.7 Hz, 6H, $CH(CH_3)_2$). ^{13}C NMR (101 MHz, $CDCl_3$): δ 168.3, 156.2, 154.2, 152.8, 152.6, 150.2, 149.7, 146.6, 139.9, 135.4, 132.2, 129.1, 128.2, 127.7, 126.8, 125.8, 125.7, 116.8, 113.3, 112.1, 110.8, 105.8,

103.9, 97.8, 79.7, 66.2, 65.1, 56.5, 52.7, 50.1, 40.7 (CH_3 × 2), 22.2 (CH_3 × 2). HRMS (ESI/Q-TOF) *m/z*: $[M + H]^+$ Calcd for $C_{34}H_{34}N_7O_8$ 668.2463; Found: 668.2469.

Carbamate 4a

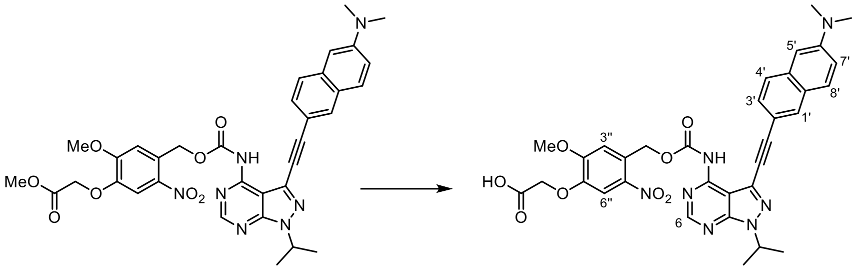

3a **4a**

To a suspension of carbamate **3a** (43 mg, 0.064 mmol) in THF:H_2O (1:1, 4 mL), was added LiOH·H_2O (6 mg, 0.150 mmol). After stirring at 21 °C for 45 minutes, excess solvent was removed. The resulting yellow residue was suspended in H_2O (5 mL) and the pH adjusted to pH 3 using HCl (0.1 M). The resulting aqueous phase was then extracted with EtOAc (3 × 5 mL). Combined organic layer was then dried ($MgSO_4$), filtered and excess solvent removed. Trituration in CH_3CN afforded the title compound **4a** (11 mg, 26%) as a pale yellow solid. ^1H NMR (400 MHz, DMSO-d_6): δ 13.17 (br s, 1H, CH_2CO_2H), 10.99 (br s, 1H, OCONH), 8.79 (s, 1H, H-6), 7.87 (s, 1H, H-1′), 7.58–7.48 (m, 3H, H-4′, H-8′, H-6″), 7.36 (d, *J* = 9.0 Hz, 1H, H-3′), 7.25–7.11 (m, 2H, H-7′, H-3″), 6.90 (d, *J* = 2.5 Hz, 1H, H-5′), 5.49 (s, 2H, CH_2OCONH), 5.23–5.13 (m, 1H, $CH(CH_3)_2$), 4.80 (s, 2H, CH_2CO_2H), 3.79 (s, 3H, OCH_3), 3.04 (s, 6H, $N(CH_3)_2$), 1.53 (d, *J* = 6.7 Hz, 6H, $CH(CH_3)_2$). ^{13}C NMR (101 MHz, DMSO-d_6): δ 169.5, 155.3*, 153.5, 153.2, 152.8†, 149.3, 146.1, 138.7, 134.6, 131.2, 128.5, 127.7, 127.1, 126.3, 125.2, 116.7, 113.3, 110.6, 109.5, 105.2, 94.5, 81.0, 65.2, 64.0, 56.2, 49.3, 39.8 (CH_3 × 2)*, 21.7 (CH_3 × 2). HRMS (ESI/Q-TOF) *m/z*: $[M + H]^+$ Calcd for $C_{33}H_{32}N_7O_8$ 654.2307; Found: 654.2335. * *Identified by HSQC.* † *Identified by HMBC. Carbons from the pyrazolo[3,4-d]pyrimidine heterocyclic core were not identified in the ^{13}C NMR spectrum. It is thought that the absence of these carbons is the result of quadrupolar broadening.*

Synthesis of compound 4b:

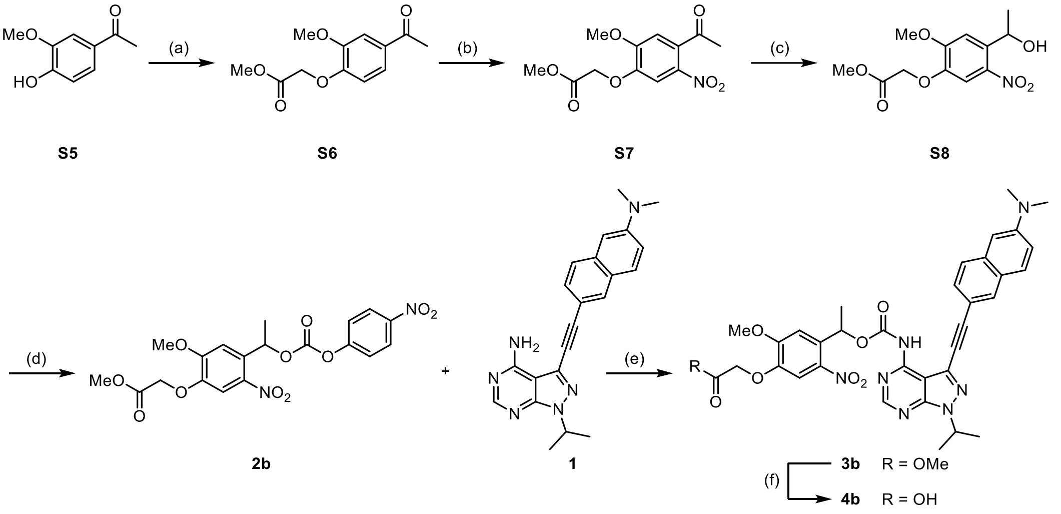

***Reagents and Conditions*:** (a) methyl chloroacetate, K_2CO_3, NaI, acetone, 56 °C, 16 h; (b) HNO_3, Ac_2O, –40 °C, 30 mins; (c) $NaBH_4$, (1:1) MeOH/CH_2Cl_2, 0–21 °C, 2.5 h; (d) *p*-nitrophenol chloroformate, pyridine, CH_2Cl_2, 40 °C, 48 h; (e) HOBt, CH_2Cl_2, 40 °C, 5 days; (f) LiOH·H_2O, (1:1) THF/H_2O, 21 °C, 45 mins.

Methyl 2-(4-acetyl-2-methoxyphenoxy)acetate (S6)

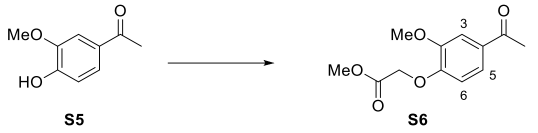

To a suspension of 4′-hydroxy-3′-methoxyacetophenone **S5** (651 mg, 3.92 mmol) in acetone (7 mL) were added methyl chloroacetate (430 µL, 4.90 mmol), K_2CO_3 (1.63 g, 11.76 mmol) and NaI (470 mg, 3.14 mmol). The resulting reaction mixture was left to stir at 56 °C for 16 hours. The reaction mixture was cooled to 21 °C and excess solvent removed. The crude solid was suspended in H_2O (150 mL) and the precipitate was then collected by vacuum filtration to afford the title compound **S6** (751 mg, 81%) as an off-white solid. The product was used without further purification. ^1H NMR (400 MHz, $CDCl_3$): δ 7.56–7.49 (m, 2H, H-3, H-5), 6.79 (d, *J* = 8.3 Hz, 1H, H-6), 4.77 (s, 2H, $CH_2CO_2CH_3$), 3.94 (s, 3H, OCH_3), 3.80 (s, 3H, $CH_2CO_2CH_3$), 2.56 (s, 3H, $COCH_3$). ^{13}C NMR (101 MHz, $CDCl_3$): δ 196.9, 168.9, 151.4, 149.5, 131.8, 122.9, 112.1, 111.0, 66.0, 56.2, 52.6, 26.4. HRMS (ESI/Q-TOF) *m/z*: $[M + Na]^+$ Calcd for $C_{12}H_{14}O_5Na$ 239.0924; Found 239.0926.

Methyl 2-(4-acetyl-2-methoxy-5-nitrophenoxy)acetate (S7)

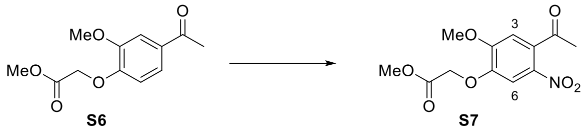

To a solution of HNO_3 (6.50 mL) cooled to 0 °C, was added acetic anhydride (10 mL). This was left to stir at 0 °C for 30 minutes. To a suspension of methyl 2-(4-acetyl-2-methoxyphenoxy)acetate **S6** (1.15 g, 4.81 mmol) in acetic anhydride (10 mL) at – 40 °C was added the acetyl nitrate solution in a dropwise manner. After stirring at – 40 °C for 30 minutes the reaction mixture was poured onto ice. The resulting aqueous phase was then extracted with $CHCl_3$ (3 × 50 mL). The combined organic layer was washed with H_2O (20 mL) and $NaHCO_3$ (sat., 20 mL), dried ($MgSO_4$), filtered and excess solvent removed to afford the title compound **S7** (903 mg, 66%) as a pale yellow solid. The product was used without further purification. ^{1}H NMR (400 MHz, $CDCl_3$): δ 7.55 (s, 1H, H-6), 6.78 (s, 1H, H-3), 4.79 (s, 2H, $C\mathit{H_2}CO_2CH_3$), 3.99 (s, 3H, OCH_3), 3.83 (s, 3H, $CH_2CO_2C\mathit{H_3}$), 2.50 (s, 3H, $COCH_3$). ^{13}C NMR (101 MHz, $CDCl_3$): δ 200.0, 168.1, 154.6, 147.6, 138.1, 134.3, 109.3, 109.2, 66.1, 56.9, 52.8, 30.6. HRMS (ESI/Q-TOF) *m/z*: $[M + H]^+$ Calcd for $C_{12}H_{14}NO_7$ 284.0765; Found 284.0774.

Methyl 2-(4-(1-hydroxyethyl)-2-methoxy-5-nitrophenoxy)acetate (S8)

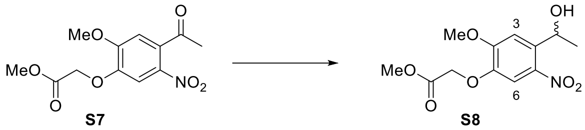

To a solution of methyl 2-(4-acetyl-2-methoxy-5-nitrophenoxy)acetate **S7** (210 mg, 0.716 mmol) in MeOH:CH_2Cl_2 (1:1, 10 mL) at 0 °C, was added $NaBH_4$ (54 mg, 1.43 mmol), portion-wise. After stirring at 0 °C for 30 minutes the reaction mixture was allowed to warm to 21 °C and was continued to stir for a further 2 hours. The reaction mixture was then poured onto ice and the pH adjusted to pH 3 using 0.1 M HCl. The aqueous phase was extracted with $CHCl_3$ (3 × 20 mL). Combined organic phase was dried ($MgSO_4$), filtered and excess solvent removed. Purification by flash column chromatography (1:1 EtOAc/Pentane) afforded the title compound **S8** (102 mg, 50%, R_f = 0.29) as a light brown solid. ^{1}H NMR (400 MHz, $CDCl_3$): δ 7.52 (s, 1H, H-6), 7.35 (s, 1H, H-3), 5.57 (q, J = 6.3 Hz, 1H, $C\mathit{H}CH_3$), 4.75 (s, 2H, $C\mathit{H_2}CO_2CH_3$), 4.01 (s, 3H, OCH_3), 3.82 (s, 3H, $CH_2CO_2C\mathit{H_3}$), 2.27 (br s, 1H, OH), 1.55 (d, J = 6.3 Hz, 3H, $CHC\mathit{H_3}$). ^{13}C NMR (101 MHz, $CDCl_3$): δ 168.6, 154.3, 145.8, 139.4, 138.5, 110.3, 109.4, 66.2, 65.9, 56.6, 52.6, 24.4. HRMS (ESI/Q-TOF) *m/z*: $[M + Na]^+$ Calcd for $C_{12}H_{15}NO_7Na$ 308.0741; Found 308.0751.

Carbonate 2b

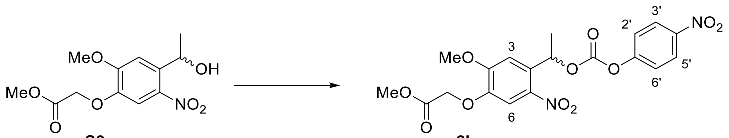

S8 **2b**

To a solution of 4-nitrophenyl chloroformate (284 mg, 1.41 mmol) in CH_2Cl_2 (2 mL) at 0 °C was added pyridine (114 µL, 1.41 mmol). After stirring at 0 °C for 20 minutes, a solution of methyl 2-(4-methoxy-5-nitrophenoxy)acetate **S8** (268 mg, 0.940 mmol) in CH_2Cl_2 (2 mL) was slowly added and the resulting reaction mixture was allowed to stir at 40 °C for 48 hours. The reaction mixture was washed with HCl (2 M, 3 × 5 mL) and brine (5 mL). The organic layer was dried ($MgSO_4$), filtered and excess solvent removed. Purification by column chromatography (1:1 EtOAc/Pentane) afforded the title compound **2b** (306 mg, 72%, R_f = 0.59) as colourless oil. ^{1}H NMR (400 MHz, $CDCl_3$): δ 8.26 (d, *J* = 9.3 Hz, 2H, H-3′, H-5′), 7.55 (s, 1H, H-6), 7.35 (d, *J* = 9.3 Hz, 2H, H-2′, H-6′), 7.16 (s, 1H, H-3), 6.54 (q, *J* = 6.5 Hz, 1H, C*H*CH_3), 4.78 (s, 2H, C*H*$_2CO_2CH_3$), 4.03 (s, 3H, OCH_3), 3.83 (s, 3H, CH_2CO_2C*H*$_3$), 1.77 (d, *J* = 6.4 Hz, 3H, CHC*H*$_3$). ^{13}C NMR (101 MHz, $CDCl_3$): δ 168.3, 155.4, 154.4, 151.5, 146.6, 145.5, 139.7, 132.8, 125.4 (CH × 2), 121.8 (CH × 2), 110.1, 108.7, 73.8, 66.0, 56.8, 52.7, 22.1. HRMS (ESI/Q-TOF) *m/z*: $[M + Na]^+$ Calcd for $C_{19}H_{18}N_2O_{11}Na$ 473.0803; Found: 473.0809.

Carbamate 3b

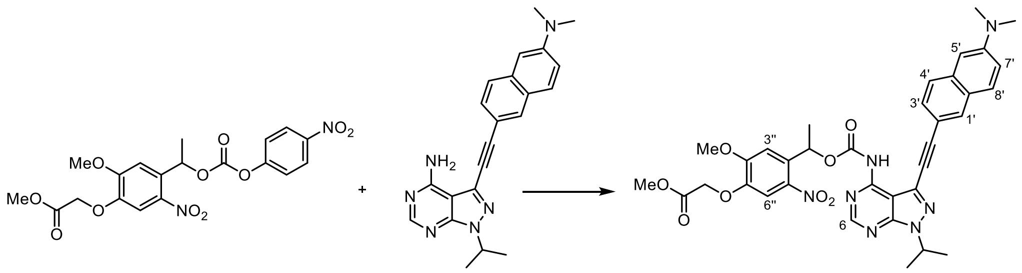

2b **1** **3b**

To a solution of compound **1** (78 mg, 0.211 mmol) in CH_2Cl_2 (anhydrous, 2 mL), was added carbonate **2b** (114 mg, 0.253 mmol) and HOBt (34 mg, 0.253 mmol). The resulting reaction mixture was left to at 40 °C for 5 days. Upon cooling to 21 °C, excess solvent was removed. Purification by flash column chromatography (*Method A*) afforded the title compound **3b** (105 mg, 74%, R_f = 0.17 in $CHCl_3$) as a yellow solid. ^{1}H NMR (500 MHz, $CDCl_3$): δ 8.73 (s, 1H, H-6), 8.63 (s, 1H, OCONH), 8.05 (s, 1H, H-1′), 7.67 (d, *J* = 9.1 Hz, 1H, H-8′), 7.65–7.56 (m, 2H, H-3′, H-4′), 7.50 (s, 1H, H-6″), 7.17 (dd, *J* = 9.1, 2.3 Hz, 1H, H-7′), 7.05 (s, 1H, H-3″), 6.86 (d, *J* = 2.3 Hz, 1H, H-5′), 6.69 (q, *J* = 6.4 Hz, 1H, C*H*CH_3), 5.21 (sept, *J* = 6.7 Hz, 1H, C*H*$(CH_3)_2$), 4.69 (d, *J* = 2.9 Hz, 2H, C*H*$_2CO_2CH_3$), 3.79 (s, 3H, CH_2CO_2C*H*$_3$), 3.50 (s, 3H, OCH_3), 3.10 (s, 6H, $N(CH_3)_2$), 1.79 (d, *J* = 6.4 Hz, 3H, CHC*H*$_3$), 1.61 (dd, *J* = 6.7, 4.4 Hz, 6H, CH(C*H*$_3$)$_2$). ^{13}C NMR (126 MHz, $CDCl_3$): δ 168.4, 156.3, 154.3, 152.8, 152.6, 149.7, 149.3, 146.2, 139.7, 135.5, 134.1, 132.0, 129.0, 128.1, 126.7, 125.8, 125.5, 117.0, 113.4, 110.2, 108.7, 105.7, 104.0,

97.7, 80.0, 70.7, 66.1, 56.2, 52.6, 50.1, 40.6 (CH_3 × 2), 22.4, 22.2 (CH_3 × 2). HRMS (ESI/Q-TOF) *m/z*: $[M + H]^+$ Calcd for $C_{35}H_{36}N_7O_8$ 682.2620; Found: 682.2639.

Carbamate 4b

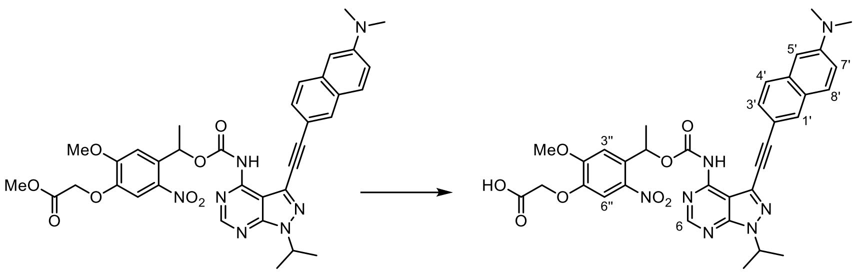

To a suspension of carbamate **3b** (120 mg, 0.176 mmol) in THF/H_2O (1:1, 7 mL), was added LiOH·H_2O (17 mg, 0.410 mmol). After stirring at 21 °C for 45 minutes, the reaction mixture was poured onto ice/H_2O (*ca* 15 mL) and the pH adjusted to pH 3 using HCl (2 M). The resulting aqueous phase was then extracted with EtOAc (3 × 20 mL). Combined organic layer was then washed with brine (10 mL), dried ($MgSO_4$), filtered and excess solvent removed, to afford the title compound **4b** (93 mg, 79%) as a pale yellow solid. ^{1}H NMR (400 MHz, DMSO-d_6): δ 10.81 (s, 1H, OCONH), 8.75 (s, 1H, H-6), 7.93 (s, 1H, H-1′), 7.70 (d, *J* = 9.3 Hz, 1H, H-8′), 7.65 (d, *J* = 8.7 Hz, 1H, H-4′), 7.48 (s, 1H, H-6″), 7.39 (d, *J* = 9.5 Hz, 1H, H-3′), 7.28 (dd, *J* = 9.3, 2.7 Hz, 1H, H-7′), 7.16 (s, 1H, H-3″), 6.96 (d, *J* = 2.7 Hz, 1H, H-5′), 6.32 (q, *J* = 6.5 Hz, 1H, C*H*CH_3), 5.19–5.13 (m, 1H, C*H*$(CH_3)_2$), 4.79 (s, 2H, C*H*$_2CO_2H$), 3.82 (s, 3H, OCH_3), 3.06 (s, 6H, $N(CH_3)_2$), 1.52–1.49 (m, 9H, 3H, CHC*H*$_3$, CH(C*H*$_3)_2$). ^{13}C NMR (101 MHz, DMSO-d_6): δ 169.5, 155.4*, 153.6, 153.2, 149.3, 146.1, 138.8, 134.7, 132.7†, 131.2, 128.6, 127.7, 126.3, 125.2, 116.8, 113.3, 108.9, 108.8, 105.3, 80.9, 69.4*, 65.2, 56.2, 49.3, 40.1 (CH_3 × 2)*, 21.7, 21.6 (CH_3 × 2). HRMS (ESI/Q-TOF) *m/z*: $[M + H]^+$ Calcd for $C_{34}H_{34}N_7O_8$ 668.2463; Found: 668.2456. * *Identified by HSQC.* † *Identified by HMBC. Carbons from the pyrazolo[3,4-d]pyrimidine heterocyclic core and one carbon from the alkyne were not identified in the ^{13}C NMR spectrum. It is thought that the absence of these carbons is the result of quadrupolar broadening.*

Synthesis of compound 3c:

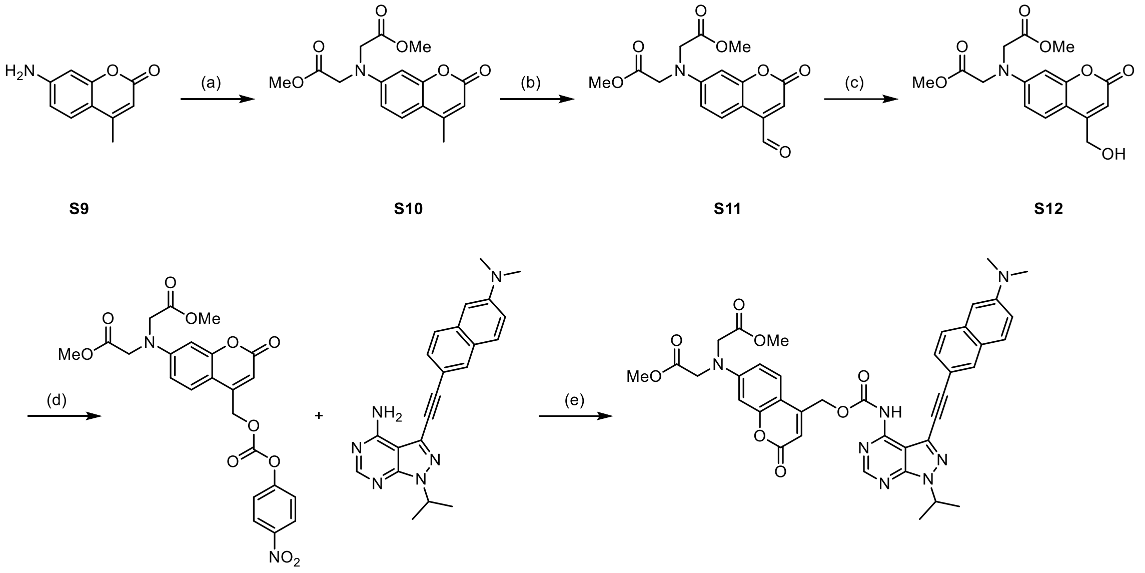

Reagents and Conditions**:** (a) $BrCH_2COOCH_3$, NaI, DIPEA, CH_3CN, 82 °C, 4 days; (b) SeO_2, *p*-xylene, 138 °C, 16 h; (c) $NaBH_4$, THF, 21 °C, 3.5 h; (d) *p*-nitrophenyl chloroformate, pyridine, CH_2Cl_2, 40 °C, 48 h; (e) HOBt, CH_2Cl_2, 40 °C, 7 days.

7-(Bis(methoxycarbonylmethyl)amino)-4-methylcoumarin (S10)

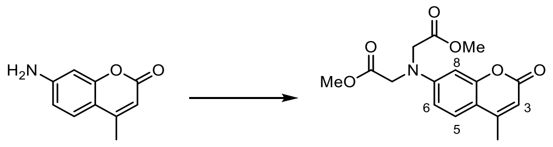

To a suspension of 7-amino-4-methylcoumarin **S9** (756 mg, 4.32 mmol) and NaI (648 mg, 4.32 mmol) in CH_3CN (anhydrous, 20 mL), was added bromoacetic acid methyl ester (4.09 mL, 43.20 mmol) and DIPEA (3.80 mL, 21.82 mmol). The resulting reaction mixture was stirred under nitrogen at 82 °C for 4 days. After cooling to 21 °C, the reaction mixture was filtered and excess solvent removed. The resulting residue was dissolved in EtOAc (50 mL), washed with H_2O (20 mL) and brine (20 mL), dried ($MgSO_4$), filtered and excess solvent removed. Purification by flash column chromatography (1:1 EtOAc/pentane) afforded the title compound **S10** (1.05 g, 76%, R_f = 0.29) as a light brown solid. ^1H NMR (400 MHz, $CDCl_3$): δ 7.39 (d, J = 8.9 Hz, 1H, H-5), 6.52 (dd, J = 8.9, 2.7 Hz, 1H, H-6), 6.43 (d, J = 2.7 Hz, 1H, H-8), 5.99 (q, J = 1.2 Hz, 1H, H-3), 4.17 (s, 4H, CH_2 × 2), 3.74 (s, 6H, OCH_3 × 2), 2.31 (d, J = 1.2 Hz, 3H, CH_3). ^{13}C NMR (101 MHz, $CDCl_3$): δ 170.3 (C × 2), 161.6, 155.4, 152.7, 150.7, 125.8, 111.5, 110.7, 109.1, 99.4, 53.3 (CH_2 × 2), 52.5 (CH_3 × 2), 18.5. HRMS (ESI/Q-TOF) *m/z*: $[M + H]^+$ Calcd for $C_{16}H_{18}NO_6$ 320.1129; Found: 320.1133.

7-(Bis(methoxycarbonylmethyl)amino)-4-(hydroxymethyl)coumarin (S12)

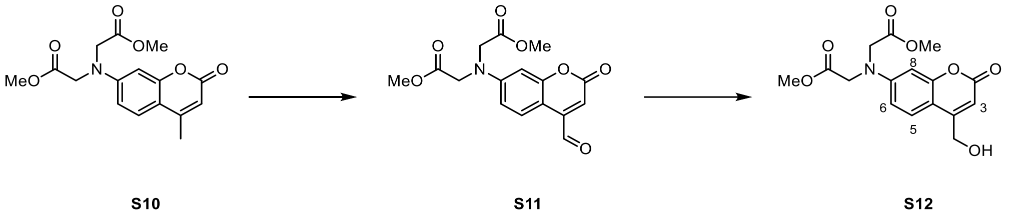

A suspension of 7-(bis(methoxycarbonylmethyl)amino)-4-methylcoumarin **S10** (578 mg, 1.81 mmol) and selenium dioxide (301 mg, 2.72 mmol) in *p*-xylene (10 mL) was heated to 138 °C under a nitrogen atmosphere. After stirring for 16 hours, the reaction mixture was cooled to 21 °C. The reaction mixture was then filtered and excess solvent removed. The resulting brown residue was then dissolved in THF (20 mL), to which $NaBH_4$ (137 mg, 3.62 mmol) was added. After stirring at 21 °C for 3.5 hours, the reaction was quenched with $NaHCO_3$ (sat. solution, 40 mL) and the extracted with CH_2Cl_2 (3 × 20 mL). Combined organic layer was washed with H_2O (20 mL) and brine (20 mL), dried ($MgSO_4$), filtered and excess solvent removed. Purification by flash column chromatography (1:1 EtOAc/CH_2Cl_2) afforded the title compound **S12** (327 mg, 54%, R_f = 0.25) as a dark yellow solid. ^{1}H NMR (400 MHz, DMSO-d_6): δ 7.47 (d, *J* = 8.9 Hz, 1H, H-5), 6.60 (dd, *J* = 8.9, 2.7 Hz, 1H, H-6), 6.53 (d, *J* = 2.7 Hz, 1H, H-8), 6.16 (s, 1H, H-3), 5.54 (t, *J* = 5.6 Hz, 1H, CH_2OH), 4.68 (dd, *J* = 5.6, 1.5 Hz, 2H, CH_2OH), 4.35 (s, 4H, CH_2 × 2), 3.67 (s, 6H, OCH_3 × 2). ^{1}H NMR (400 MHz, $CDCl_3$): δ 7.23 (d, *J* = 8.8 Hz, 1H, H-5), 6.46 (dd, *J* = 8.9, 2.7 Hz, 1H, H-6), 6.42 (d, *J* = 2.6 Hz, 1H, H-8), 6.30 (t, *J* = 1.4 Hz, 1H, H-3), 4.69 (d, *J* = 1.6 Hz, 2H, CH_2OH), 4.17 (s, 4H, CH_2 × 2), 3.77 (s, 6H, OCH_3 × 2). ^{13}C NMR (101 MHz, $CDCl_3$): δ 170.4 (C × 2), 162.0, 155.5, 154.6, 150.7, 124.6, 109.3, 108.9, 107.6, 99.6, 77.2, 60.8 (CH_2 × 2), 53.3 (CH_3 × 2), 52.7. HRMS (ESI/Q-TOF) *m/z*: $[M + H]^+$ Calcd for $C_{16}H_{18}NO_7$ 336.1078; Found: 336.1078.

Carbonate 2c

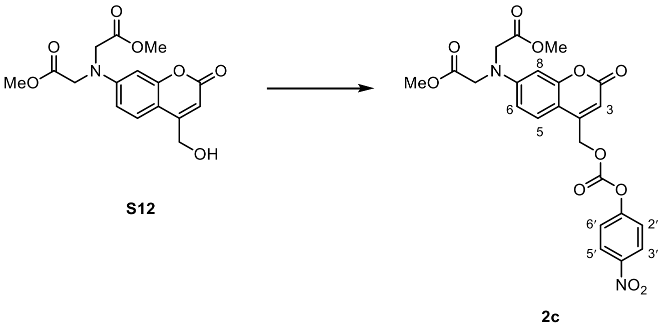

To a solution of 4-nitrophenylchloroformate (314 mg, 1.56 mmol) in CH_2Cl_2 (10 mL) cooled to 0 °C, was added pyridine (125 µL, 1.56 mmol). After stirring at 0 °C for 10 minutes, the resulting white suspension was slowly added to a suspension of 7-(bis(methoxycarbonylmethyl)amino)-4-(hydroxymethyl)coumarin **S12** in CH_2Cl_2 (15 mL) at 21 °C. The resulting reaction mixture was left to stir at 40 °C for 48 hours. Upon cooling to 21 °C, the organic layer was washed with HCl (0.1 M, 3 × 10 mL) and brine (10 mL). The resulting organic layer was then dried ($MgSO_4$), filtered and excess

solvent removed. Purification by flash column chromatography (0–5% MeOH in CH_2Cl_2) afforded to title compound **2c** (397 mg, 76%) as a pale yellow solid and the product was used without further purification. ^1H NMR (400 MHz, $CDCl_3$): δ 8.28 (d, *J* = 9.2 Hz, 2H, H-3′, H-5′), 7.41 (d, *J* = 9.2 Hz, 2H, H-2′, H-6′), 7.37 (d, *J* = 8.9 Hz, 1H, H-5), 6.56 (dd, *J* = 8.9, 2.7 Hz, 1H, H-6), 6.50 (d, *J* = 2.6 Hz, 1H, H-8), 6.31 (t, *J* = 1.3 Hz, 1H, H-3), 5.39 (d, *J* = 1.3 Hz, 2H, CH_2), 4.20 (s, 4H, CH_2 × 2), 3.78 (s, 6H, OCH_3 × 2). ^{13}C NMR (101 MHz, $CDCl_3$): δ 170.1 (C × 2), 161.0, 155.9, 155.3, 152.2, 151.2, 147.6, 145.8, 125.6 (CH × 2), 124.7, 121.9 (CH × 2), 109.6, 109.4, 108.2, 99.9, 65.7, 53.4 (CH_2 × 2), 52.7 (CH_3 × 2). HRMS (ESI/Q-TOF) *m/z*: $[M + H]^+$ Calcd for $C_{23}H_{21}N_2O_{11}$ 501.1140; Found: 501.1148.

Carbamate 3c

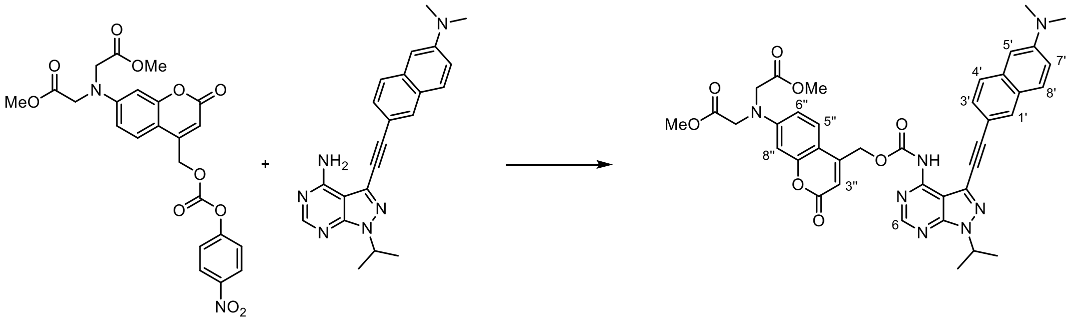

2c 1 3c

To a solution of compound **1** (40 mg, 0.107 mmol) in CH_2Cl_2 (anhydrous, 3 mL), was added carbonate **2c** (64 mg, 0.128 mmol) and HOBt (17 mg, 0.128 mmol). The resulting reaction mixture was left to at 40 °C for 7 days. Upon cooling to 21 °C, excess solvent was removed. Purification by flash column chromatography (*Method C*) afforded the title compound **3c** (36 mg, 46%) as a yellow oil that solidified upon standing. ^1H NMR (400 MHz, $CDCl_3$): δ 8.81 (s, 1H, H-6), 8.64 (s, 1H, NH), 7.95 (br s, 1H, H-1′), 7.60–7.55 (m, 2H, H-4′, H-8′), 7.44 (dd, *J* = 8.5, 1.7 Hz, 1H, H-3′), 7.37 (d, *J* = 8.8 Hz, 1H, H-5″), 7.14 (dd, *J* = 9.1, 2.5 Hz, 1H, H-7′), 6.85 (d, *J* = 2.5 Hz, 1H, H-5′), 6.46 (d, *J* = 2.7 Hz, 1H, H-8″), 6.43 (dd, *J* = 8.8, 2.7 Hz, 1H, H-6″), 6.36 (s, 1H, H-3″), 5.41 (s, 2H, CH_2), 5.23 (sept, *J* = 6.7 Hz, 1H, C*H*(CH_3)$_2$), 4.14 (s, 4H, CH_2 × 2), 3.76 (s, 6H, OCH_3 × 2), 3.08 (s, 6H, $N(CH_3)_2$), 1.62 (d, *J* = 6.7 Hz, 6H, CH(C*H*$_3$)$_2$). ^{13}C NMR (101 MHz, $CDCl_3$): δ 170.1 (C × 2), 161.0, 156.2, 155.8, 152.9, 152.3, 151.0, 149.74, 149.67, 148.4, 135.4, 132.2, 129.0, 128.0, 126.7, 125.8, 125.7, 125.0, 116.9, 113.2, 109.9, 109.5, 108.6, 105.9, 104.1, 99.7, 97.8, 79.7, 63.3, 53.3 (CH_2 × 2), 52.6 (CH_3 × 2), 50.1, 40.6 (CH_3 × 2), 22.1 (CH_3 × 2). HRMS (ESI/Q-TOF) *m/z*: $[M + H]^+$ Calcd for $C_{39}H_{38}N_7O_8$ 732.2776; Found: 732.2776.

Synthesis of 3d:

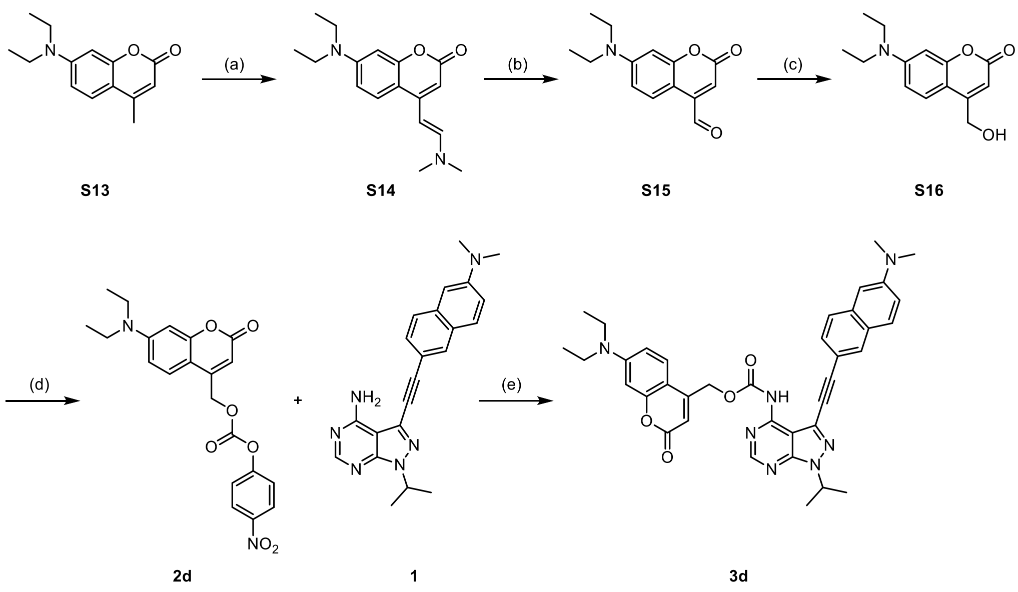

***Reagents and Conditions*:** (a) DMF-DMA, DMF, reflux, 24 h; (b) $NaIO_4$, (1:1) THF/H_2O, 21 °C, 1.5 h; (c) $NaBH_4$, THF, 21 °C, 5 h; (d) *p*-nitrophenol chloroformate, pyridine, CH_2Cl_2, 40 °C, 48 h; (e) HOBt, CH_2Cl_2, 40 °C, 4 days.

(*E*)-7-(Diethylamino)-4-(2-(dimethylamino)vinyl)-2*H*-chromen-2-one (S14)

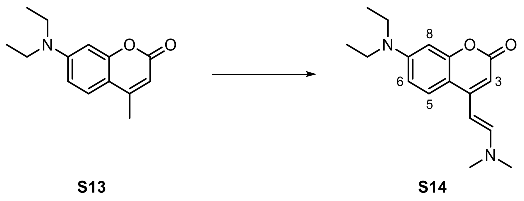

To a solution of 7-diethylamino-4-methylcoumarine **S13** (3.60 g, 15.60 mmol) in DMF (anhydrous, 21 mL) was added DMF-DMA (2.66 mL, 31.14 mmol). The resulting reaction mixture was allowed to reflux for 24 h. Upon cooling to 21 °C, the reaction mixture was diluted with $NaHCO_3$ (sat., 100 mL) and the resulting aqueous phase was then extracted with CH_2Cl_2 (3 × 40 mL). The combined organic layer was dried ($MgSO_4$), filtered and excess solvent removed to afford the title compound **S14** (4.39 g, 98%) as a brown solid. The product was used without further purification. ^{1}H NMR (400 MHz, $CDCl_3$): δ 7.52 (d, *J* = 9.1 Hz, 1H, H-5), 7.21 (d, *J* = 13.0 Hz, 1H, CHC*H*N), 6.54 (dd, *J* = 9.0, 2.7 Hz, 1H, H-6), 6.48 (d, *J* = 2.6 Hz, 1H, H-8), 5.84 (s, 1H, H-3), 5.21 (d, *J* = 13.0 Hz, 1H, C*H*CHN), 3.39 (q, *J* = 7.1 Hz, 4H, N(CH_2CH$_3$)$_2$), 2.98 (s, 6H, N(CH$_3$)$_2$), 1.18 (t, *J* = 7.1 Hz, 6H, N(CH$_2$CH_3)$_2$). ^{13}C NMR (101 MHz, $CDCl_3$): δ 163.5, 156.5, 152.4, 150.2, 146.7, 124.9, 108.2, 108.0, 98.2, 93.4, 87.5, 44.8 (CH_2 × 2), 41.0 (CH_3 × 2), 12.6 (CH_3 × 2). Data is in agreement with the literature.[3]

7-(Diethylamino)-2-oxo-2*H*-chromene-4-carbaldehyde (S15)

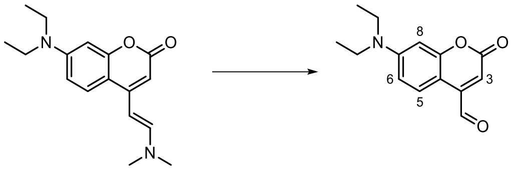

S14 **S15**

To a solution of (*E*)-7-(diethylamino)-4-(2-(dimethylamino)vinyl)-2*H*-chromen-2-one **S14** (4.39 g, 15.33 mmol) in THF/H_2O (1:1, 100 mL), was added $NaIO_4$ (9.83 g, 45.99 mmol). After stirring at 21 °C for 1.5 hours, the precipitate was filtered off, washing thoroughly with EtOAc. The volume of the filtrate was reduced by half under reduced pressure and $NaHCO_3$ (sat., 100 mL) was added. The aqueous phase was extracted with CH_2Cl_2 (3 × 50 mL). Combined organic layer was dried ($MgSO_4$), filtered and excess solvent removed. Purification by flash column chromatography (CH_2Cl_2) afforded the title compound **S15** (3.53 g, 94%, R_f = 0.35) as a red solid. ^{1}H NMR (400 MHz, $CDCl_3$): δ 10.02 (s, 1H, CHO), 8.30 (d, *J* = 9.2 Hz, 1H, H-5), 6.62 (dd, *J* = 9.2, 2.6 Hz, 1H, H-6), 6.51 (d, *J* = 2.6 Hz, 1H, H-8), 6.44 (s, 1H, H-3), 3.42 (q, *J* = 7.1 Hz, 4H, N(CH_2CH$_3$)$_2$), 1.21 (t, *J* = 7.1 Hz, 6H, N(CH$_2$CH_3)$_2$). ^{13}C NMR (101 MHz, $CDCl_3$): δ 192.6, 161.9, 157.5, 151.1, 144.0, 127.1, 117.4, 109.6, 103.8, 97.7, 44.9 (CH_2 × 2), 12.5 (CH_3 × 2). Data is in agreement with the literature.[3]

7-(Diethylamino)-4-(hydroxymethyl)-2*H*-chromen-2-one (S16)

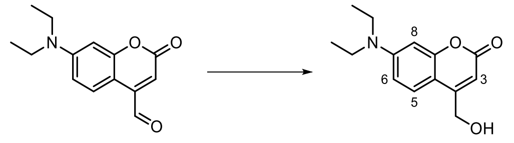

S15 **S16**

To a solution of 7-(diethylamino)-2-oxo-2*H*-chromene-4-carbaldehyde **S15** (3.53 g, 14.38 mmol) in THF (100 mL) was cooled to 0 °C was added $NaBH_4$ (1.09 g, 28.75 mmol). After stirring at 21 °C for 5 hours, the reaction mixture was diluted with $NaHCO_3$ (sat., 100 mL). The resulting aqueous phase was extracted with CH_2Cl_2 (3 × 50 mL). The combined organic layer was dried ($MgSO_4$), filtered and excess solvent removed to afford the title compound **S16** (2.66 g, 75%.) as a pale yellow solid. ^{1}H NMR (400 MHz, $CDCl_3$): δ 7.30 (d, *J* = 9.0 Hz, 1H, H-5), 6.54 (dd, *J* = 9.0, 2.6 Hz, 1H, H-6), 6.44 (d, *J* = 2.6 Hz, 1H, H-8), 6.27 (t, *J* = 1.3 Hz, 1H, H-3), 4.81 (s, 2H, CH_2OH), 3.37 (q, *J* = 7.1 Hz, 4H, N(CH_2CH$_3$)$_2$), 3.19 (s, 1H, CH$_2$O*H*), 1.17 (t, *J* = 7.1 Hz, 6H, N(CH$_2$CH_3)$_2$). ^{13}C NMR (101 MHz, $CDCl_3$): δ 162.7, 156.3, 154.6, 150.6, 124.5, 108.7, 106.4, 105.6, 97.9, 61.20, 44.9 (CH_2 × 2), 12.6 (CH_3 × 2). Data is in agreement with the literature.[3]

(7-(Diethylamino)-2-oxo-2*H*-chromen-4-yl)methyl (4-nitrophenyl)carbonate (2d)

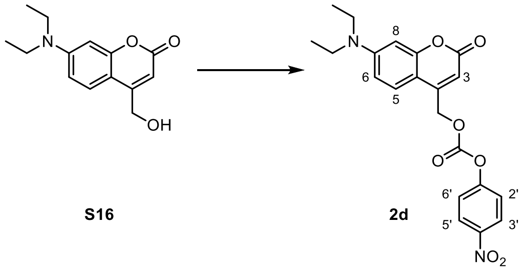

To a solution of 4-nitrophenylchloroformate (645 mg, 3.20 mmol) in CH_2Cl_2 (5 mL) cooled to 0 °C, was added pyridine (260 µL, 3.20 mmol). After stirring at 0 °C for 20 minutes, a solution of 7-(dimethylamino)-4-(hydroxymethyl)-2*H*-chromen-2-one **S16** (527 mg, 2.13 mmol) in CH_2Cl_2 (10 mL) was slowly added. The resulting reaction mixture was left to stir at 40 °C for 48 hours. Upon cooling to 21 °C, the organic layer was washed with HCl (2 M, 2 × 25 mL) and brine (10 mL). The resulting organic layer was then dried ($MgSO_4$), filtered and excess solvent removed. Purification by trituration in Et_2O at 21 °C for 15 minutes afforded to title compound **2d** (572 mg, 65%) as a pale yellow solid. ^{1}H NMR (400 MHz, $CDCl_3$): δ 8.30 (d, *J* = 9.2 Hz, 2H, H-3′, H-5′), 7.42 (d, *J* = 9.2 Hz, 2H, H-2′, H-6′), 7.31 (d, *J* = 9.0 Hz, 1H, H-5), 6.60 (dd, *J* = 9.0, 2.6 Hz, 1H, H-6), 6.53 (d, *J* = 2.6 Hz, 1H, H-8), 6.22 (t, *J* = 1.2 Hz, 1H, H-3), 5.40 (d, *J* = 1.2 Hz, 2H, CH_2), 3.42 (q, *J* = 7.1 Hz, 4H, N(CH_2CH$_3$)$_2$), 1.21 (t, *J* = 7.1 Hz, 6H, N(CH$_2$CH_3)$_2$). ^{13}C NMR (101 MHz, $CDCl_3$): δ 161.7, 156.5, 155.3, 152.3, 151.0, 147.8, 145.7, 125.5 (CH × 2), 124.4, 121.9 (CH × 2), 108.9, 107.0, 105.7, 98.0, 65.9, 44.9 (CH_2 × 2), 12.6 (CH_3 × 2). Data is in agreement with the literature.[4]

Carbamate 3d

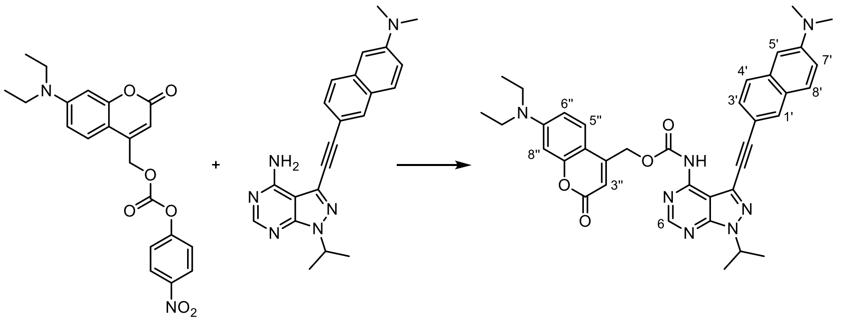

To a solution of compound **1** (103 mg, 0.278 mmol) in CH_2Cl_2 (anhydrous, 2 mL), was added (7-(diethylamino)-2-oxo-2*H*-chromen-4-yl)methyl (4-nitrophenyl)carbonate **2d** (138 mg, 0.334 mmol) and HOBt (45 mg, 0.334 mmol). The resulting reaction mixture was left to at 40 °C for 4 days. Upon cooling to 21 °C, excess solvent was removed. Purification by flash column chromatography (*Method B*) afforded the title compound **3d** (77 mg, 43%, R_f = 0.19 in 1:40 MeOH/$CHCl_3$) as a yellow solid. ^{1}H NMR (400 MHz, $CDCl_3$): δ 8.81 (s, 1H, H-6), 8.64 (s, 1H, NH), 7.94 (s, 1H, H-1′), 7.58 (d, *J* = 9.1 Hz, 1H, H-8′), 7.54 (d, *J* = 8.5 Hz, 1H, H-4′), 7.43 (dd, *J* = 8.5, 1.7 Hz, 1H, H-3′), 7.32 (d, *J* = 9.0 Hz, 1H, H-5′), 7.14 (dd, *J* = 9.1, 2.5 Hz, 1H, H-7′), 6.83 (br s, 1H, H-5′), 6.46 (d, *J* = 2.6 Hz, 1H, H-8″), 6.39

(dd, J = 9.0, 2.6 Hz, 1H, H-6″), 6.25 (s, 1H, H-3″), 5.40 (s, 2H, CH_2), 5.23 (sept, J = 6.7 Hz, 1H, C*H*$(CH_3)_2$), 3.33 (q, J = 7.1 Hz, 4H, N(C*H*$_2$$CH_3$)$_2$), 3.08 (s, 6H, $N(CH_3)_2$), 1.62 (d, J = 6.7 Hz, 6H,), 1.14 (t, J = 7.1 Hz, 6H, N(CH_2C*H*$_3$)$_2$). ^{13}C NMR (101 MHz, $CDCl_3$): δ 161.7, 156.5, 156.2, 152.8, 152.4, 150.8, 149.7, 149.6, 148.5, 135.4, 132.2, 129.1, 128.0, 126.7, 125.9, 125.6, 124.9, 116.8, 113.3, 108.9, 108.0, 106.1, 105.9, 104.0, 97.9 (CH × 1, C × 1), 79.7, 63.7, 50.1, 44.8 (CH_2 × 2), 40.7 (CH_3 × 2), 22.1 (CH_3 × 2), 12.5 (CH_3 × 2). HRMS (ESI/Q-TOF) *m/z*: [M + H]$^+$ Calcd for $C_{37}H_{38}N_7O_4$ 644.2980; Found: 644.2980.

Synthesis of compound 3e:

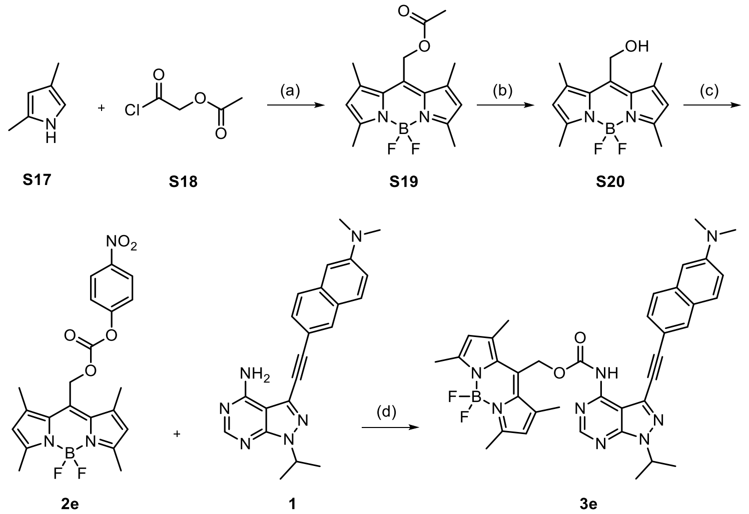

Reagents and Conditions**:** (a) *i*. CH_2Cl_2, 40 ° C, 2 h; *ii*. Et_3N, BF_3·OEt_2, 21 °C, 1 h; (b) NaOH (0.1 M), (1:1) MeOH/CH_2Cl_2, 21 °C, 2 h; (c) *p*-nitrophenol chloroformate, pyridine, CH_2Cl_2, 21 °C, 45 mins; (d) HOBt, CH_2Cl_2, 38 °C, 5 days.

8-Acetoxymethyl-1,3,5,7-tetramethyl pyrromethene fluoroborate (S19)

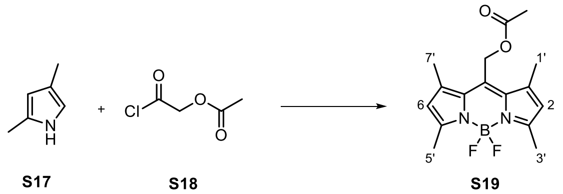

To a solution of 2,4-dimethylpyrrole **S17** (1.0 mL, 9.3 mmol) in CH_2Cl_2 (anhydrous, 40 mL) under nitrogen, was added 2-chloro-2-oxoethyl acetate **S18** (0.60 mL, 5.6 mmol). The resulting reaction mixture was left to stir at 40 °C for 2 hours. Next, the reaction mixture was cooled to 21 °C, to which Et_3N (3.20 mL, 27.9 mmol) was added. After stirring for 15 minutes BF_3·OEt_2 (5.20 mL, 42.0 mmol) was added dropwise. After stirring at 21 °C for 1 hour, the solvent was carefully removed under reduced pressure. Purification by flash column chromatography (50–100% CH_2Cl_2/pentane) afforded the title compound **S19** (595 mg, 40%, R_f = 0.63 in CH_2Cl_2) as a green-red solid. ^{1}H NMR (400 MHz, $CDCl_3$):

δ 6.08 (s, 2H, H-2, H-6), 5.29 (s, 2H, CH_2), 2.53 (s, 6H, H-3′, H-5′), 2.35 (s, 6H, H-1′, H-7′), 2.13 (s, 3H, $COCH_3$); ^{13}C NMR (101 MHz, $CDCl_3$): δ 170.7, 156.8 (C × 2), 141.6 (C × 2), 133.4, 132.8 (C × 2), 122.5 (CH × 2), 58.0, 20.7, 15.8 (CH_3 × 2), 14.8 (CH_3 × 2). Data is consistent with that reported in the literature.[5]

Carbonate 2e

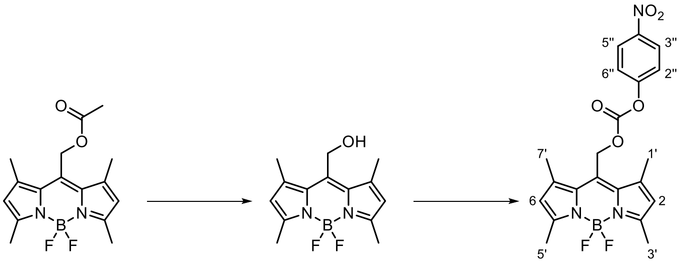

S19 S20 2e

To a solution of 8-acetoxymethyl-1,3,5,7-tetramethyl pyrromethene fluoroborate **S19** (422 mg, 1.32 mmol) in 2:1 MeOH/CH_2Cl_2 (25 mL) was added NaOH (0.1 M, 6.60 mL, 0.669 mmol). The resulting reaction mixture was purged with nitrogen and left to stir at 21 °C. After stirring for 2.5 hours, excess solvent was removed. The resulting residue was suspended in EtOAc (20 mL) and washed with NH_4OH (10 mL). The organic layer was dried over $MgSO_4$, filtered and dry loaded directly on silica. Purification by flash column chromatography (1:1 EtOAc/Pentane) afforded **S20** (254 mg, 69%) as a red solid. ^1H NMR (400 MHz, $CDCl_3$): δ 6.08 (s, 2H, H-2, H-6), 4.91 (d, *J* = 4.1 Hz, 2H, CH_2), 2.53 (s, 6H, CH_3 × 2), 2.51 (s, 6H, CH_3 × 2).

Next, a solution of 4-nitrophenyl chloroformate (543 mg, 2.70 mmol) in CH_2Cl_2 (10 mL) was cooled to 0 °C, to which pyridine (220 µL, 2.70 mmol) was added. After stirring at 0 °C for 10 minutes, the resulting cloudy solution was then slowly added to a solution of **S20** (250 mg, 0.899 mmol) in CH_2Cl_2 (10 mL). The resulting reaction mixture was allowed to stir at 21 °C for 45 minutes. An appropriate quantity of SiO_2 was then added to the reaction mixture and excess solvent was removed so that the crude material was dry loaded onto SiO_2. Purification by flash column chromatography (50–100% CH_2Cl_2/Pentane) afforded the title compound **2e** (273 mg, 69%, R_f = 0.61 in CH_2Cl_2) as a red solid. ^1H NMR (400 MHz, $CDCl_3$): δ 8.28 (d, *J* = 9.2 Hz, 2H, H-3″, H-5″), 7.39 (d, *J* = 9.2 Hz, 2H, H-2″, H-6″), 6.12 (s, 2H, H-2, H-6), 5.56 (s, 2H, CH_2), 2.54 (s, 6H, H-3′, H-5′), 2.46 (s, 6H, H-1′, H-7′). ^{13}C NMR (101 MHz, $CDCl_3$): δ 157.4 (C × 2), 155.4, 152.3, 145.7, 141.6 (C × 2), 132.7 (C × 2), 131.1, 125.5 (CH × 2), 122.8 (CH × 2), 121.8 (CH × 2), 61.8, 15.9 (CH_3 × 2), 14.9 (CH_3 × 2). Data is consistent with that reported in the literature.[5b]

Carbamate 3e

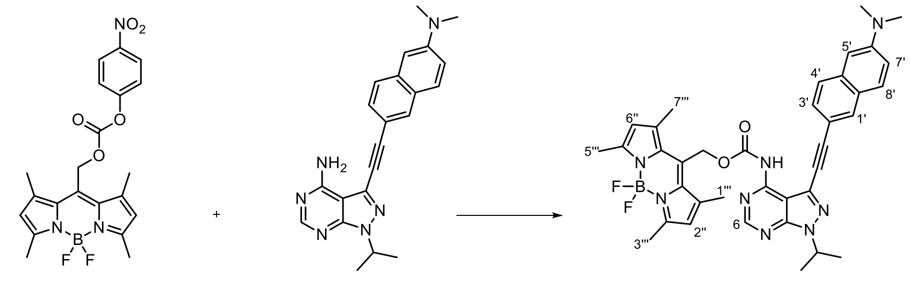

2e **1** **3e**

To a solution of compound **1** (85 mg, 0.229 mmol) in CH_2Cl_2 (3 mL) was added carbonate **2e** (122 mg, 0.275 mmol) and HOBt (37 mg, 0.275 mmol). The resulting reaction mixture was left to stir at 38 °C for 5 days. Then reaction mixture was then cooled to 21 °C and excess solvent was removed. Purification by flash column chromatography (*Method D*) afforded the title compound **3e** (69 mg, 45%) as a red solid. ^{1}H NMR (400 MHz, $CDCl_3$): δ 8.83 (s, 1H, H-6), 8.76 (s, 1H, NH), 7.81 (d, *J* = 1.3 Hz, 1H, H-1′), 7.43 (d, *J* = 9.1 Hz, 1H, H-8′), 7.28–7.25 (m, 1H, H-3′), 7.20 (d, *J* = 8.6 Hz, 1H, H-4′), 7.15 (dd, *J* = 9.1, 2.6 Hz, 1H, H-7′), 6.85 (d, *J* = 2.6 Hz, 1H, H-5′), 5.96 (s, 2H, H-2″, H-6″), 5.55 (s, 2H, CH_2), 5.23 (sept, *J* = 6.7 Hz, 1H, C*H*(CH_3)$_2$), 3.10 (s, 6H, N(CH_3)$_2$), 2.56 (s, 6H, H-3‴, H-5‴), 2.38 (s, 6H, H-1‴, H-7‴), 1.62 (d, *J* = 6.7 Hz, 6H, CH(C*H*$_3$)$_2$). ^{13}C NMR (101 MHz, $CDCl_3$): δ 157.1, 156.3, 152.8, 152.4, 149.7, 149.6, 141.8, 135.5, 133.0, 131.9, 131.7, 129.0, 127.8, 126.7, 125.7, 125.7, 122.8 (CH × 2), 116.8, 112.9, 105.9, 104.0, 98.3, 79.7, 59.6, 50.1, 40.6 (CH_3 × 2), 22.2 (CH_3 × 2), 15.8 (CH_3 × 2), 15.0 (CH_3 × 2). HRMS (ESI/Q-TOF) *m/z*: $[M + H]^+$ Calcd for $C_{37}H_{38}BF_2N_8O_2$ 674.3210; Found: 674.3240.

^{1}H and ^{13}C NMR Spectra of Compounds 4a, 4b and 3c–e

^{1}H NMR spectrum of compound **S2** (400 MHz, $CDCl_3$)

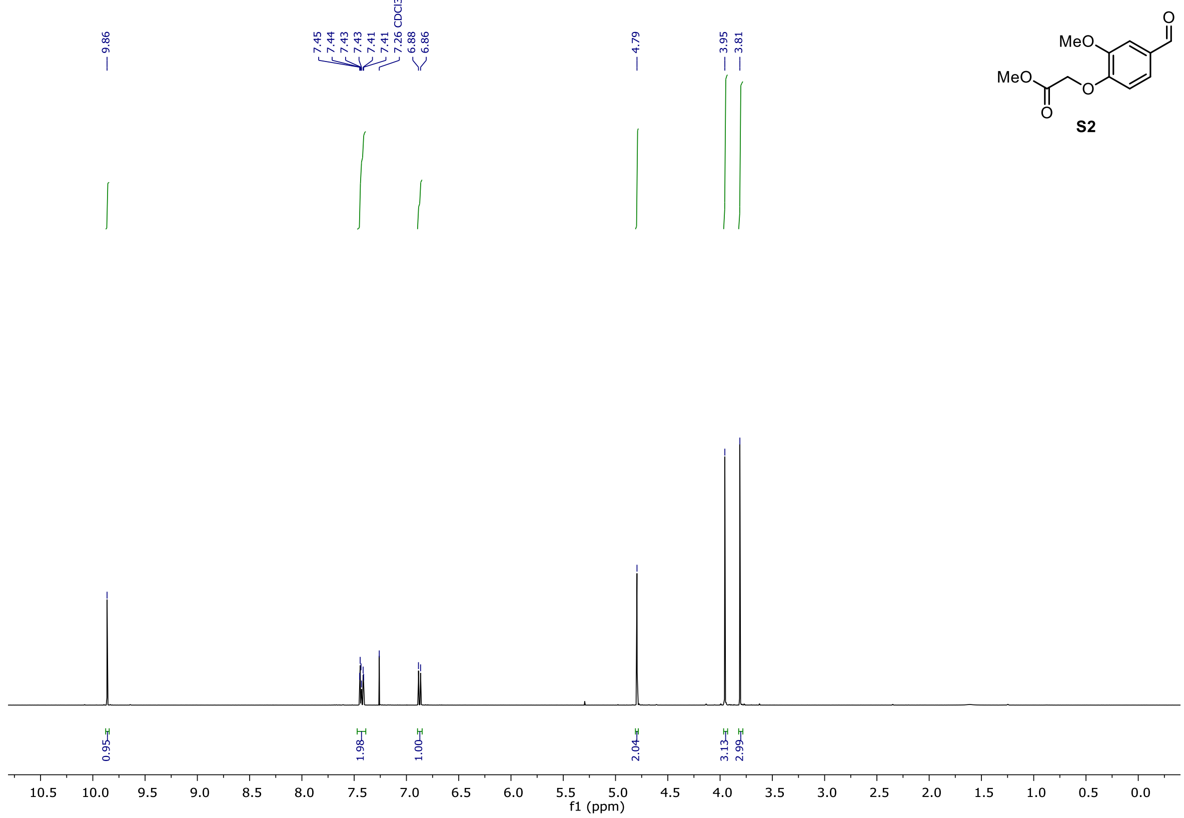

^{1}H NMR spectrum of compound **S3** (400 MHz, $CDCl_3$)

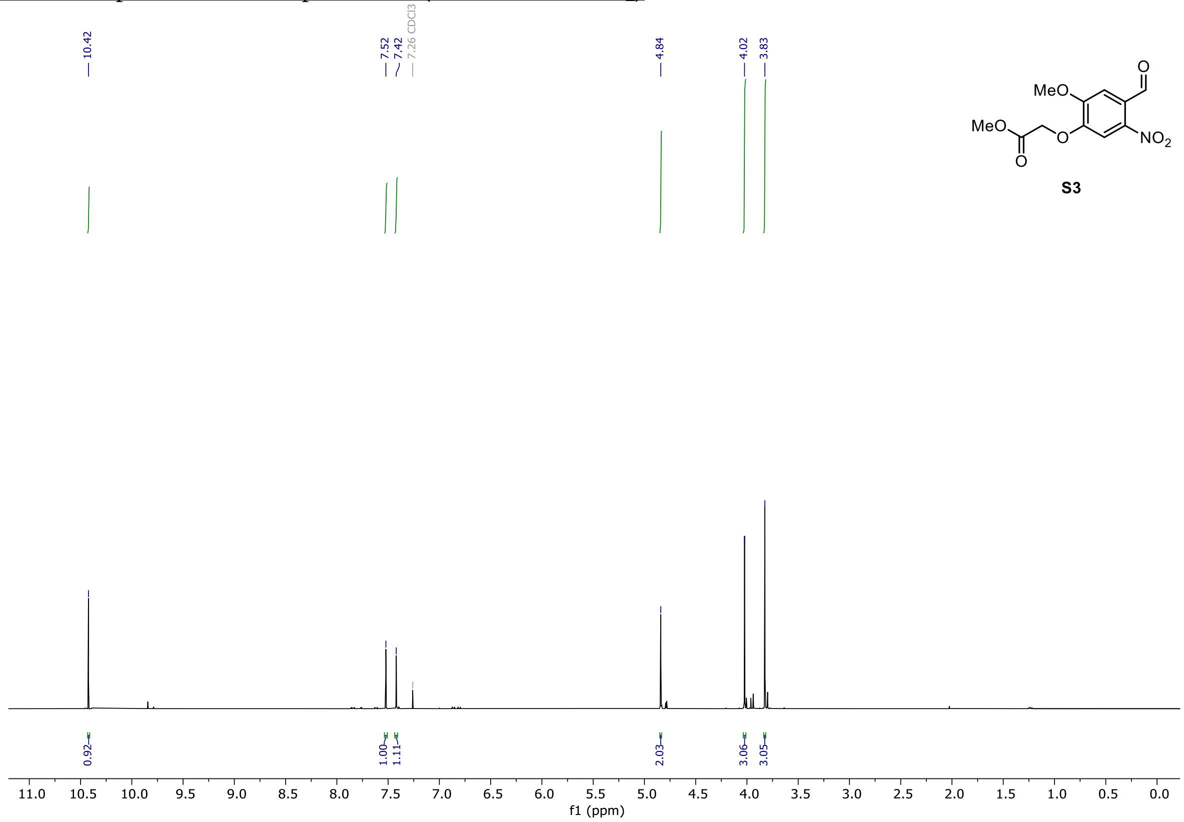

^{13}C NMR spectrum of compound **S3** (101 MHz, $CDCl_3$)

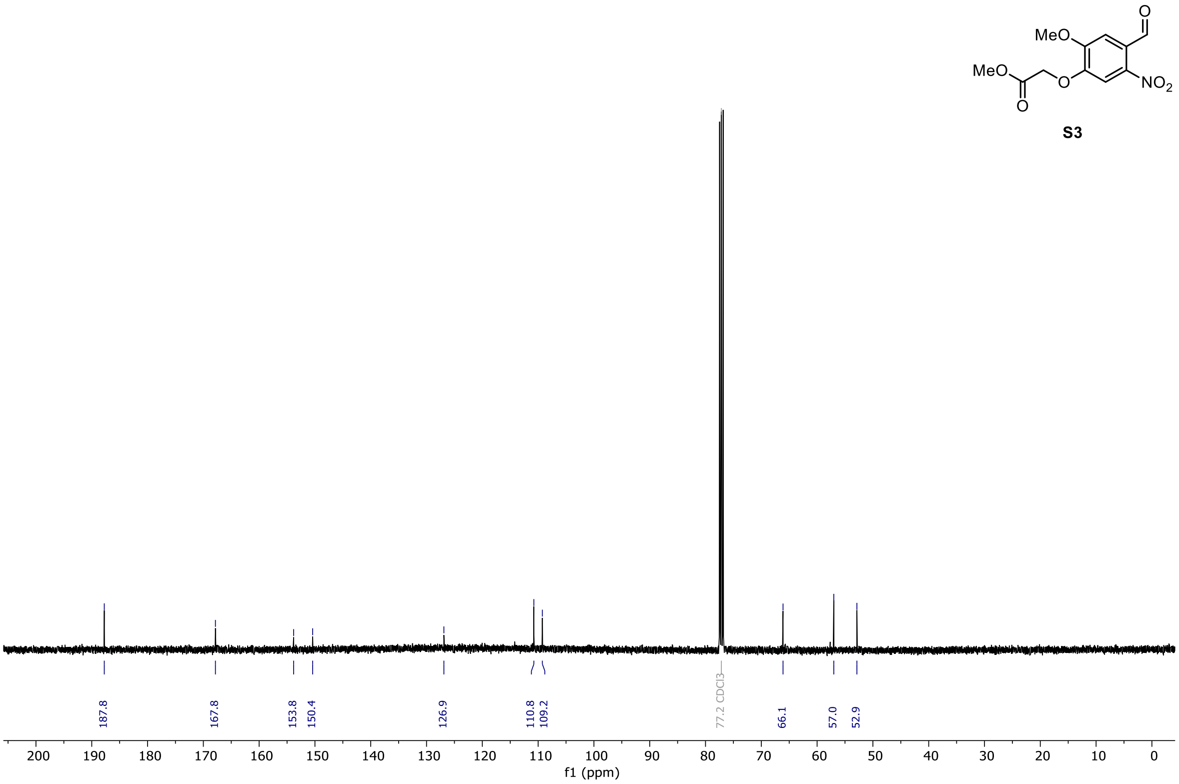

HMBC of compound **S3** (400 MHz, $CDCl_3$)

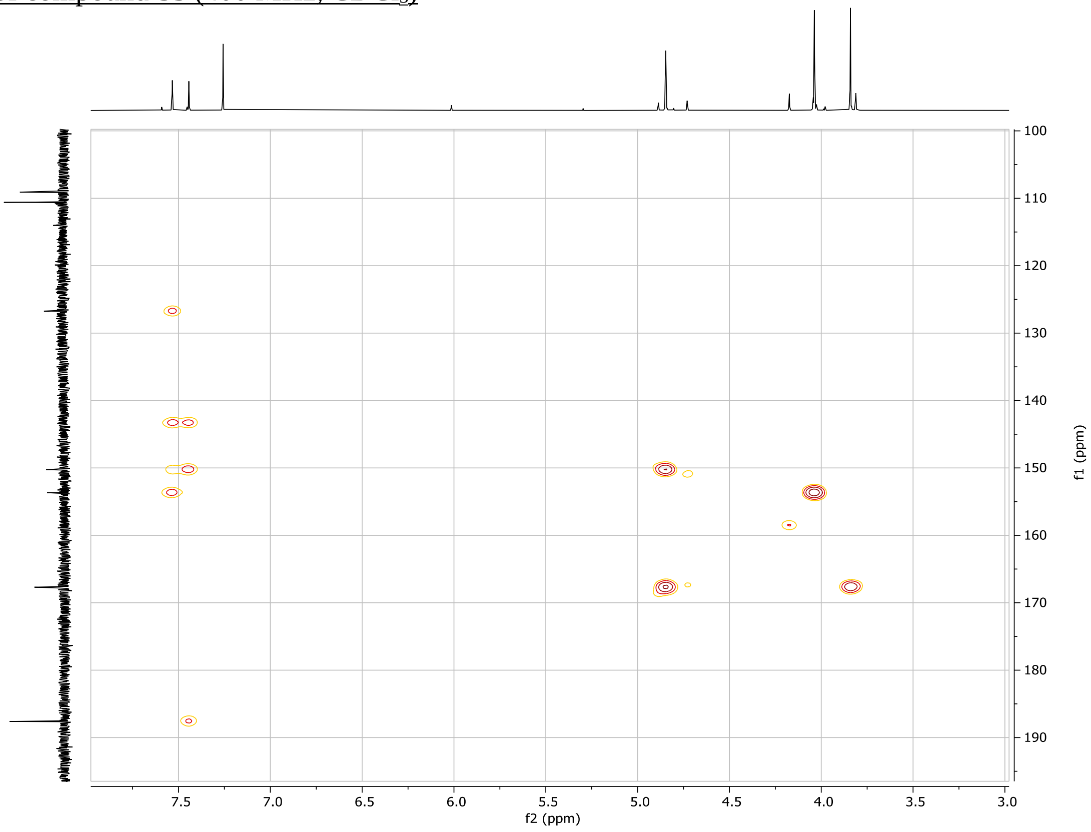

^{1}H NMR spectrum of compound **S4** (400 MHz, $CDCl_3$)

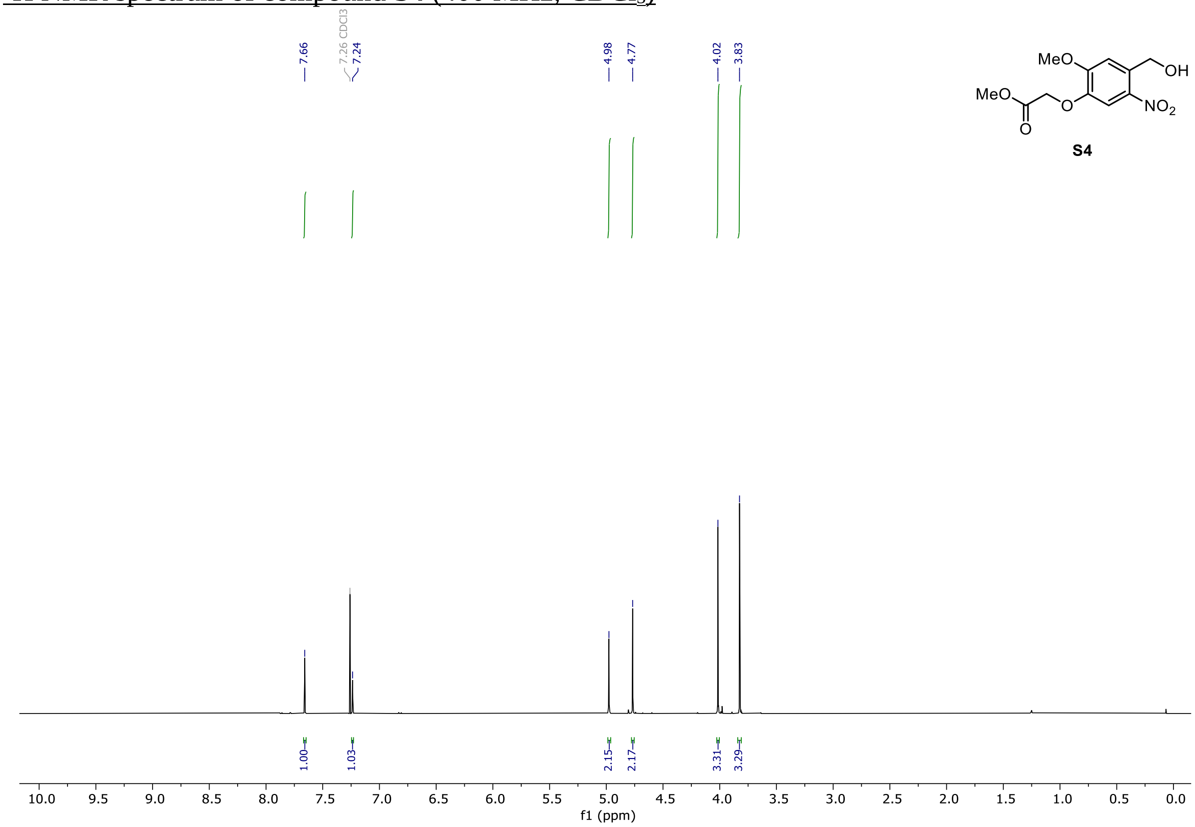

^{13}C NMR spectrum of compound **S4** (101 MHz, $CDCl_3$)

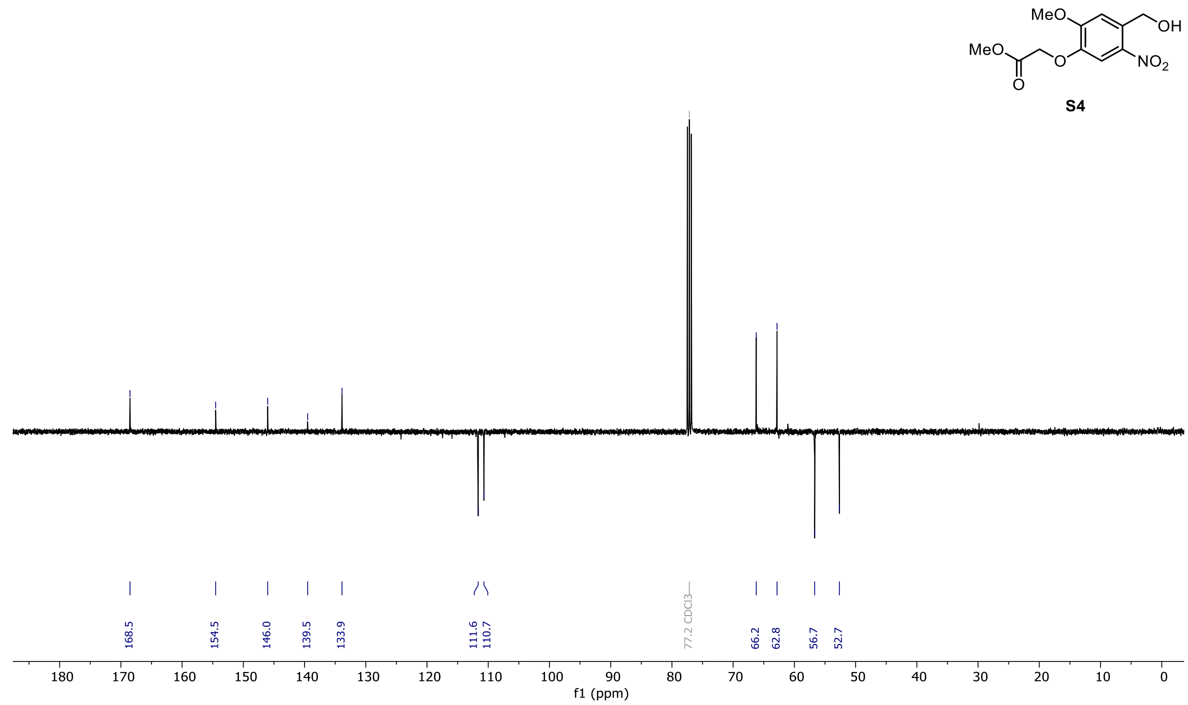

^{1}H NMR spectrum of compound **2a** (400 MHz, $CDCl_3$)

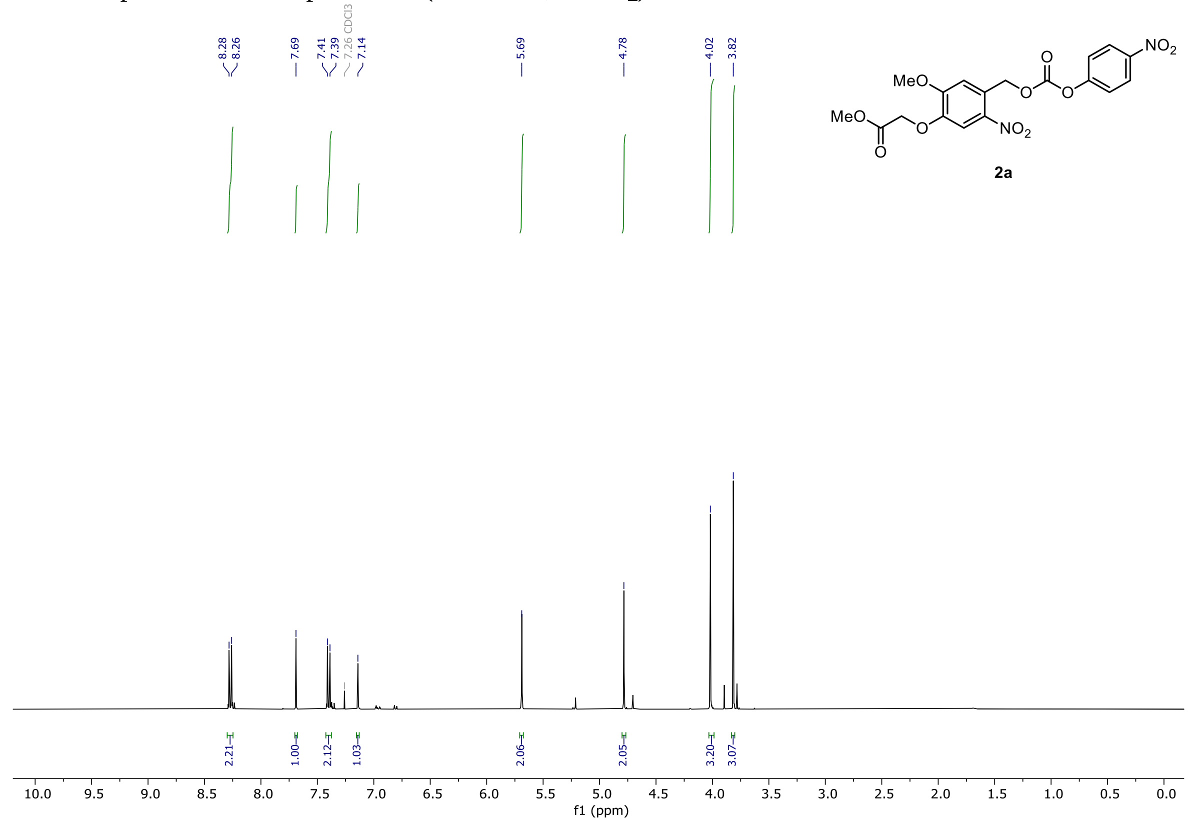

^{13}C NMR spectrum of compound **2a** (101 MHz, $CDCl_3$)

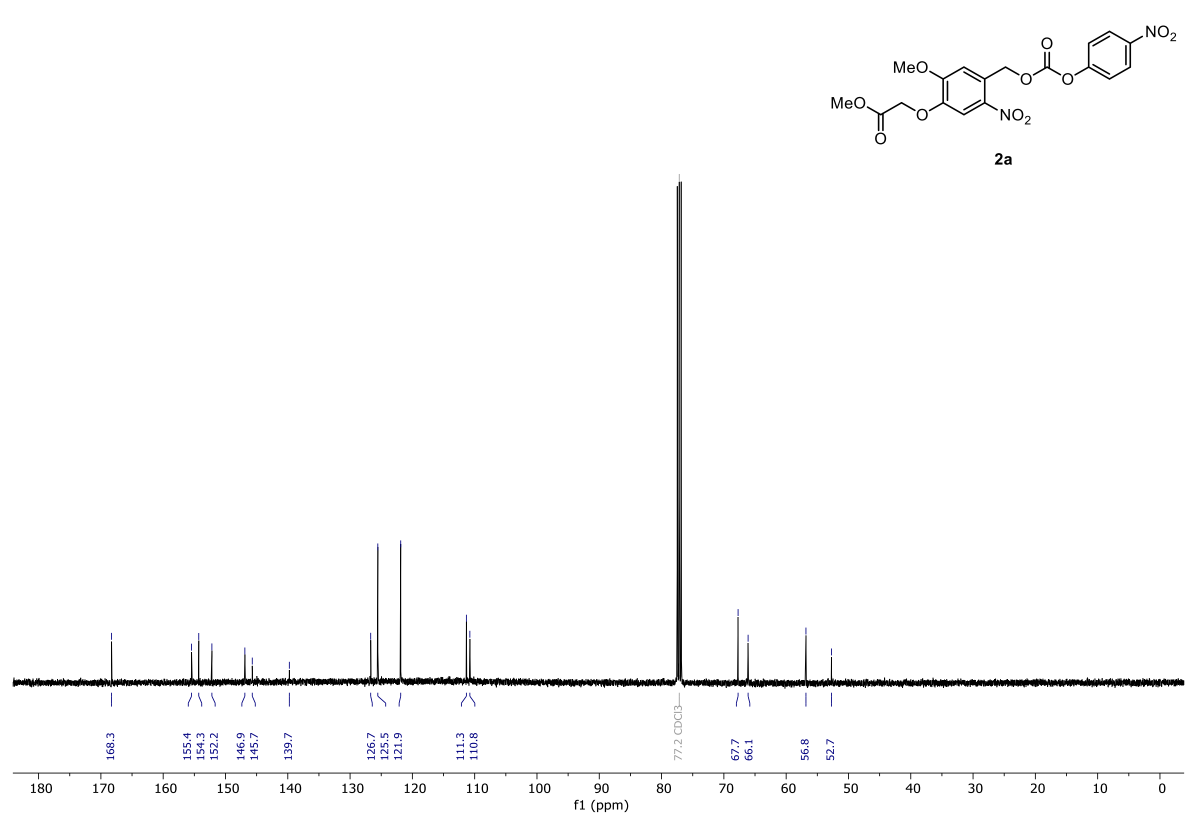

^{1}H NMR spectrum of **3a** (400 MHz, $CDCl_3$)

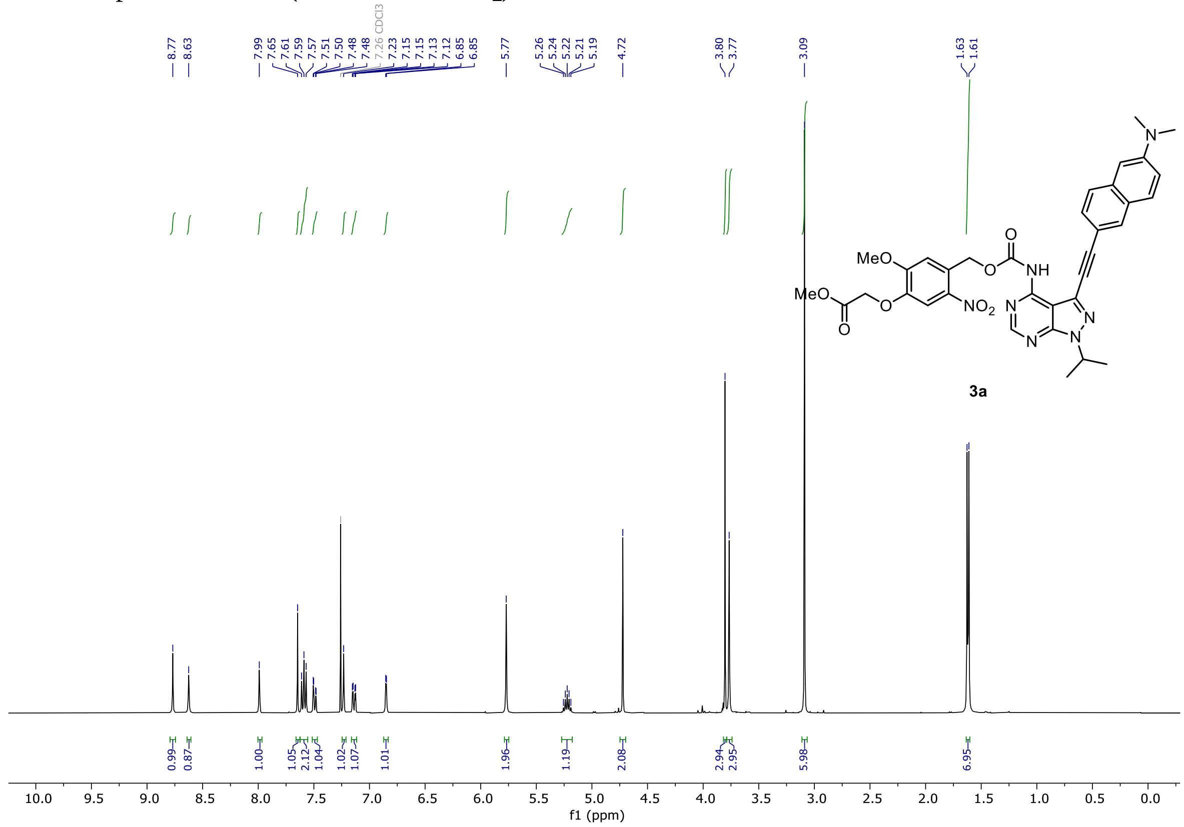

^{13}C NMR spectrum of **3a** (101 MHz, $CDCl_3$)

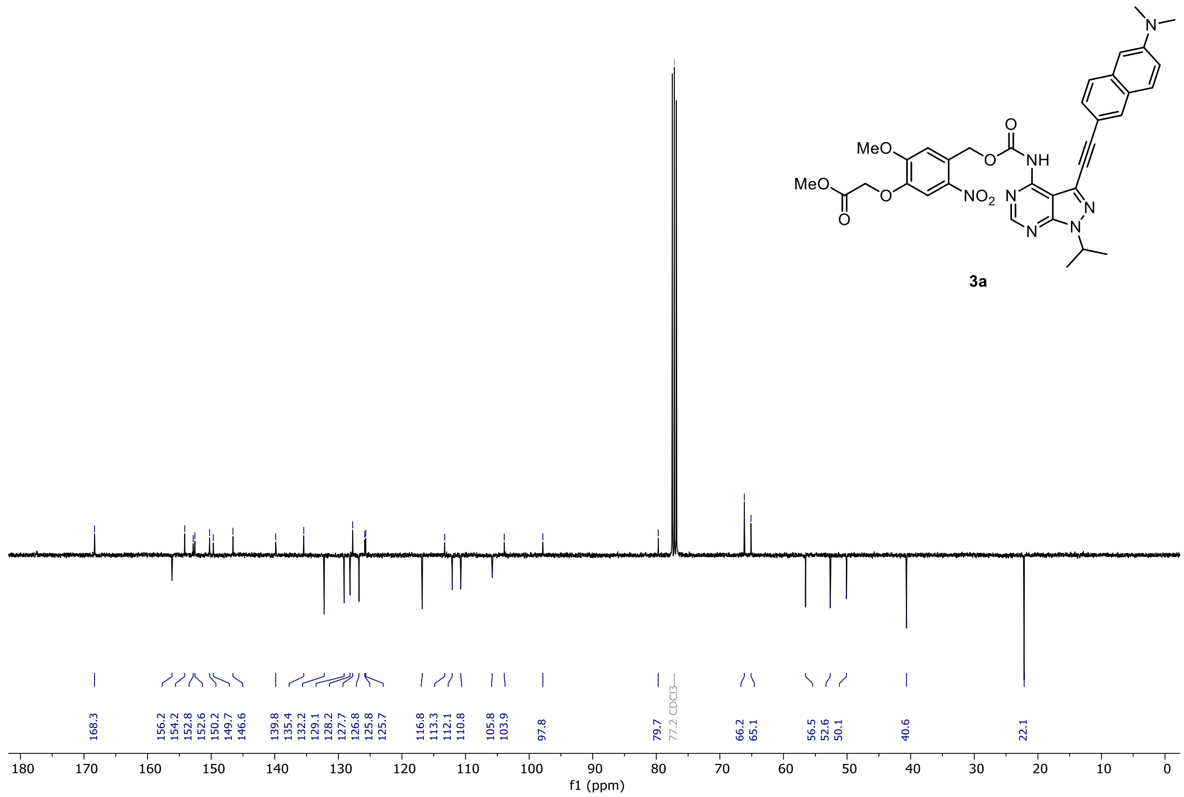

HSQC spectrum of **3a** ($CDCl_3$)

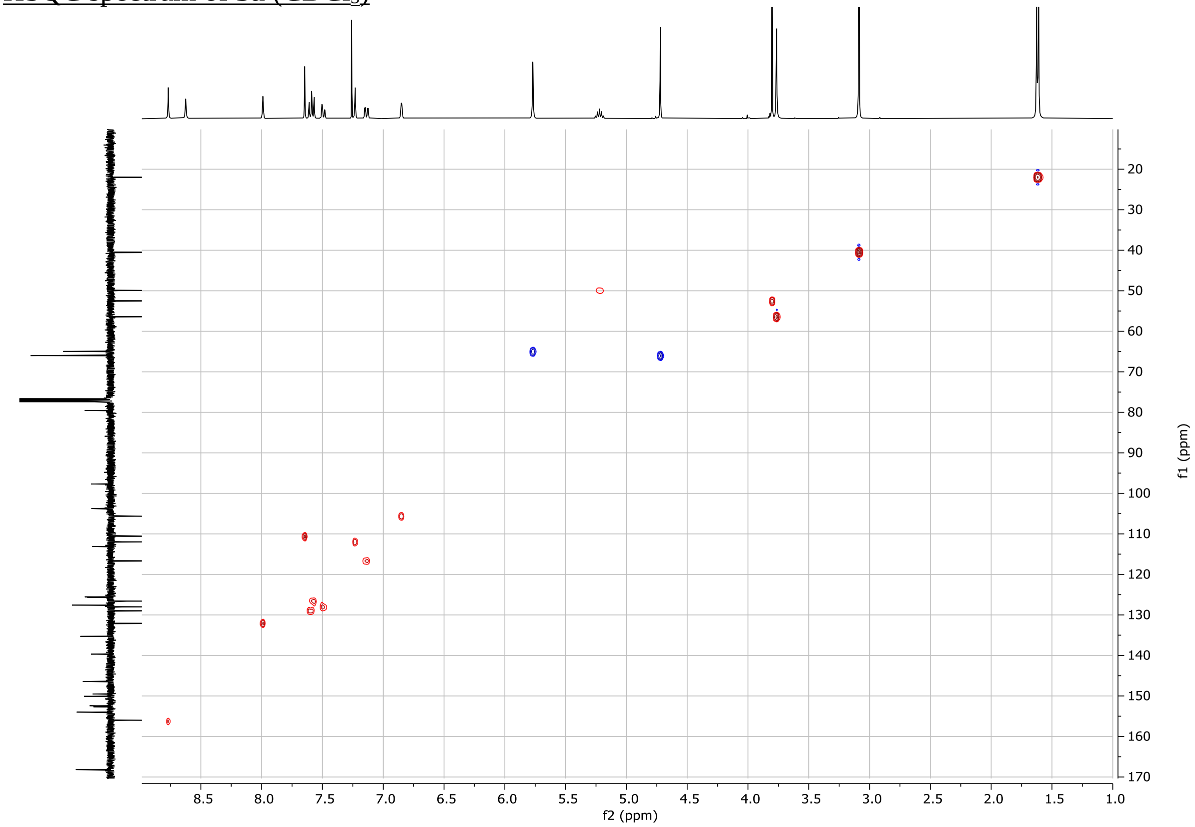

HMBC spectrum of **3a** ($CDCl_3$)

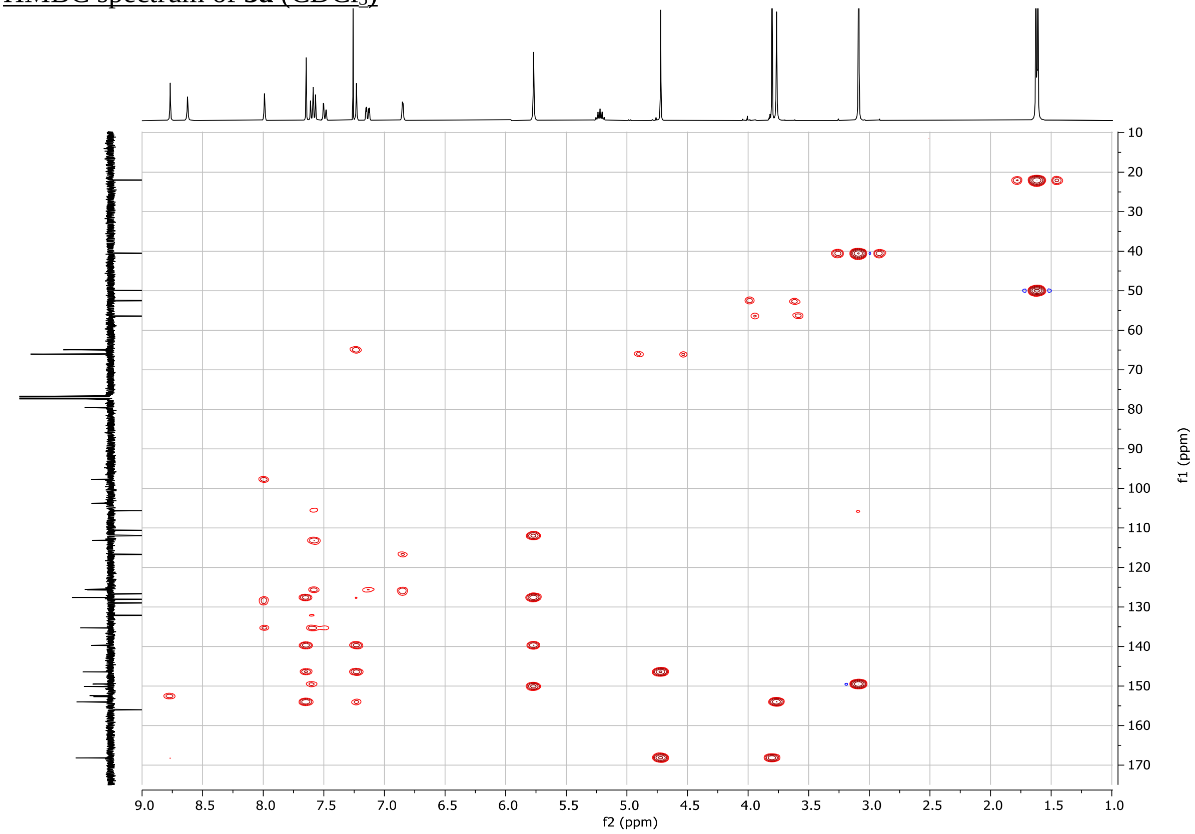

^{1}H NMR spectrum of **4a** (400 MHz, DMSO-d_6)

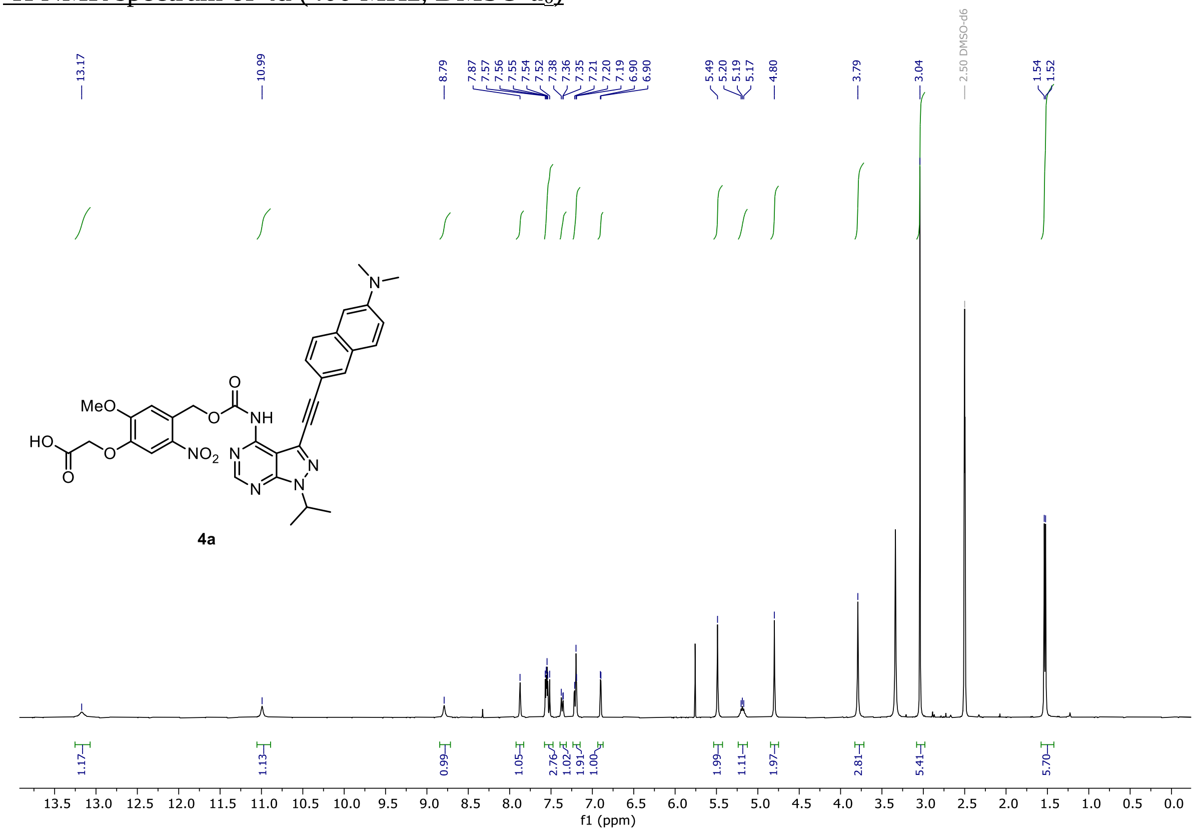

^{13}C NMR spectrum of **4a** (101 MHz, DMSO-d_6)

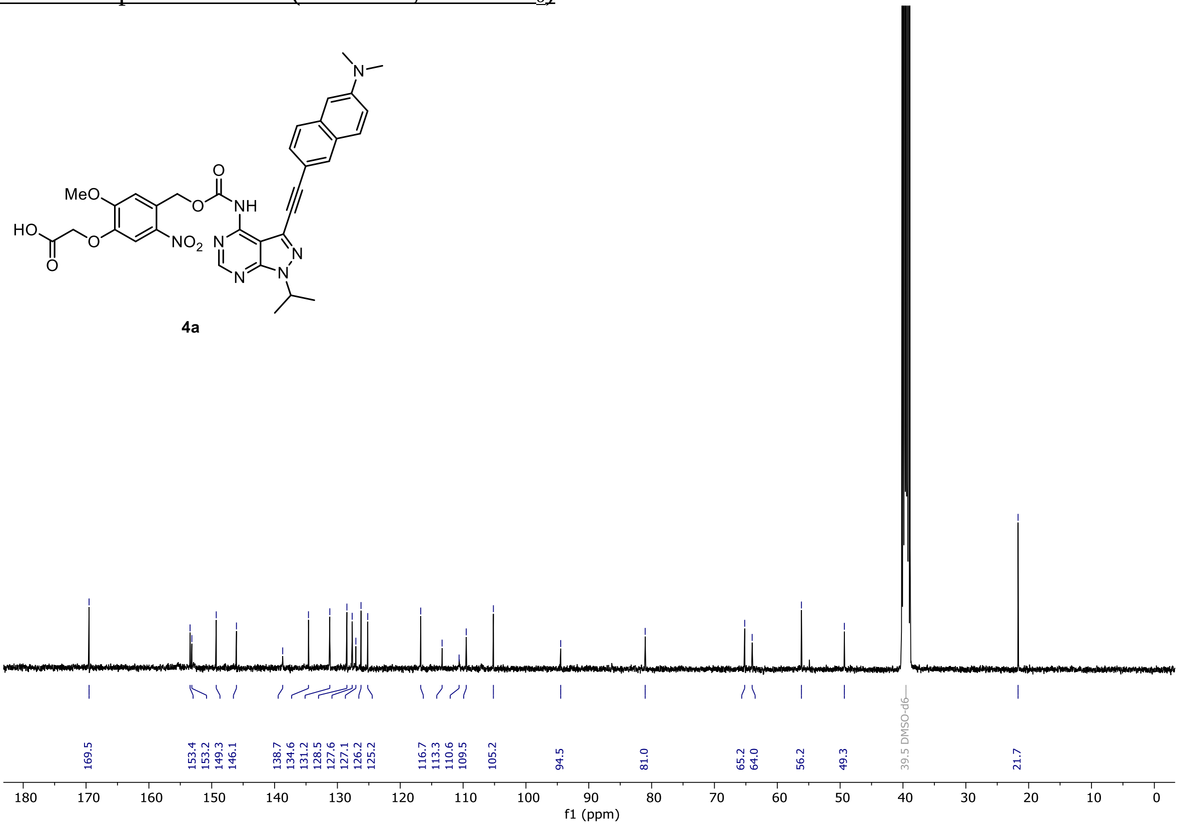

HSQC spectrum of **4a** (DMSO-d_6)

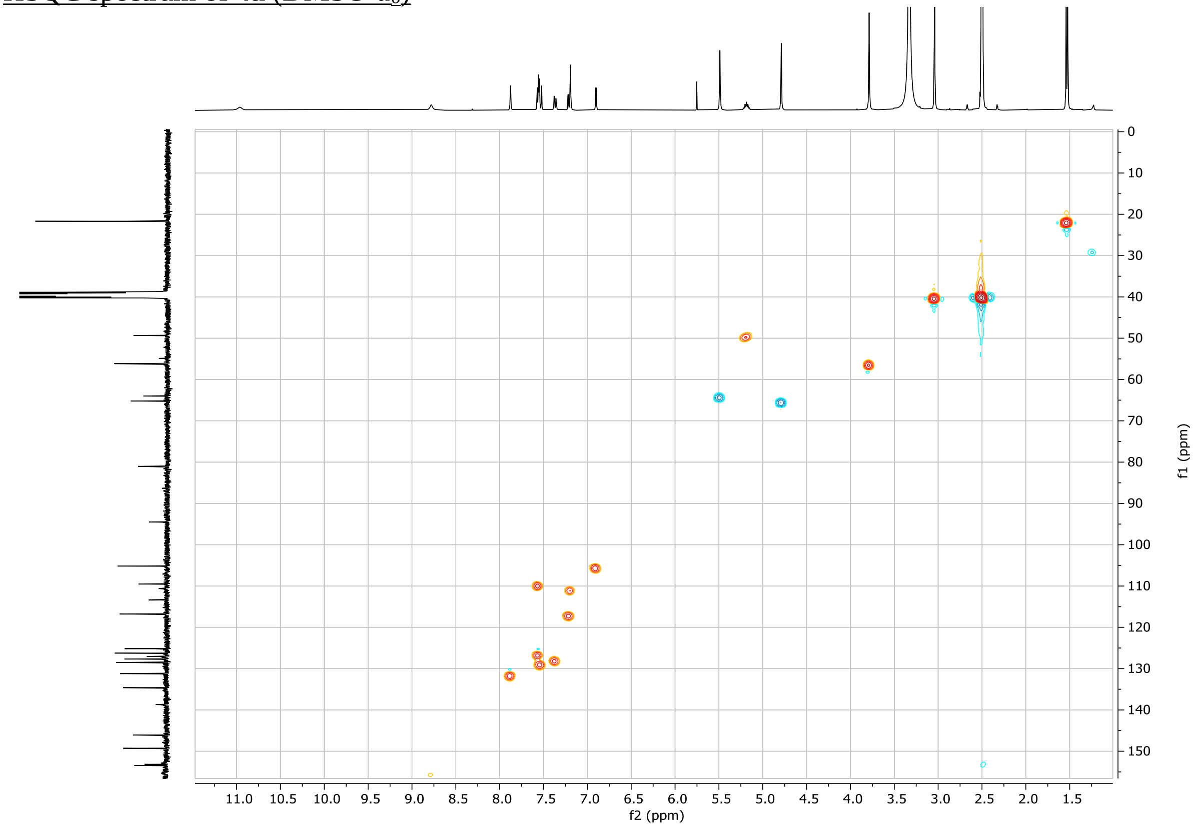

HMBC spectrum of **4a** (DMSO-d_6)

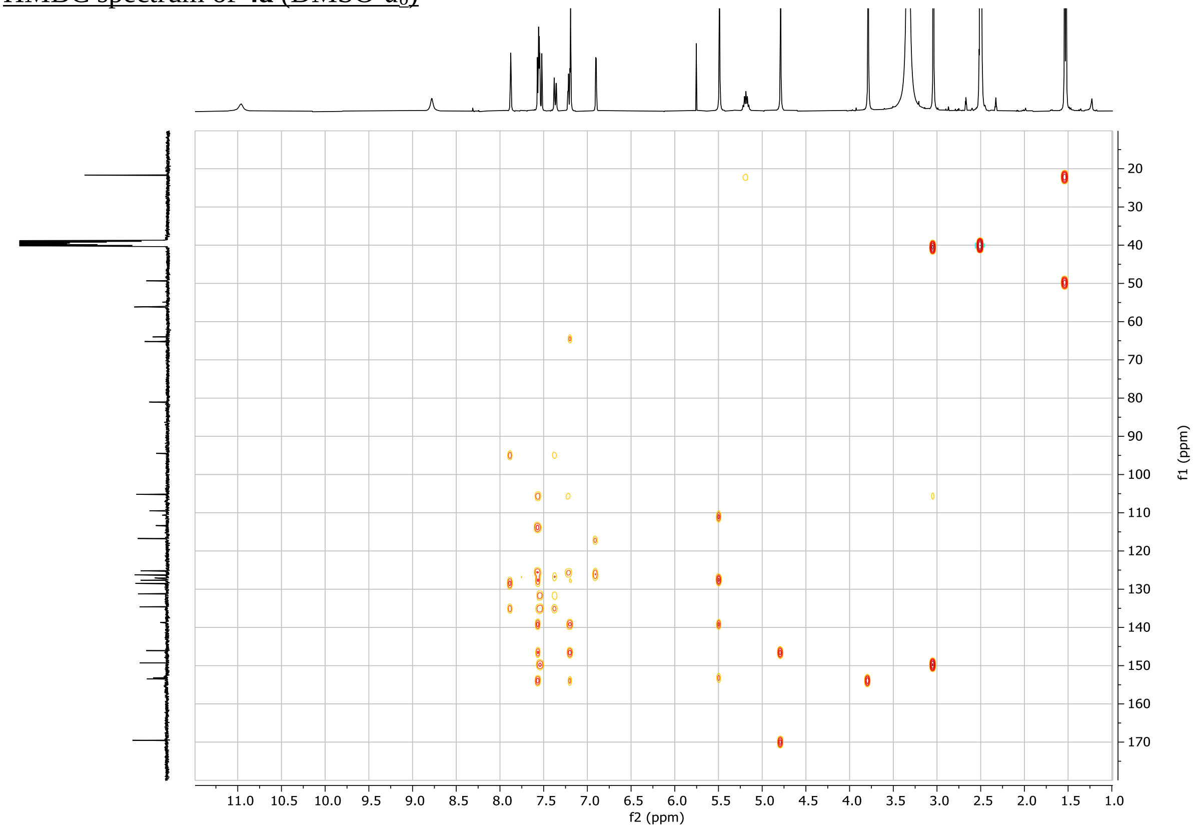

^{1}H NMR spectrum of compound **S6** (400 MHz, $CDCl_3$)

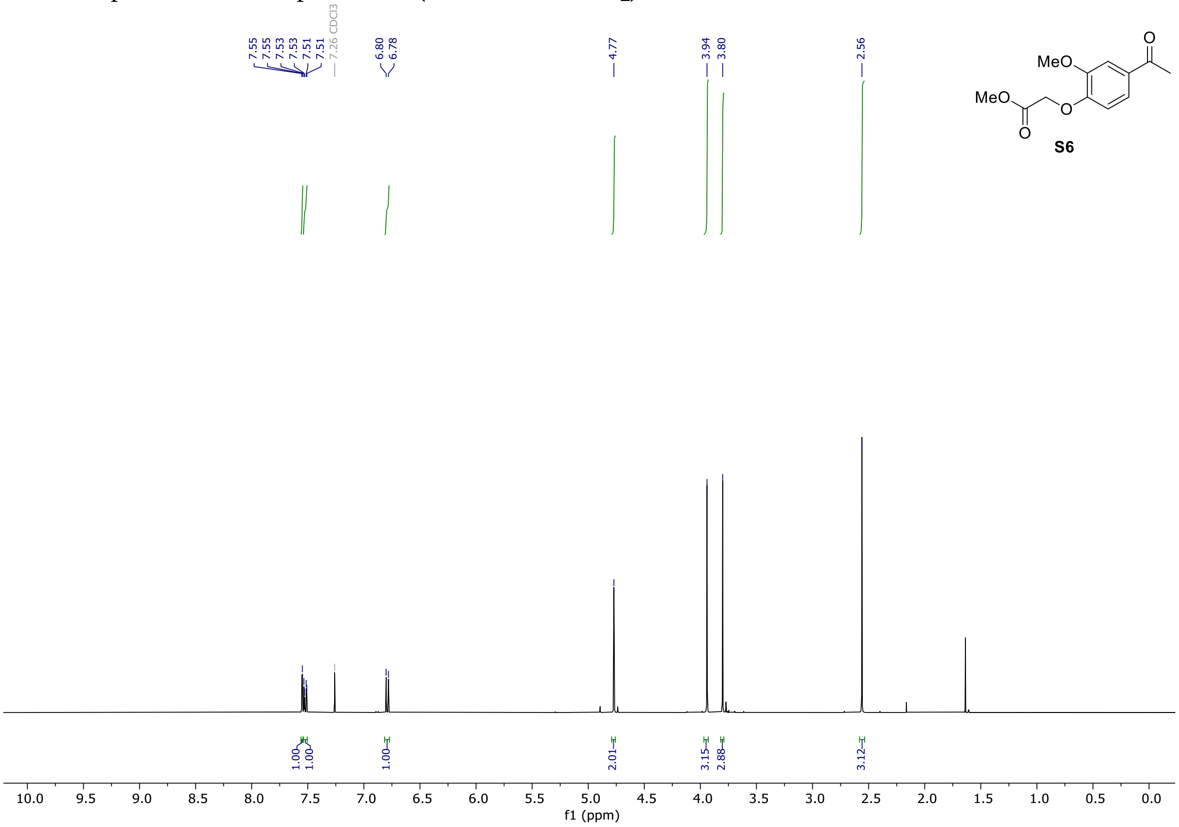

^{13}C NMR spectrum of compound **S6** (101 MHz, $CDCl_3$)

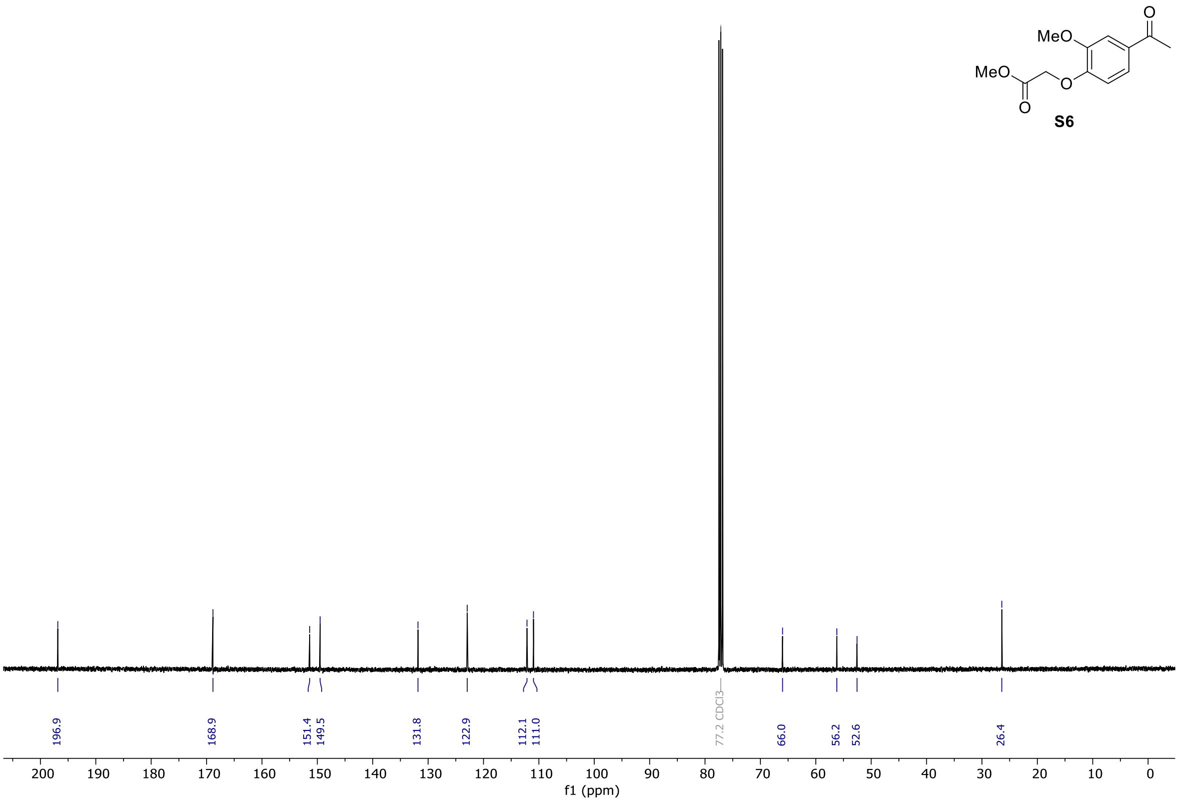

^{1}H NMR spectrum of compound **S7** (400 MHz, $CDCl_3$)

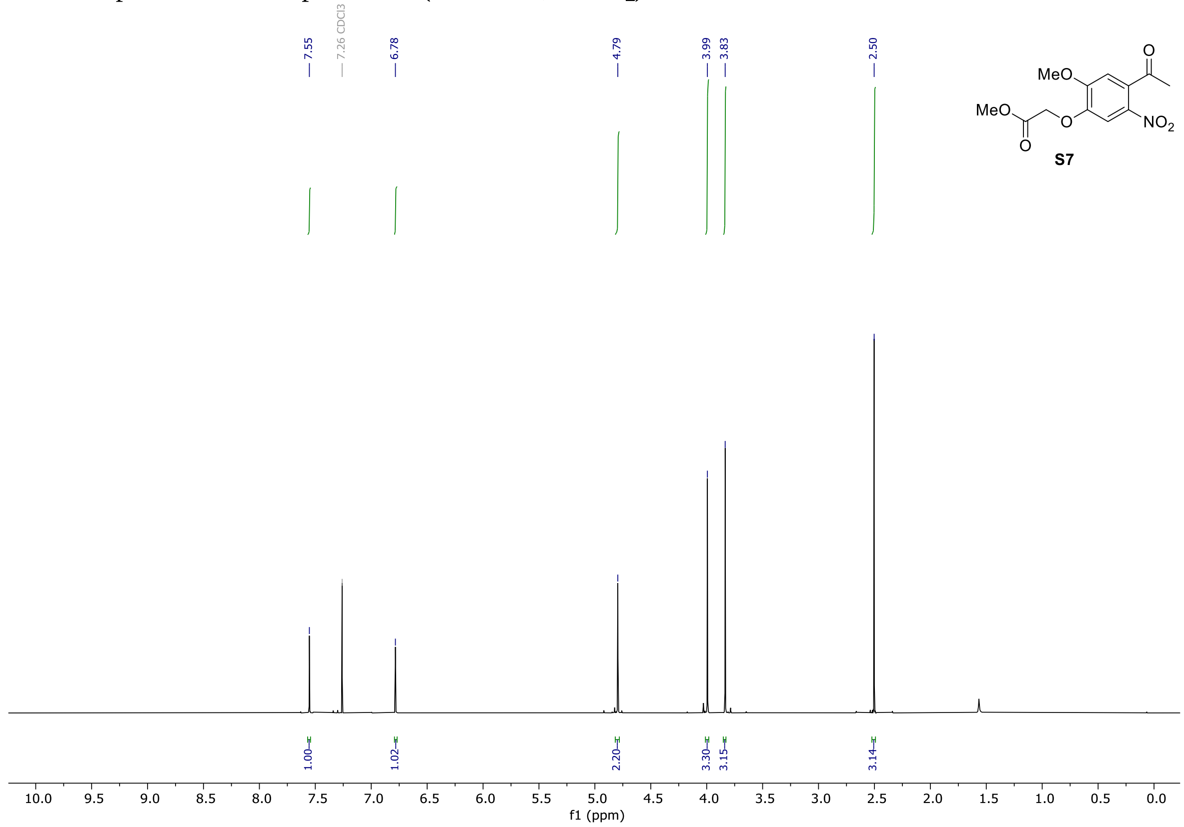

^{13}C NMR spectrum of compound **S7** (101 MHz, $CDCl_3$)

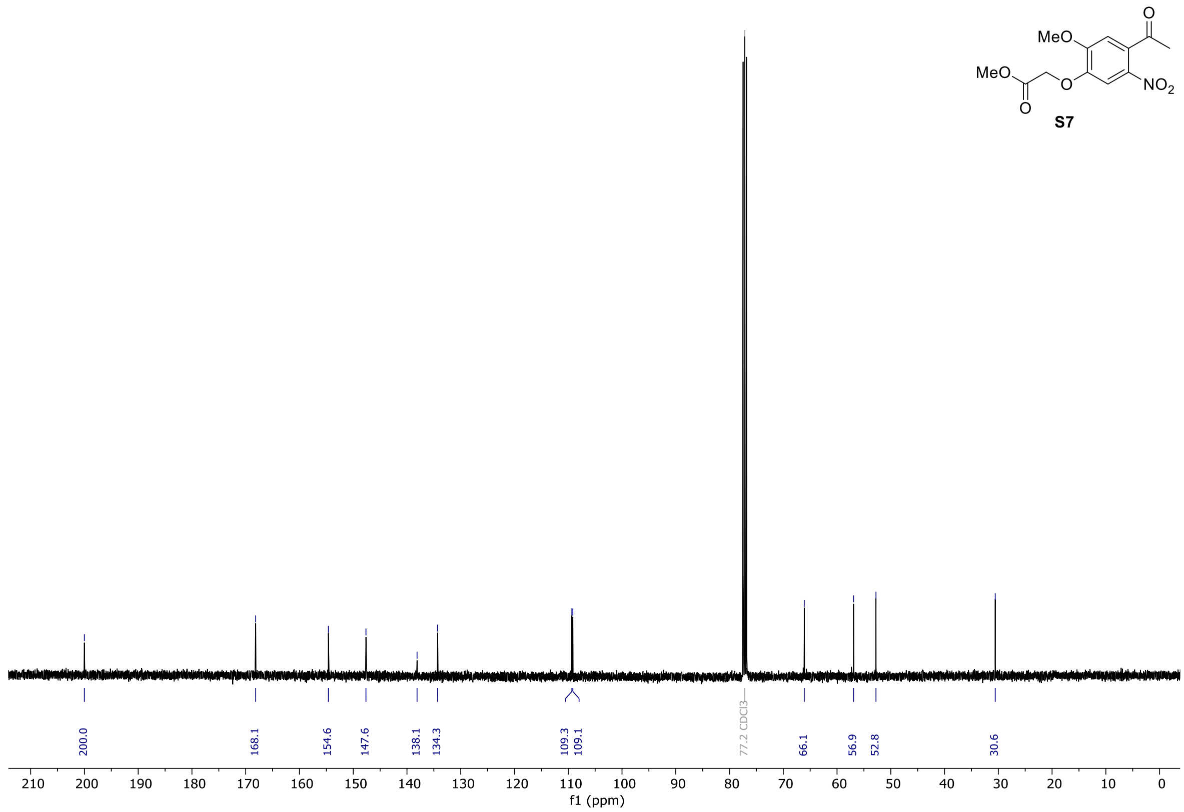

^{1}H NMR spectrum of compound **S8** (400 MHz, $CDCl_3$)

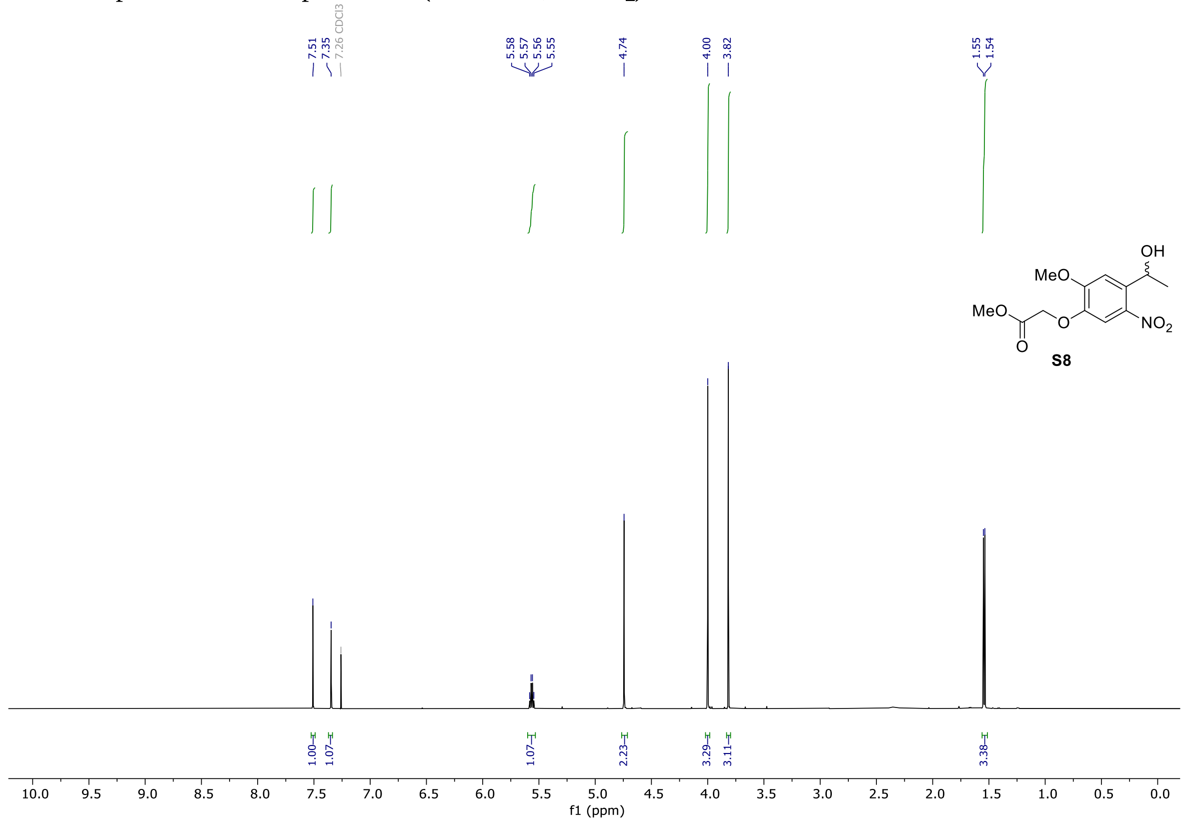

^{13}C NMR spectrum of compound **S8** (101 MHz, $CDCl_3$)

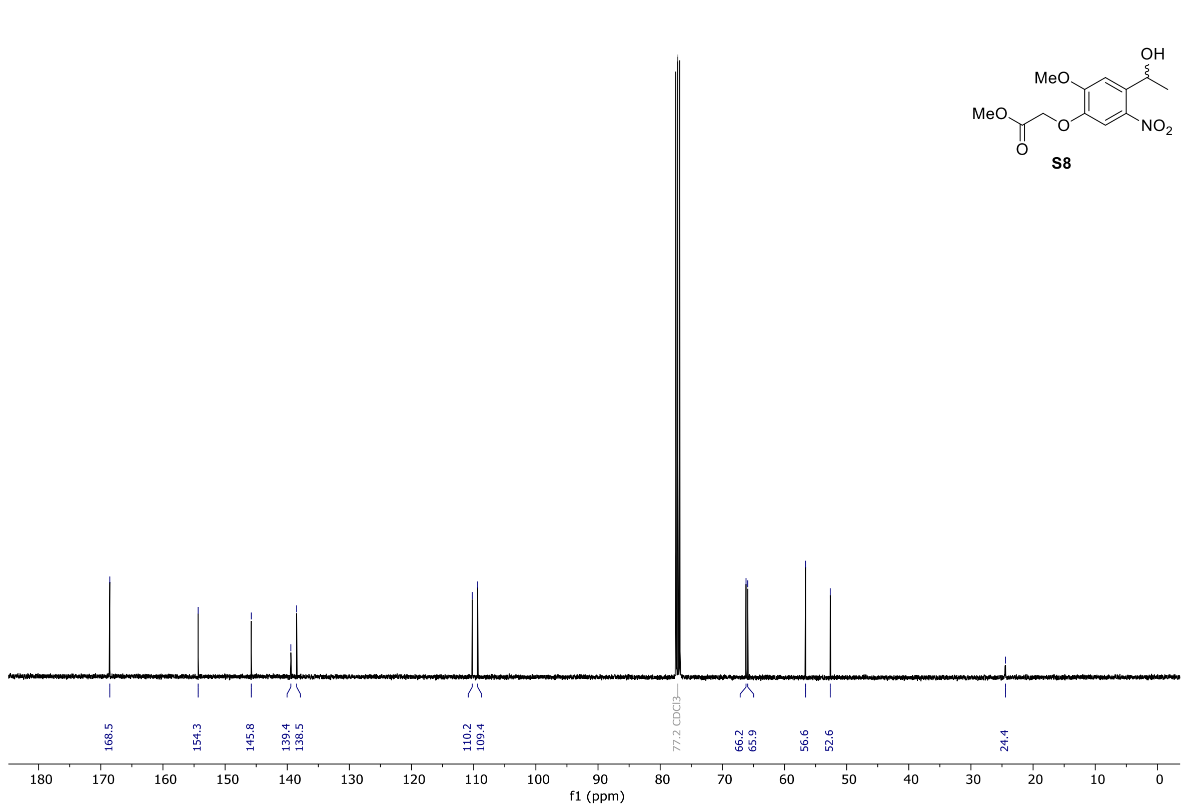

^{1}H NMR spectrum of compound **2b** (400 MHz, $CDCl_3$)

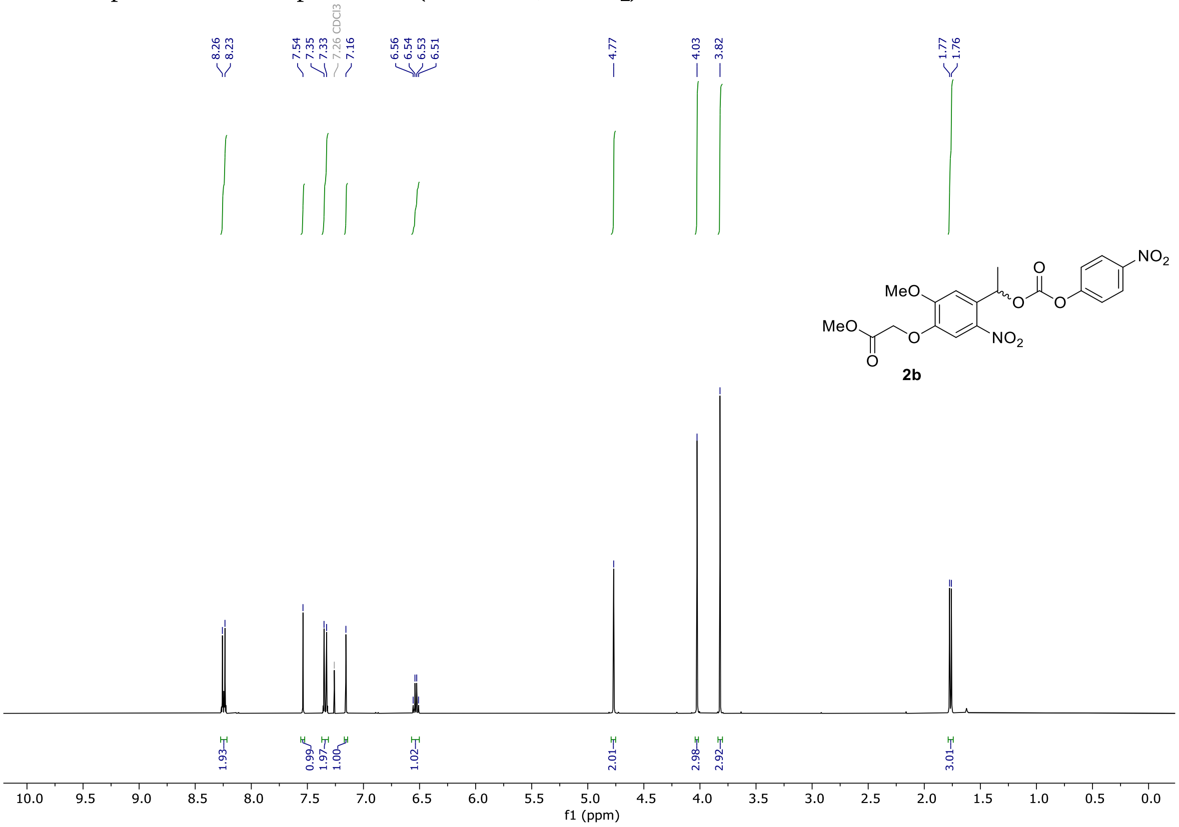

^{13}C NMR spectrum of compound **2b** (101 MHz, $CDCl_3$)

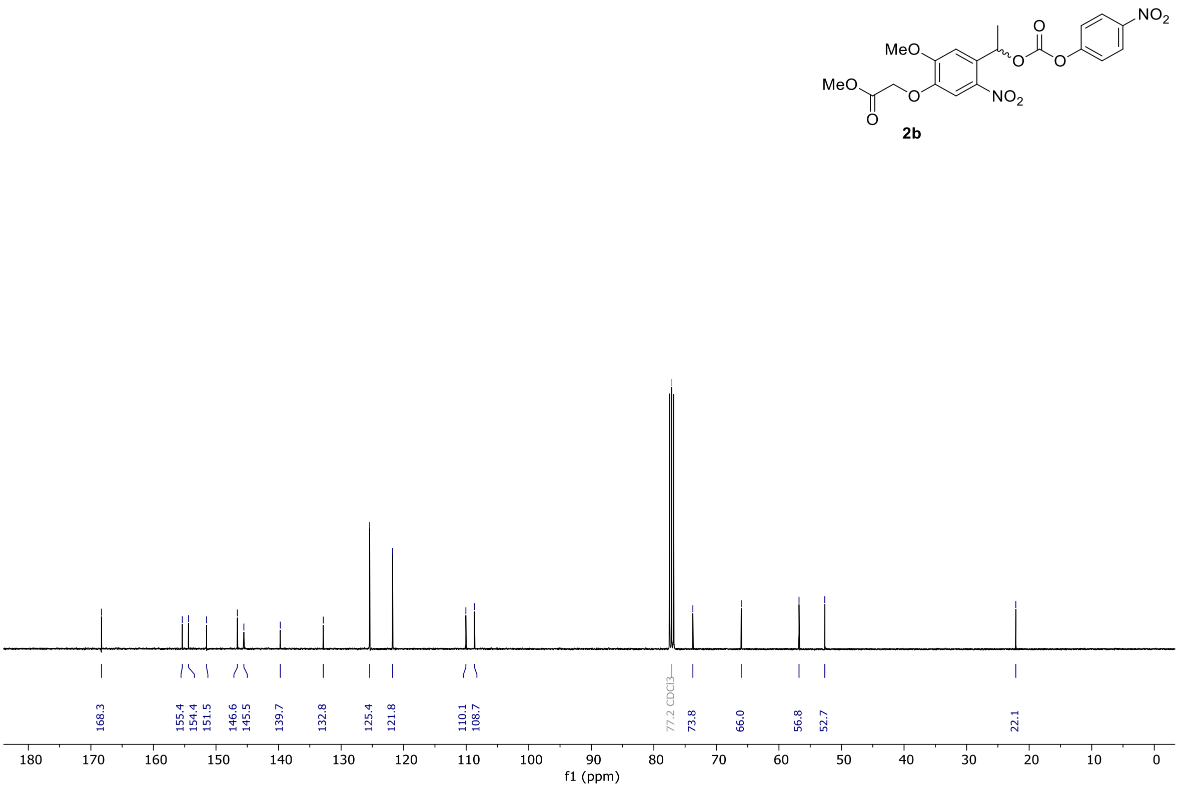

^{1}H NMR spectrum of **3b** (500 MHz, $CDCl_3$)

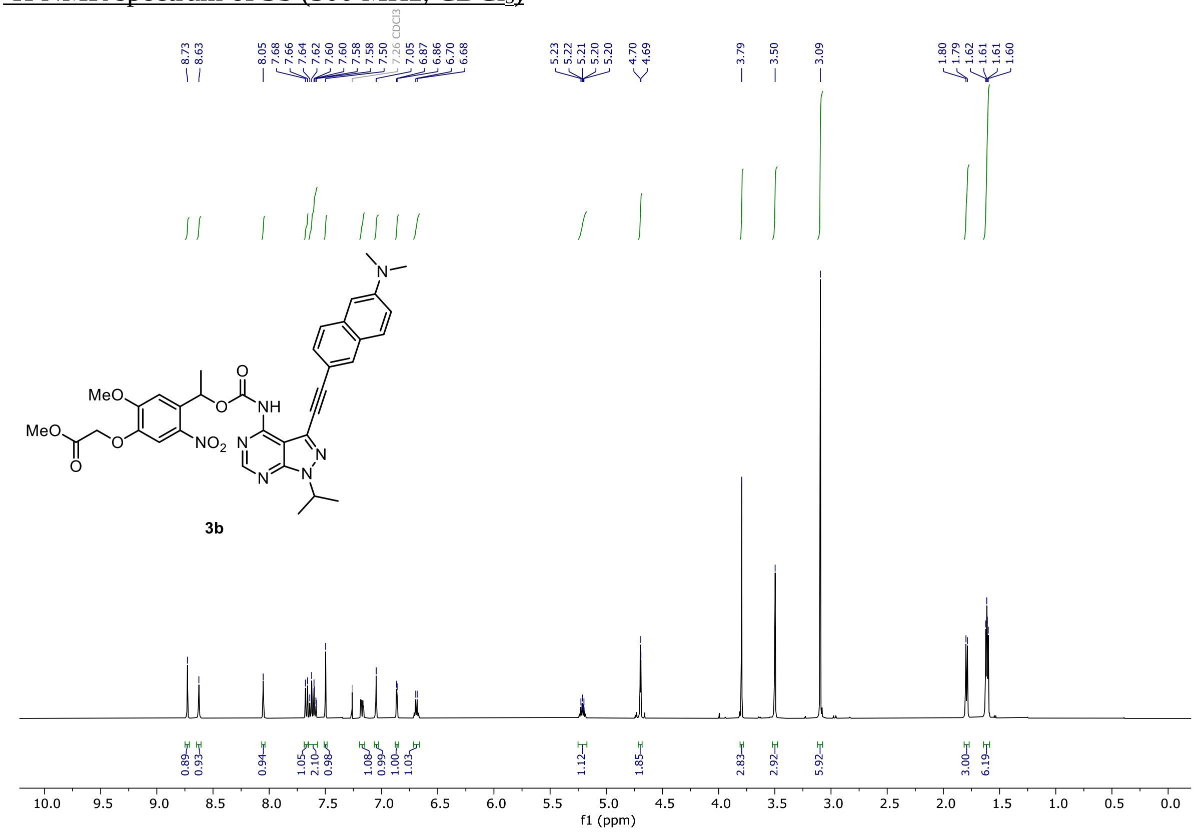

^{13}C NMR spectrum of **3b** (126 MHz, $CDCl_3$)

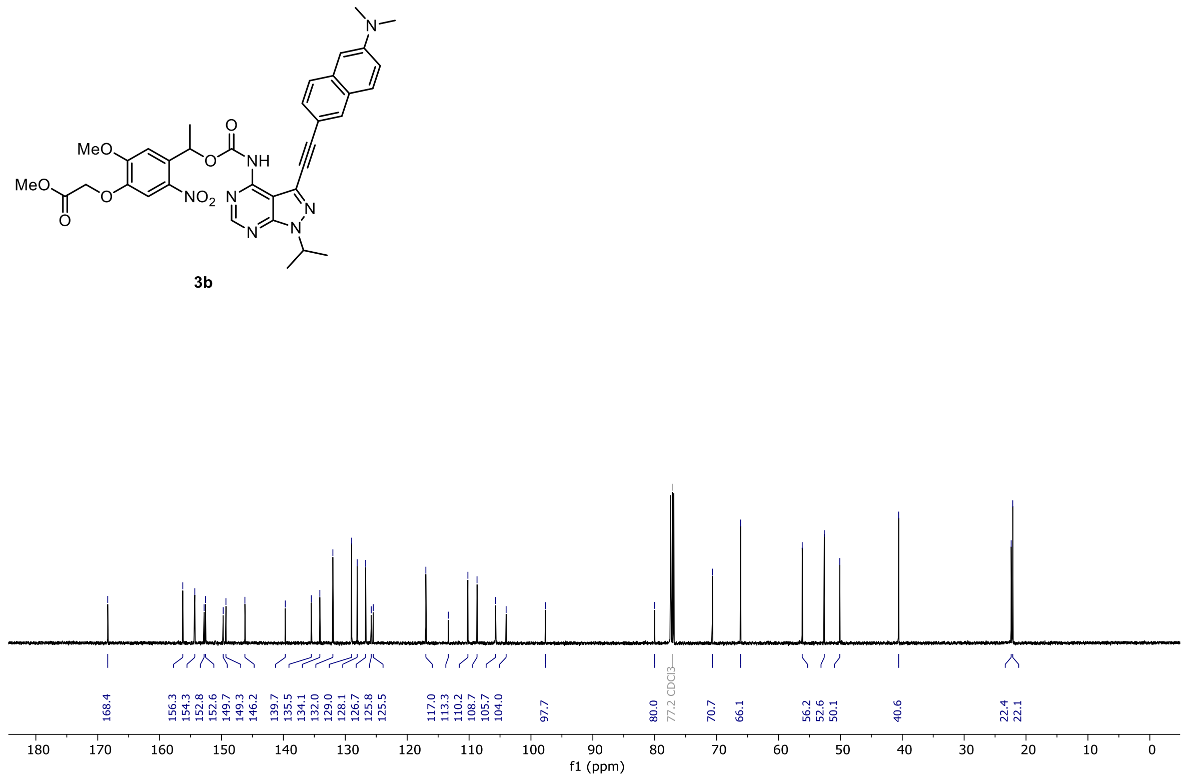

HSQC spectrum of **3b** ($CDCl_3$)

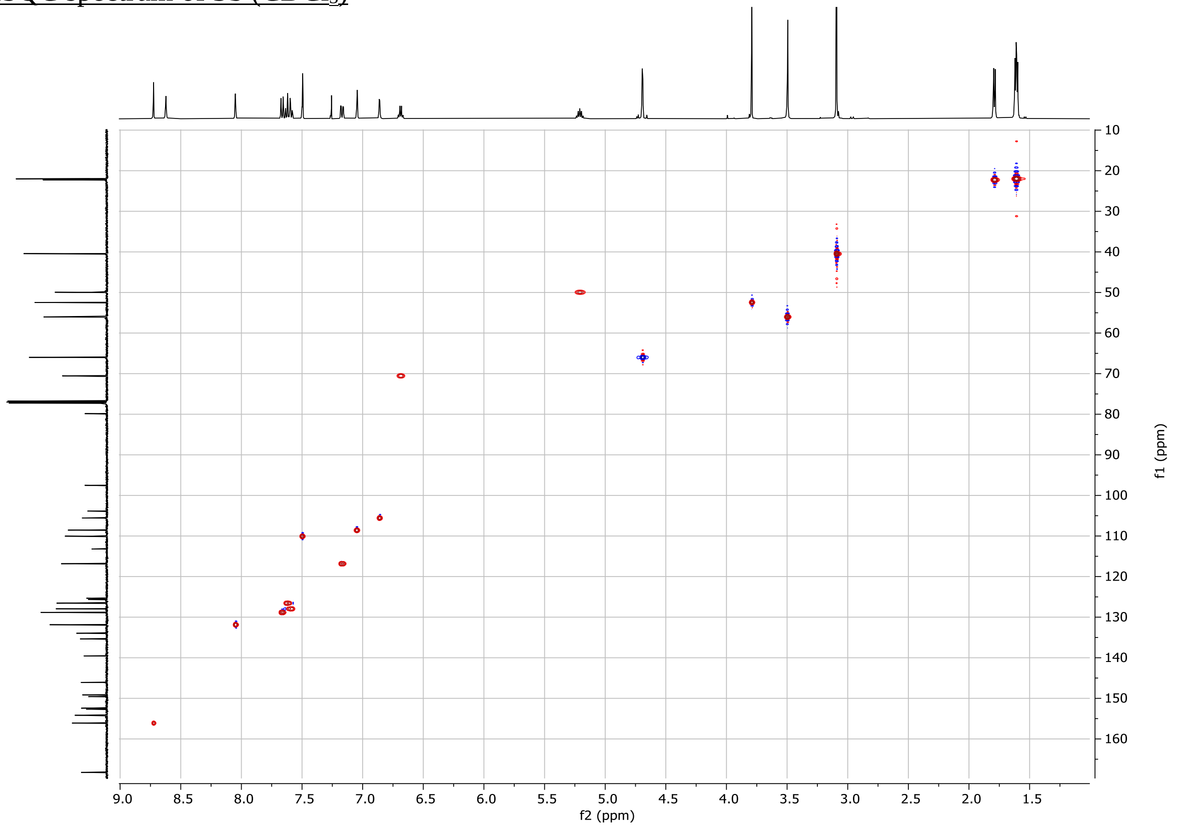

HMBC spectrum of **3b** ($CDCl_3$)

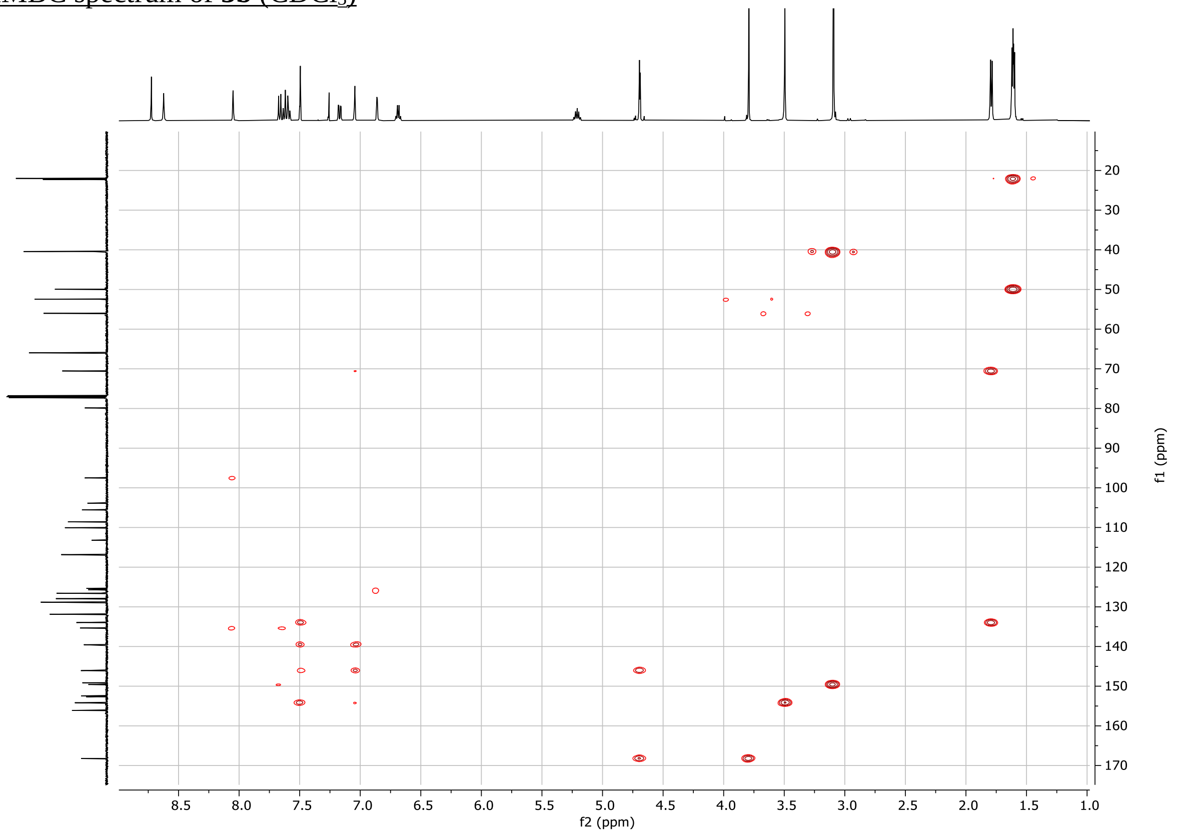

^{1}H NMR spectrum of **4b** (400 MHz, DMSO-d_6)

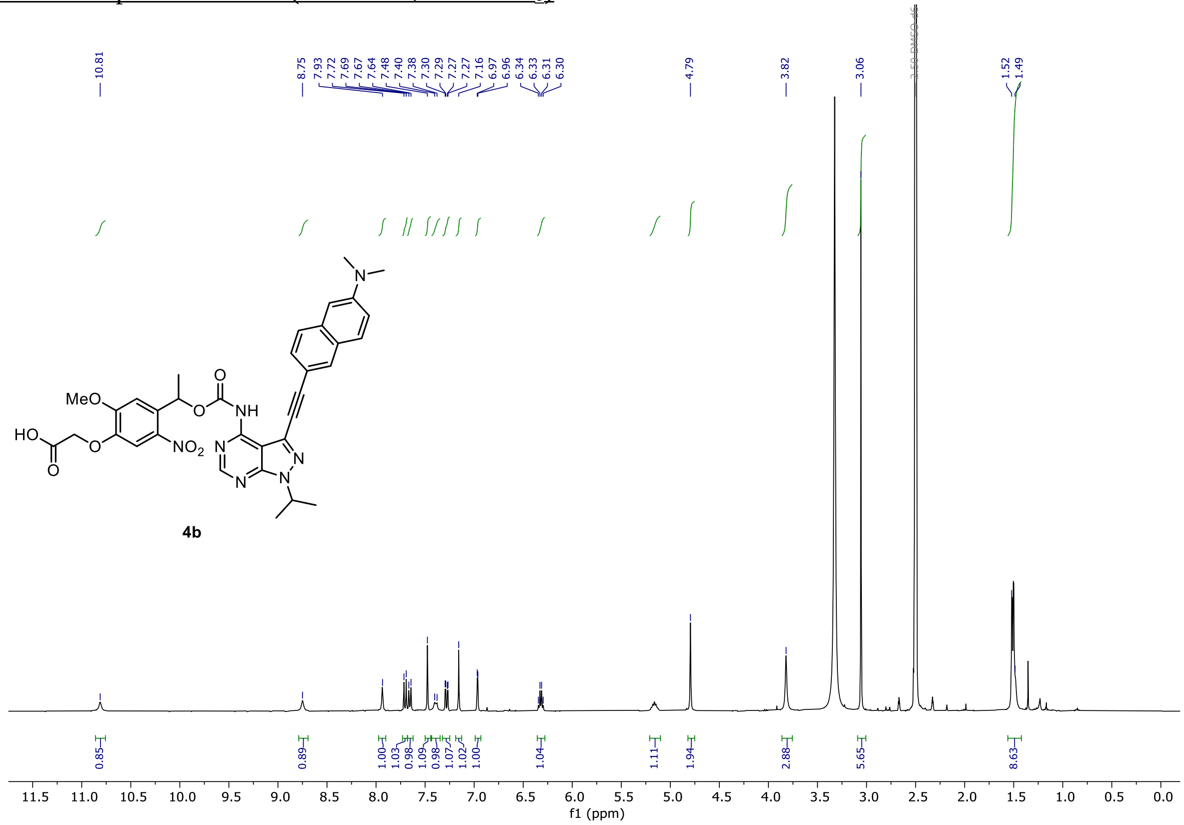

^{13}C NMR spectrum of **4b** (101 MHz, DMSO-d_6)

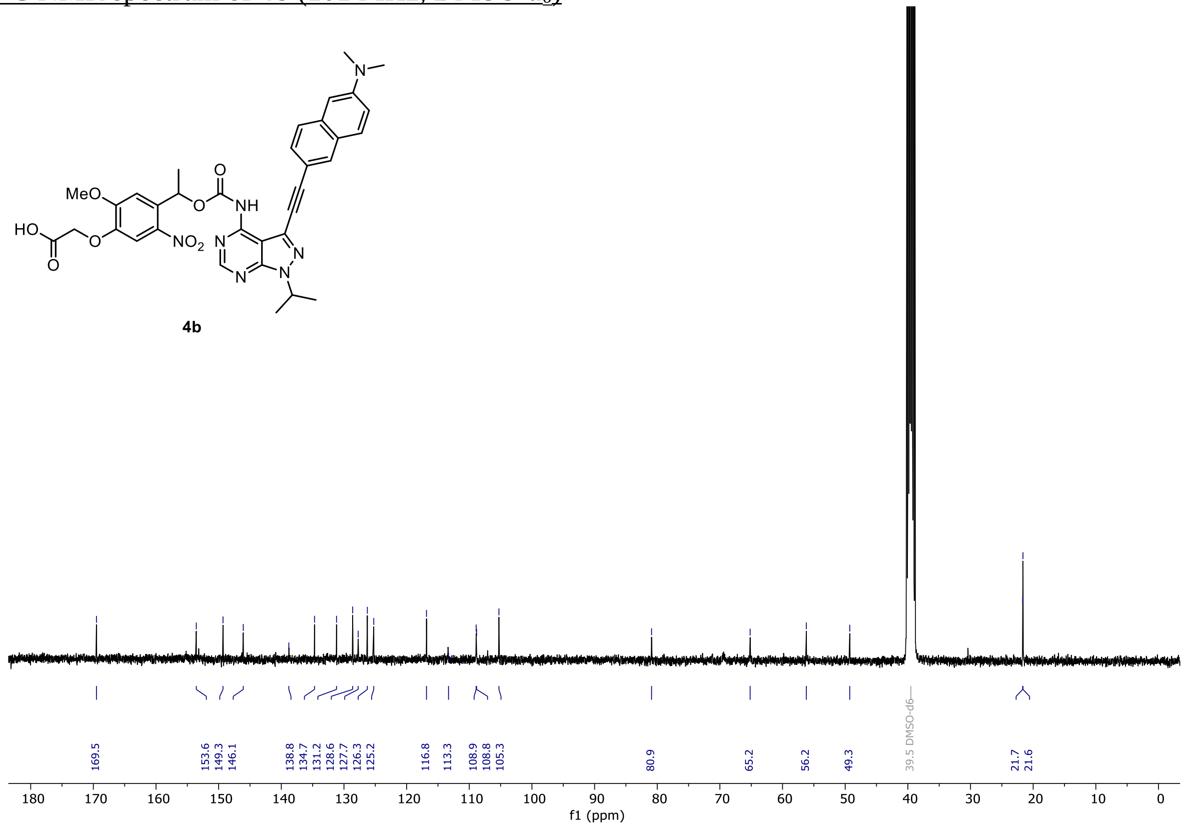

HSQC spectrum of **4b** (DMSO-d_6)

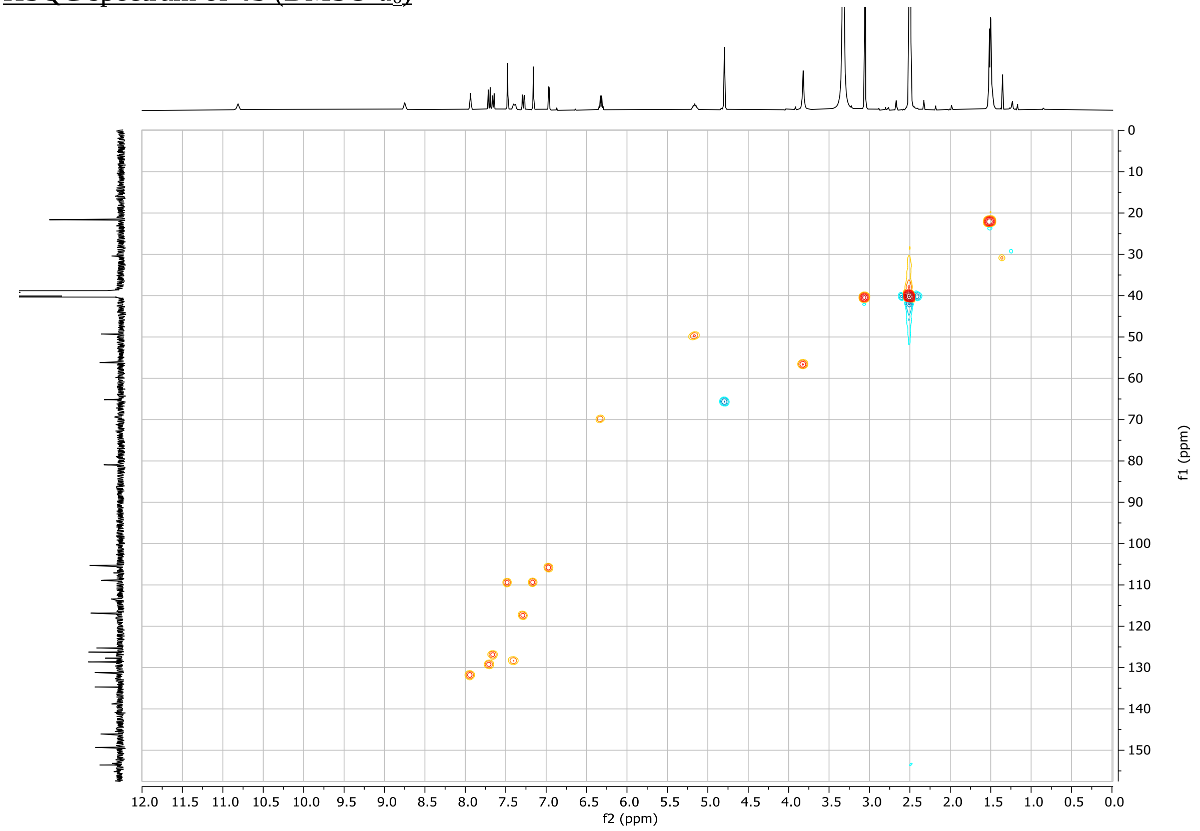

HMBC spectrum of **4b** (DMSO-d_6)

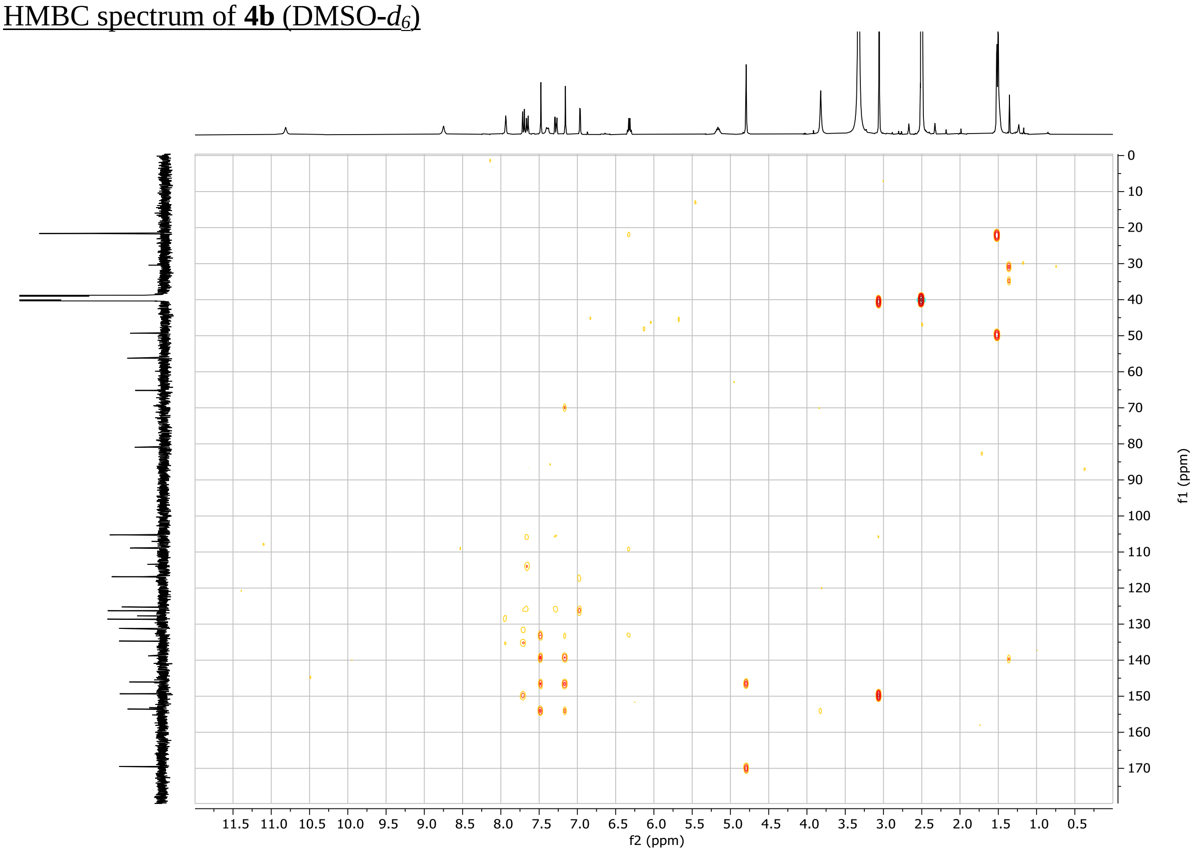

^{1}H NMR spectrum of **S10** (400 MHz, $CDCl_3$)

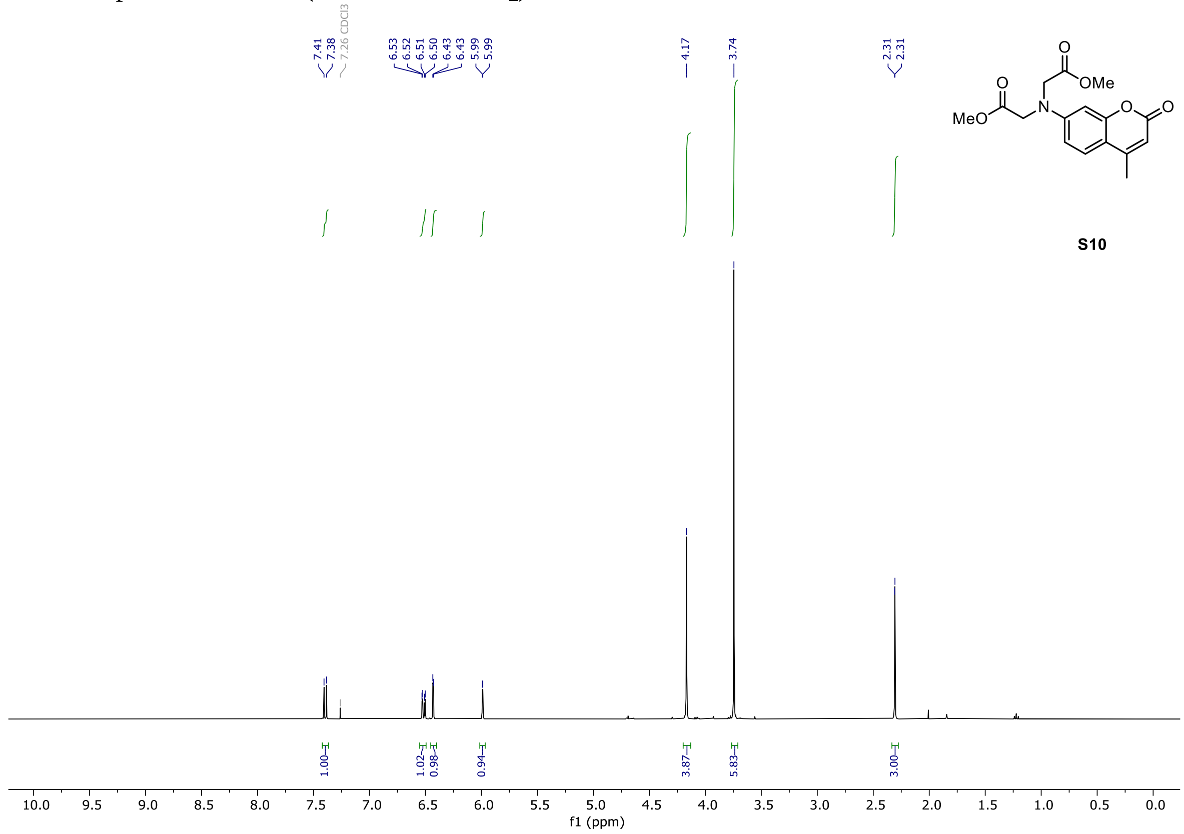

^{13}C NMR spectrum of **S10** (101 MHz, $CDCl_3$)

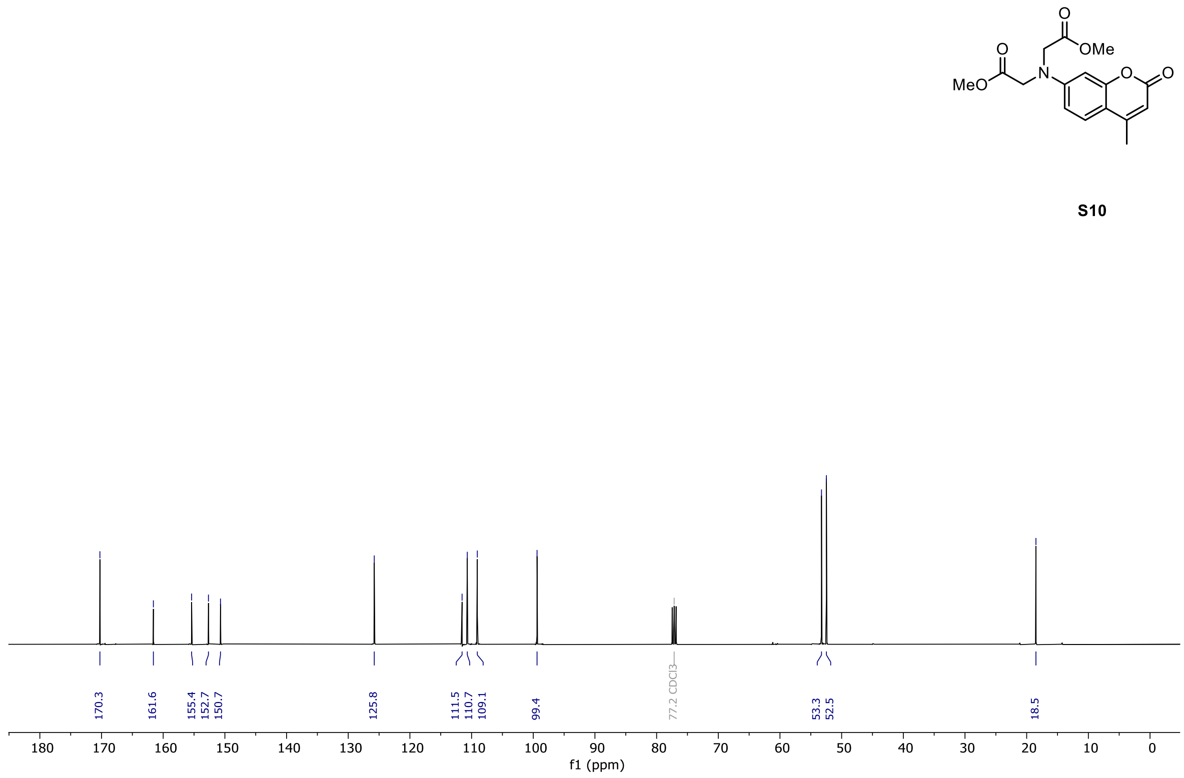

^1H NMR spectrum of **S12** (400 MHz, DMSO-d_6)

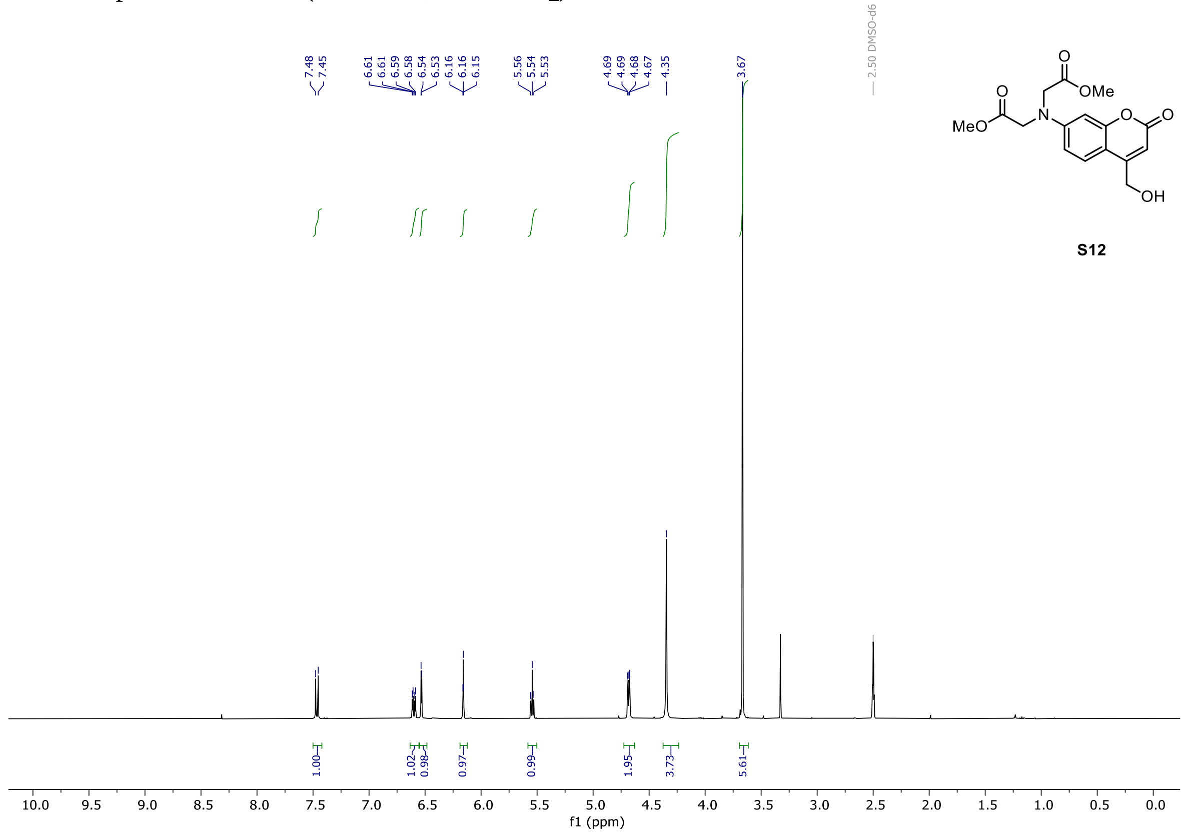

S12

^1H NMR spectrum of **S12** (400 MHz, $CDCl_3$)

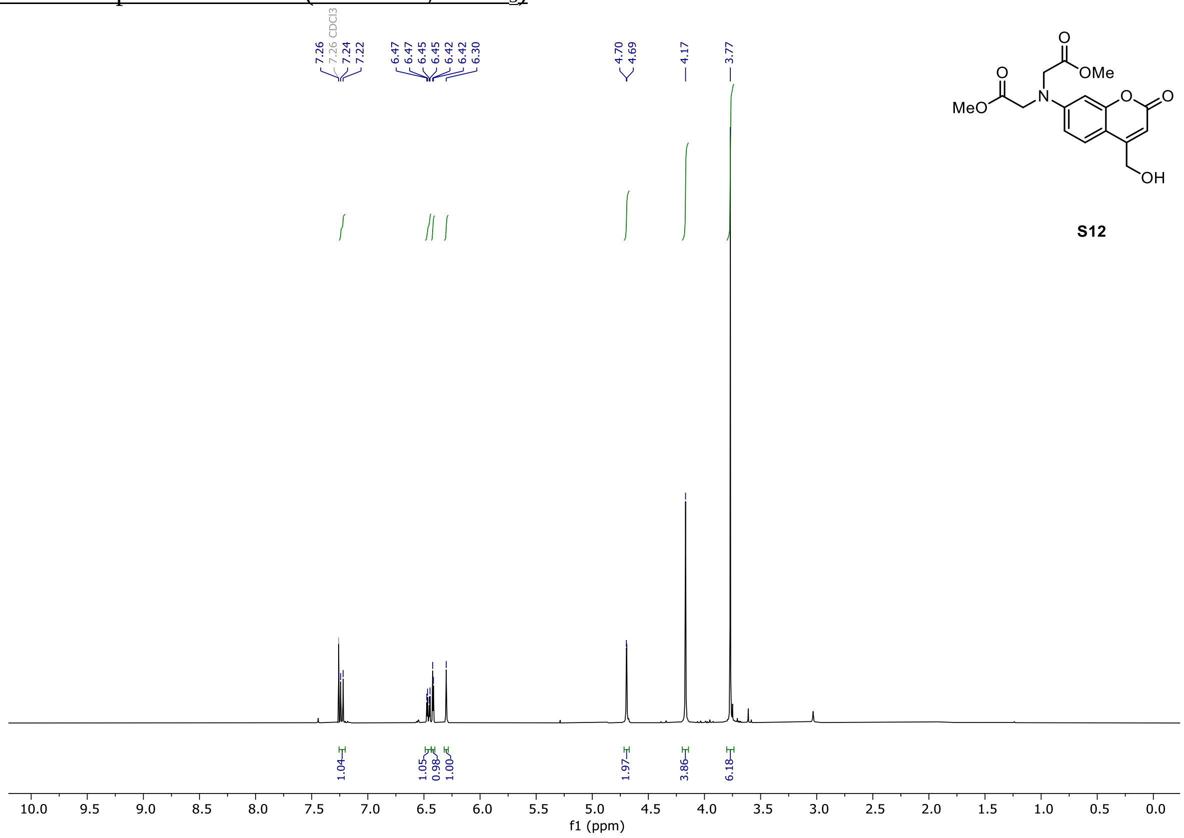

S12

^{13}C NMR spectrum of **S12** (101 MHz, $CDCl_3$)

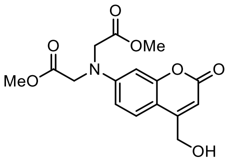

S12

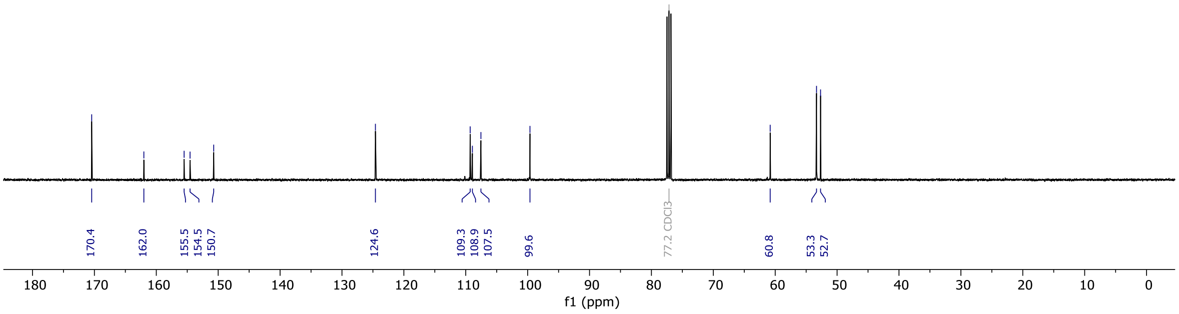

^{1}H NMR spectrum of **2c** (400 MHz, $CDCl_3$)

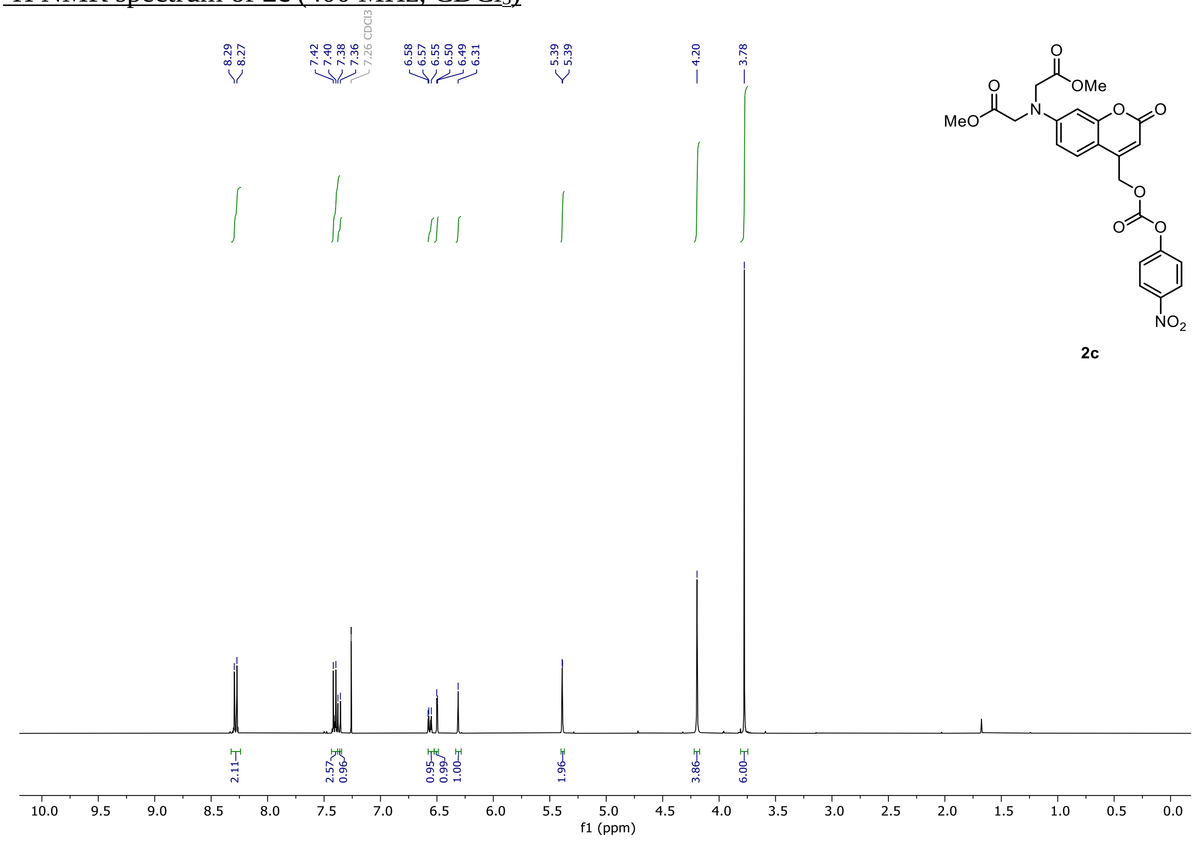

2c

^{13}C NMR spectrum of **2c** (101 MHz, $CDCl_3$)

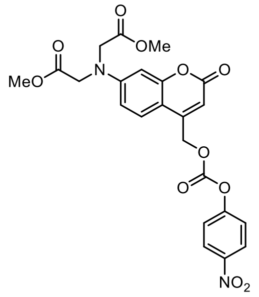

2c

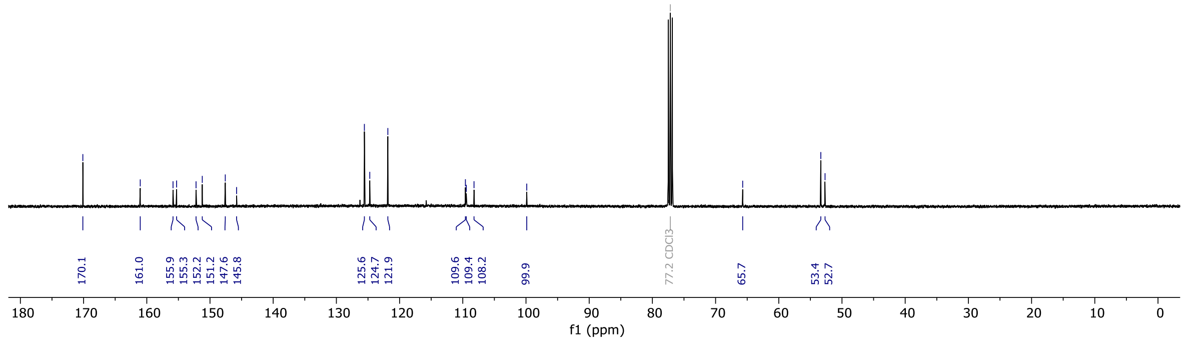

^{1}H NMR spectrum of **3c** (400 MHz, $CDCl_3$)

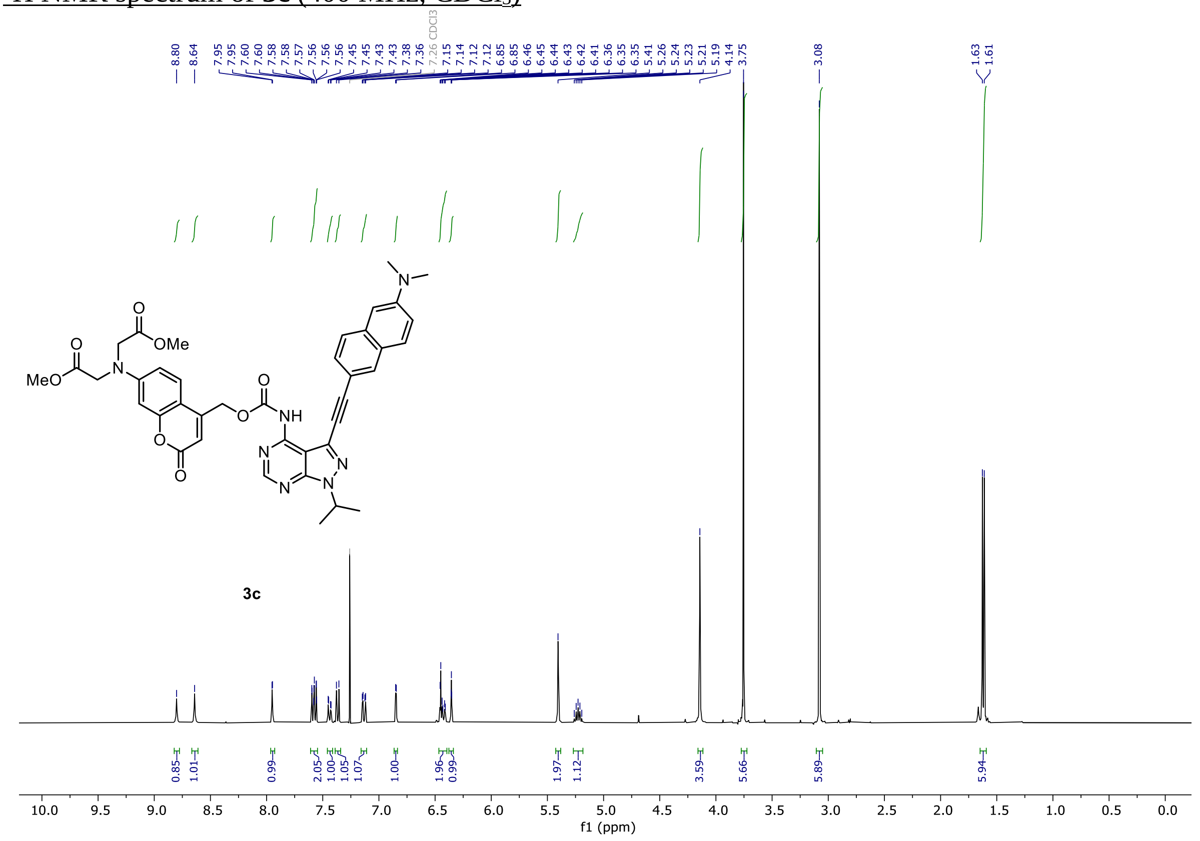

^{13}C NMR spectrum of **3c** (101 MHz, $CDCl_3$)

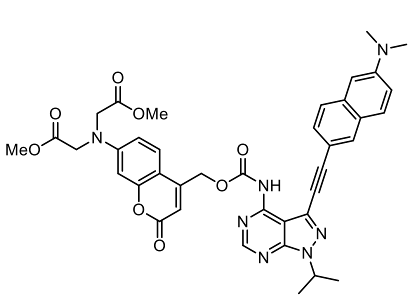

3c

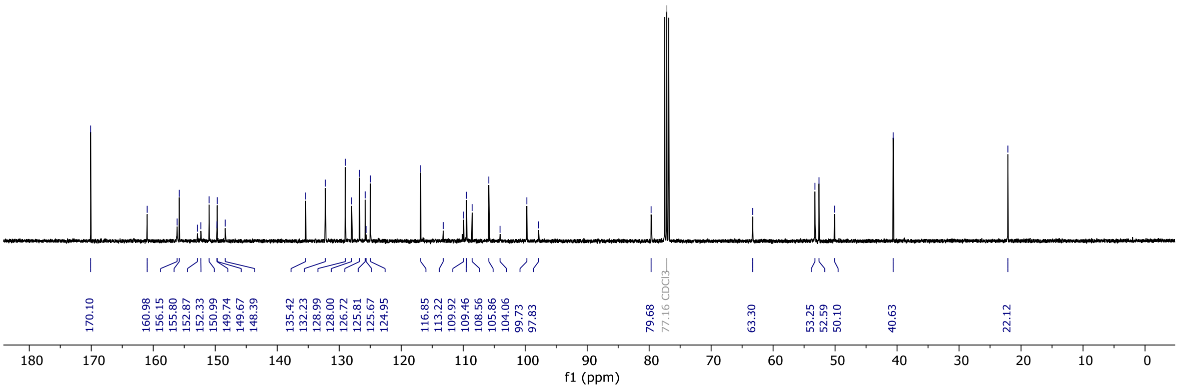

HSQC spectrum of **3c** ($CDCl_3$)

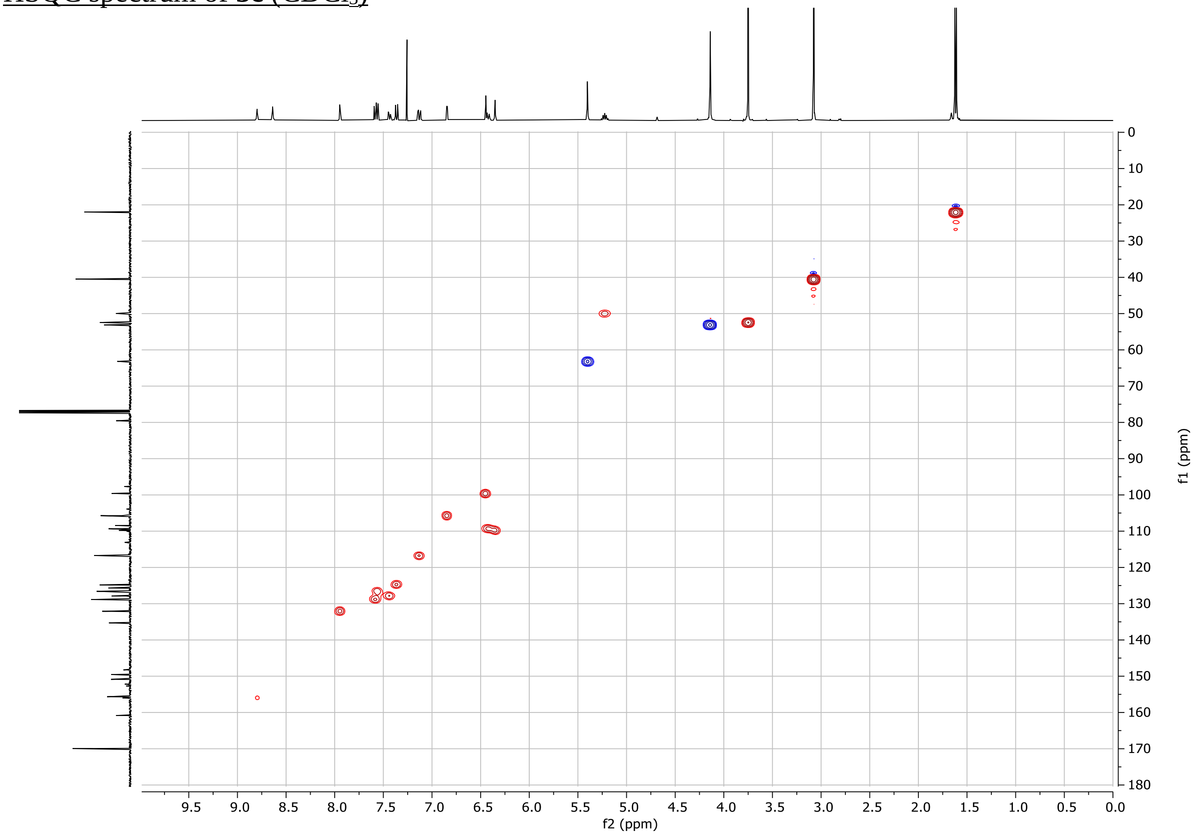

^{1}H NMR spectrum of compound **S14** (400 MHz, $CDCl_3$)

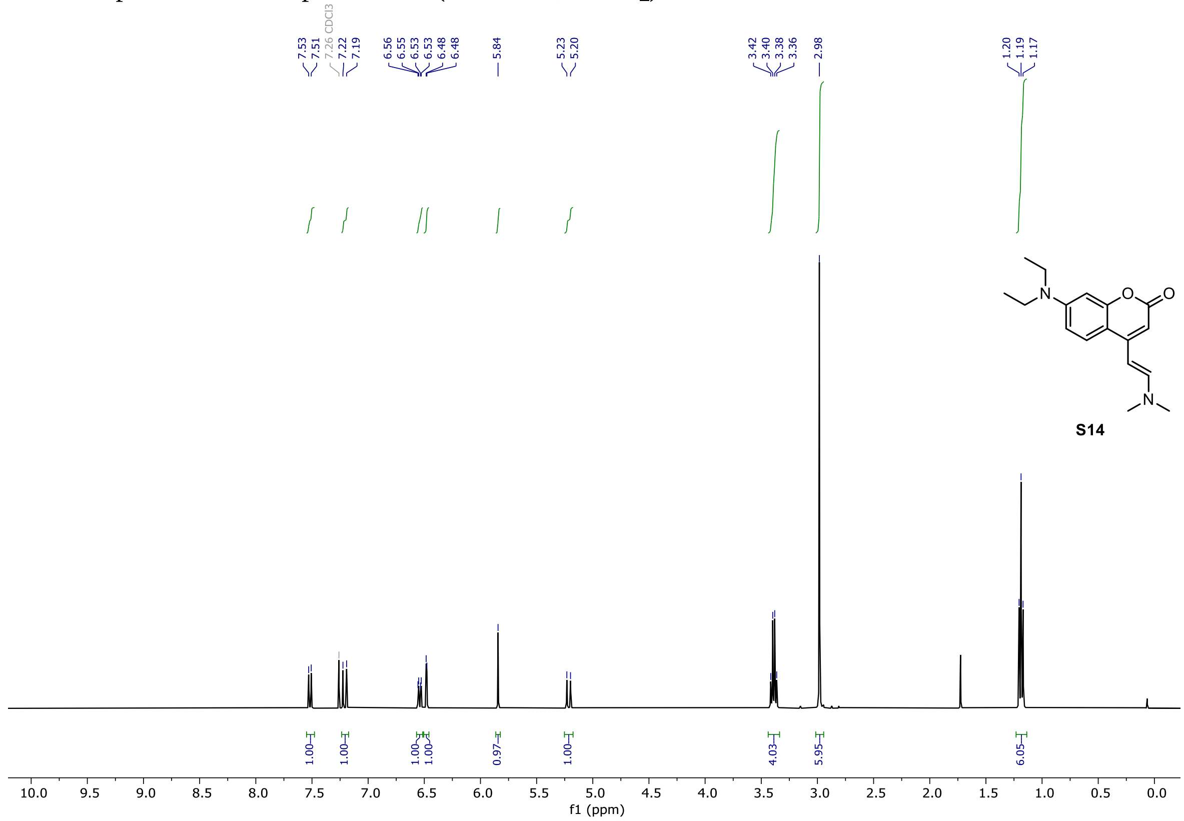

^{1}H NMR spectrum of compound **S15** (400 MHz, $CDCl_3$)

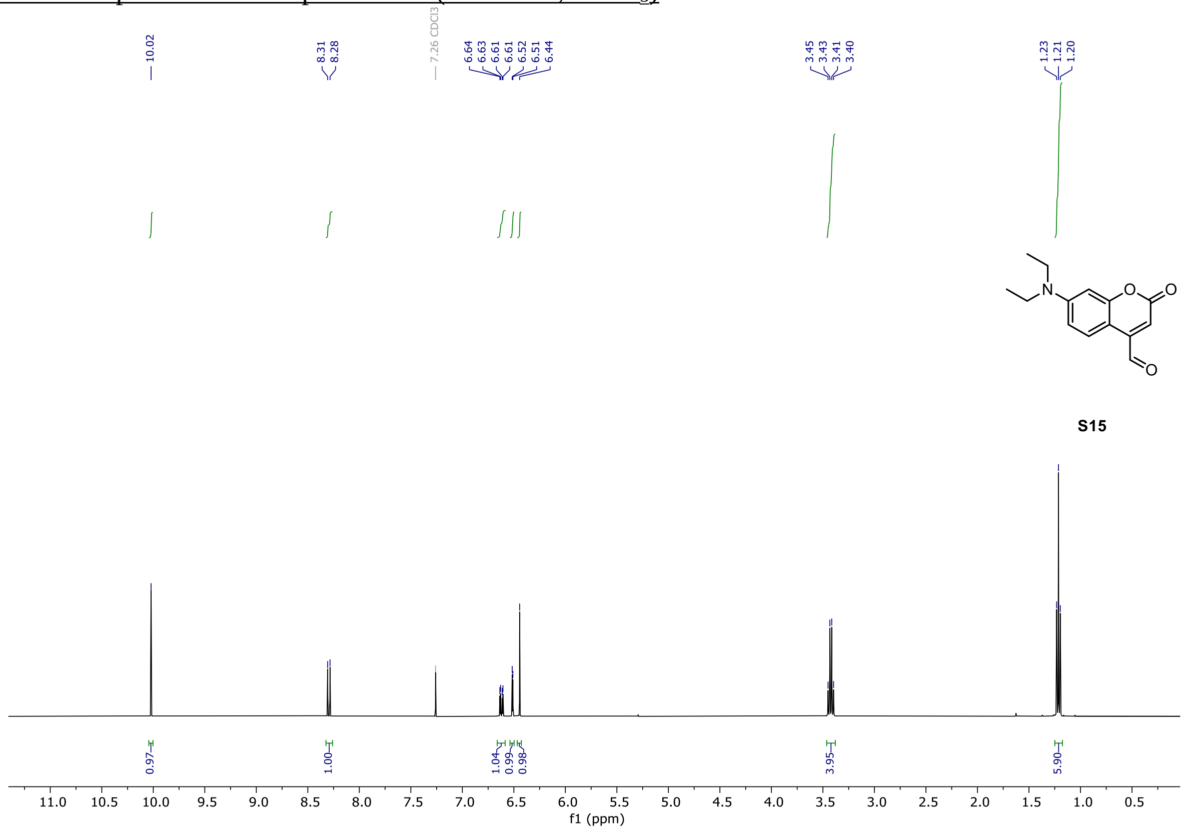

^{1}H NMR spectrum of compound **S16** (400 MHz, $CDCl_3$)

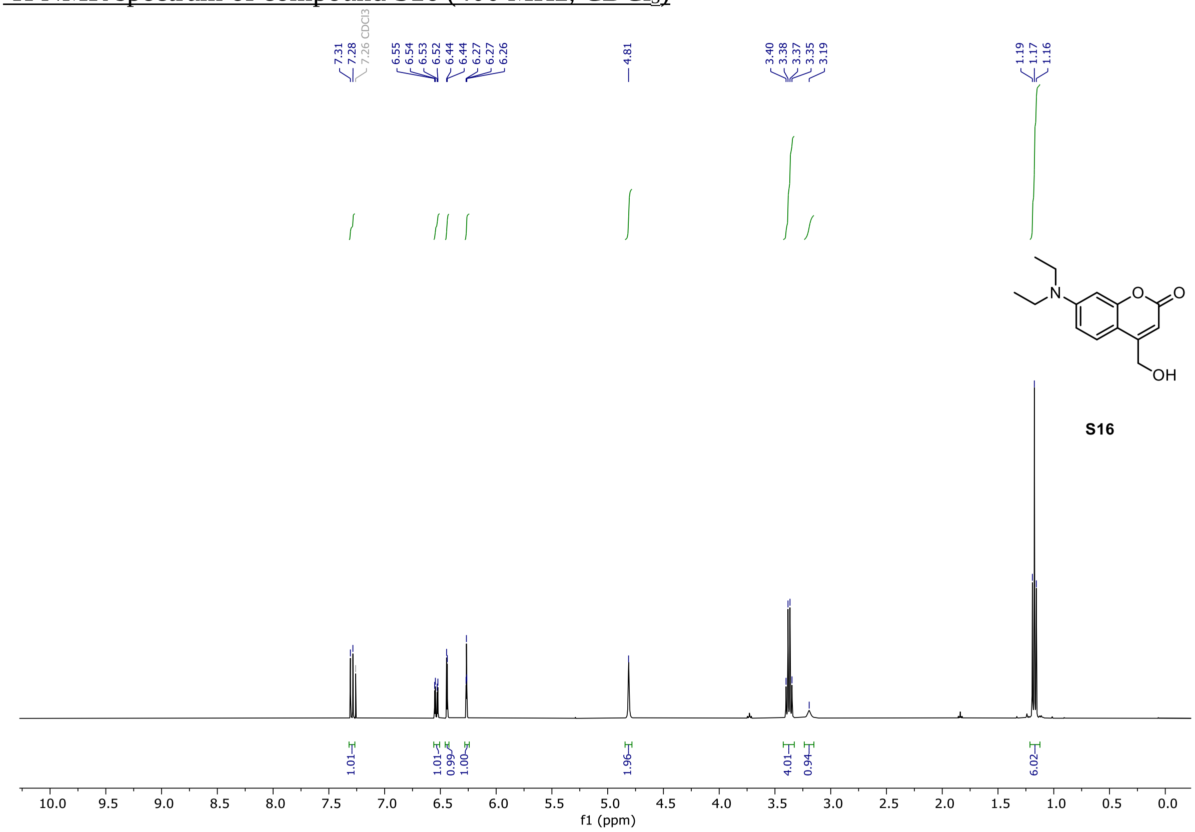

^{1}H NMR spectrum of compound **2d** (400 MHz, $CDCl_3$)

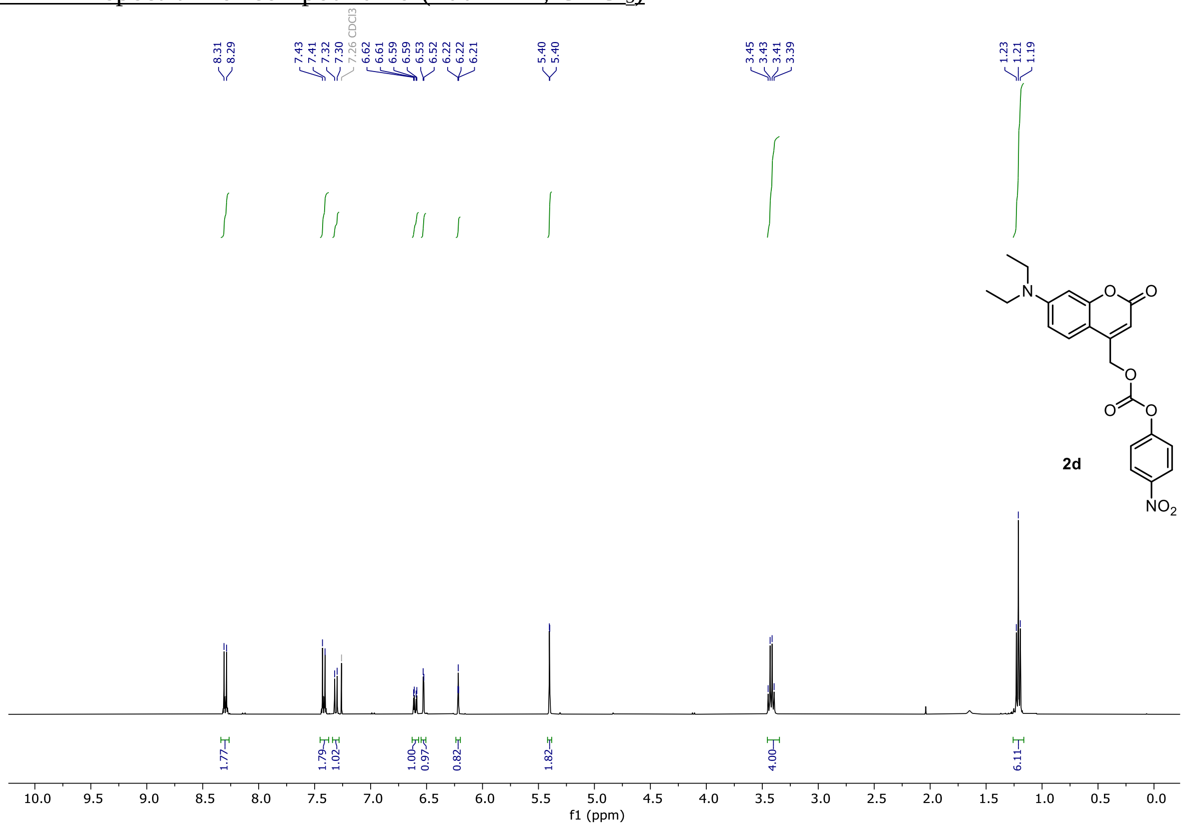

^{1}H NMR spectrum of **3d** (400 MHz, $CDCl_3$)

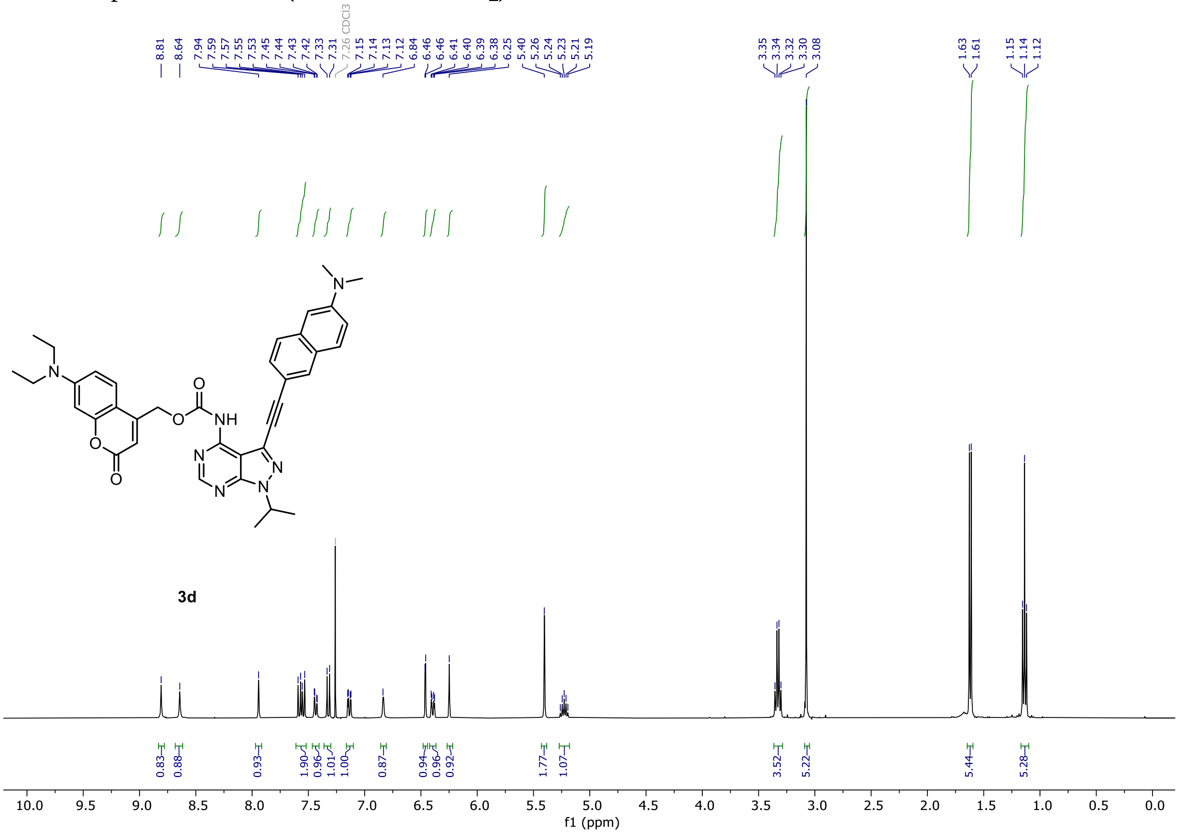

^{13}C NMR spectrum of **3d** (101 MHz, $CDCl_3$)

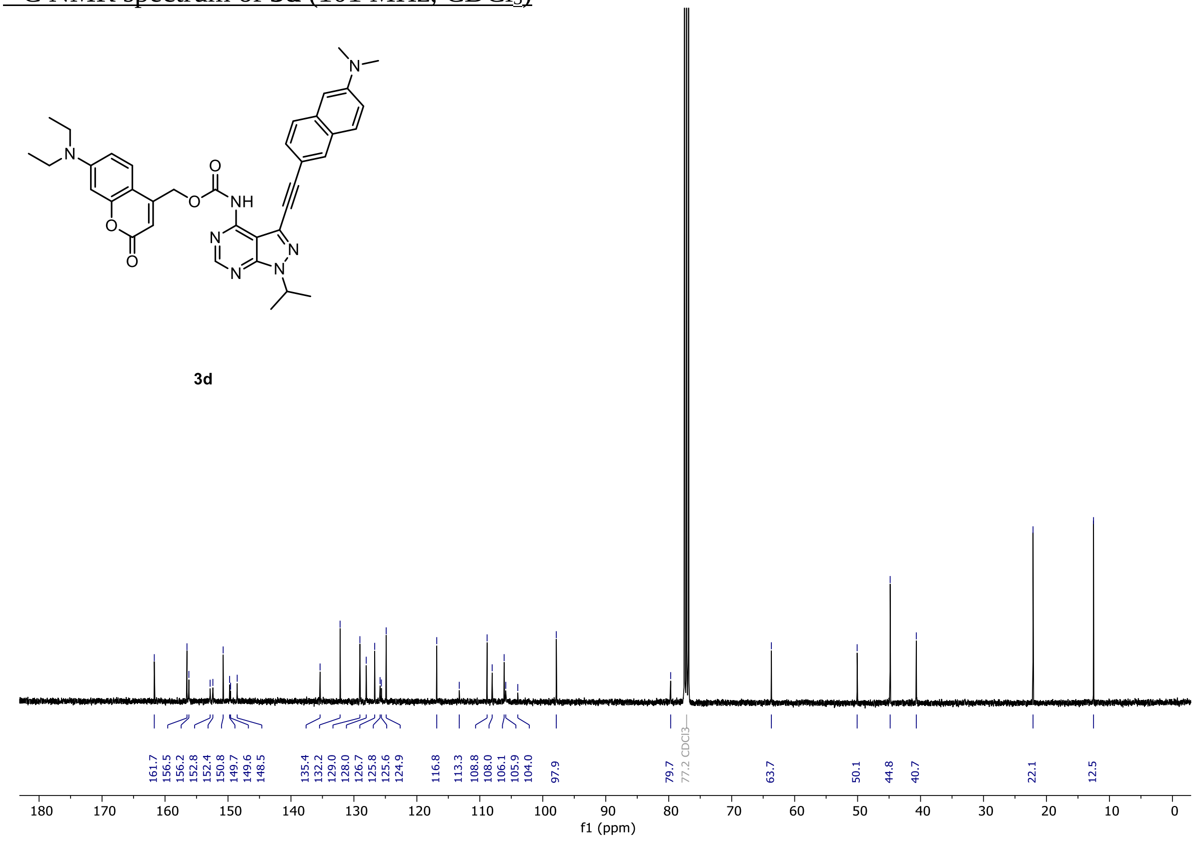

HSQC spectrum of **3d** ($CDCl_3$)

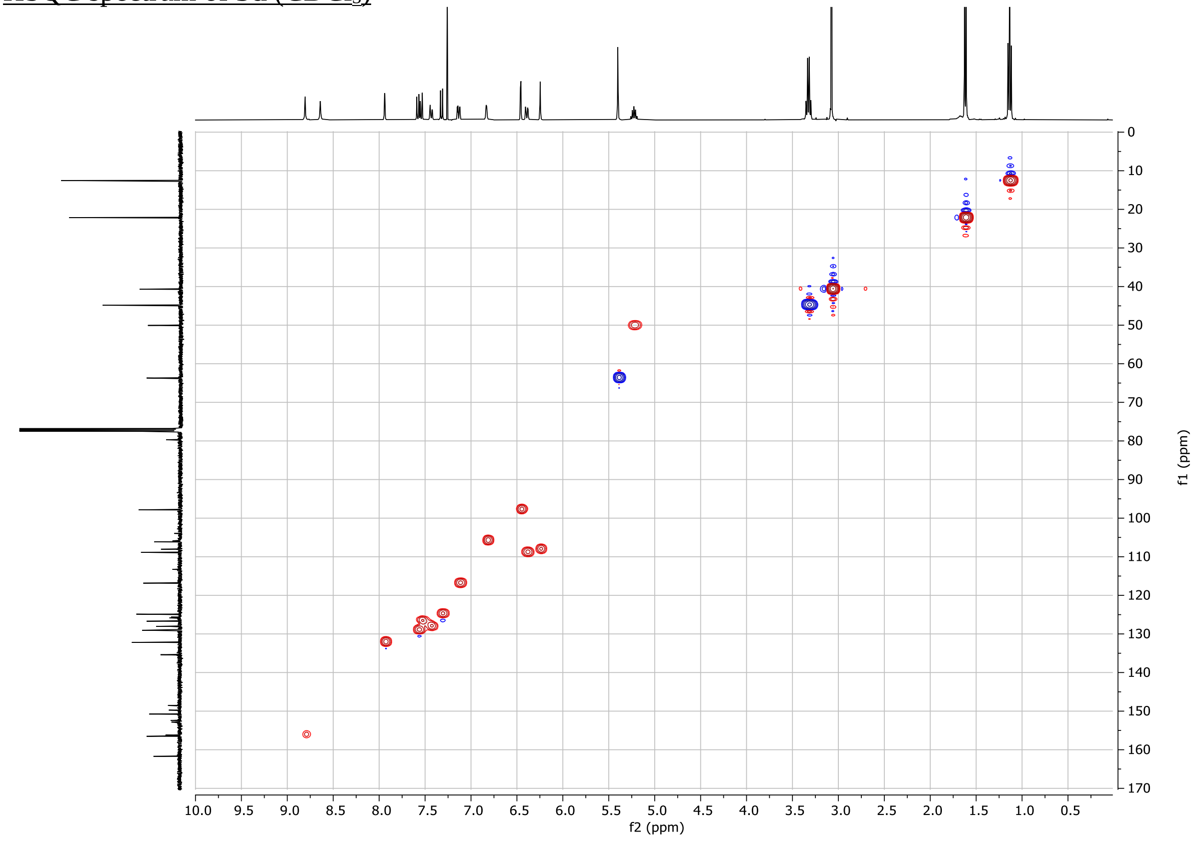

HMBC spectrum of **3d** ($CDCl_3$)

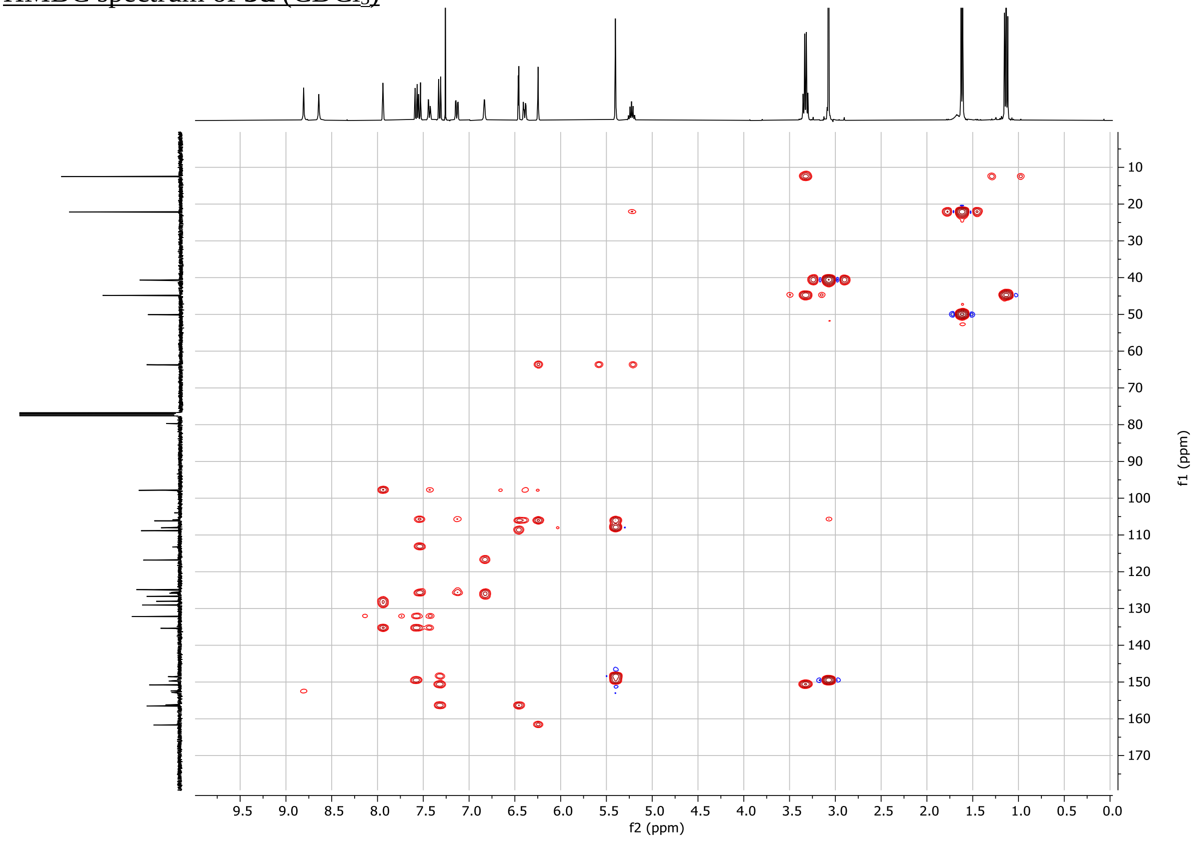

^{1}H NMR spectrum of **S19** (400 MHz, $CDCl_3$)

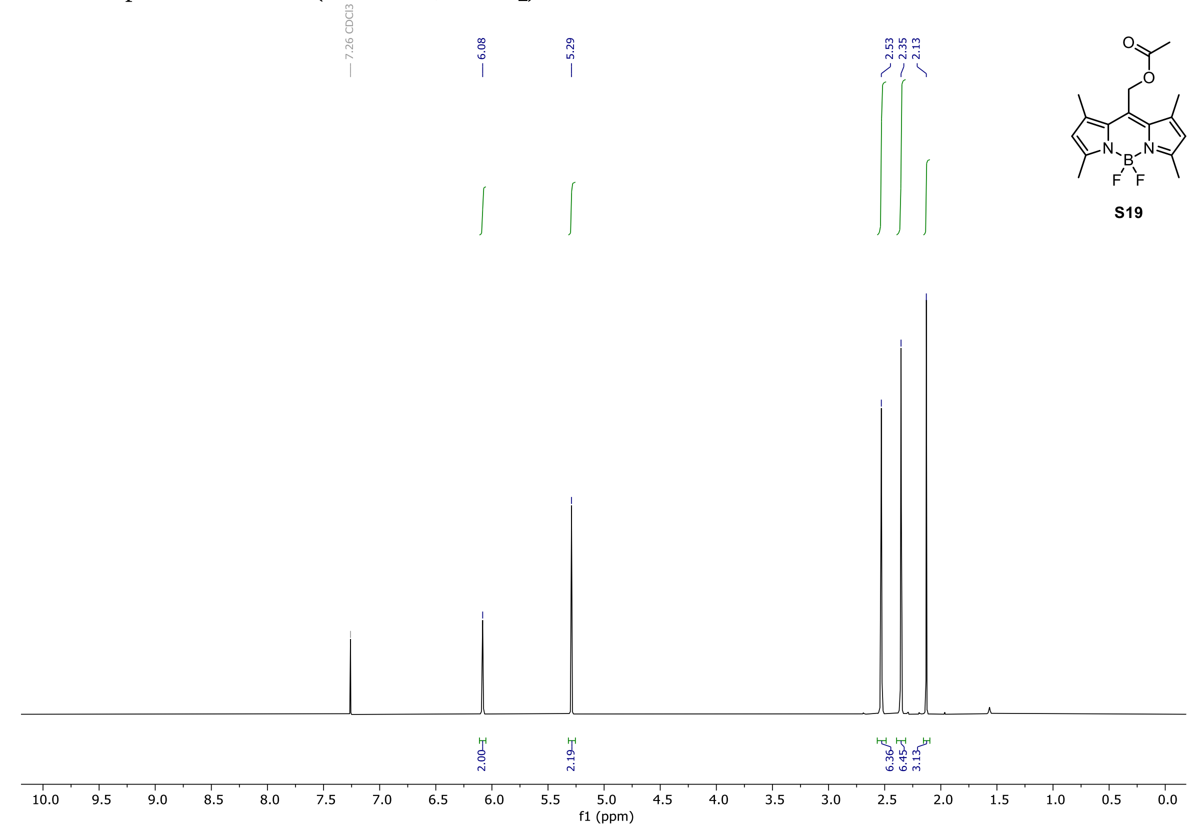

^{1}H NMR spectrum of **2e** (400 MHz, $CDCl_3$)

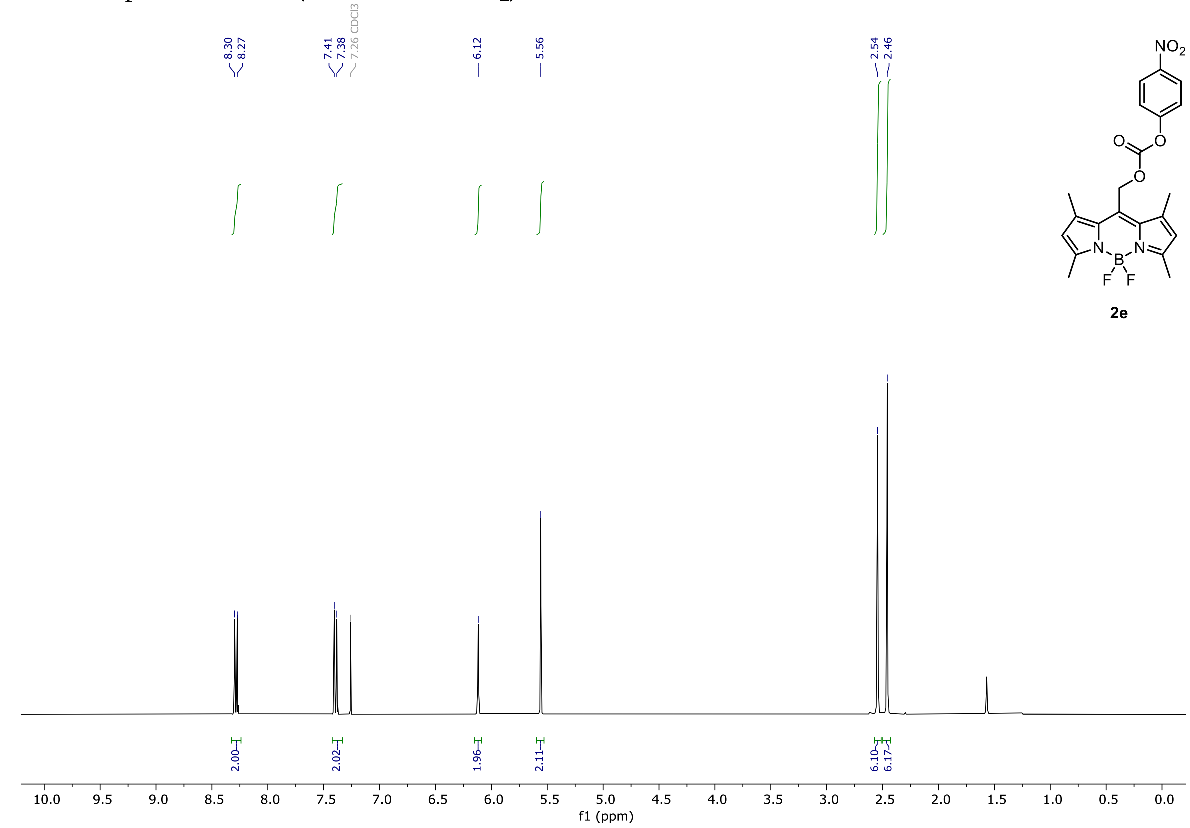

^{1}H NMR spectrum of **3e** (400 MHz, $CDCl_3$)

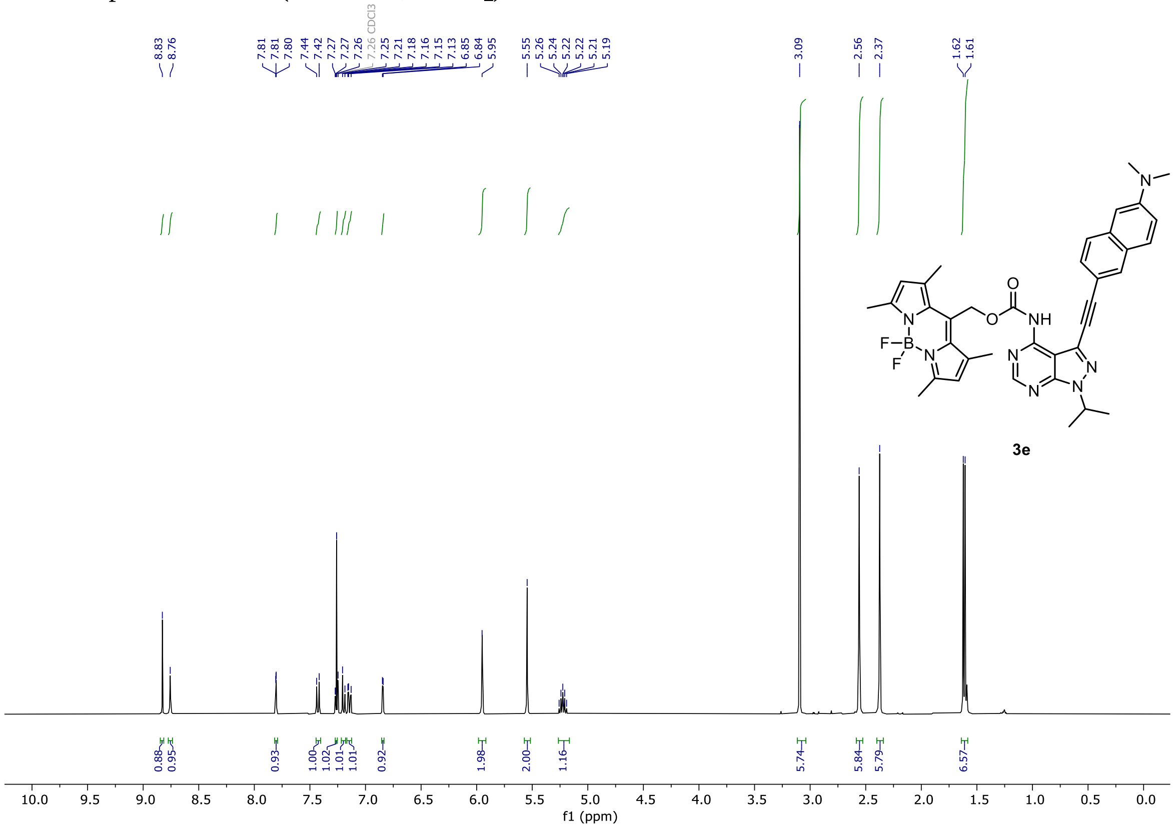

^{13}C NMR spectrum of **3e** (101 MHz, $CDCl_3$)

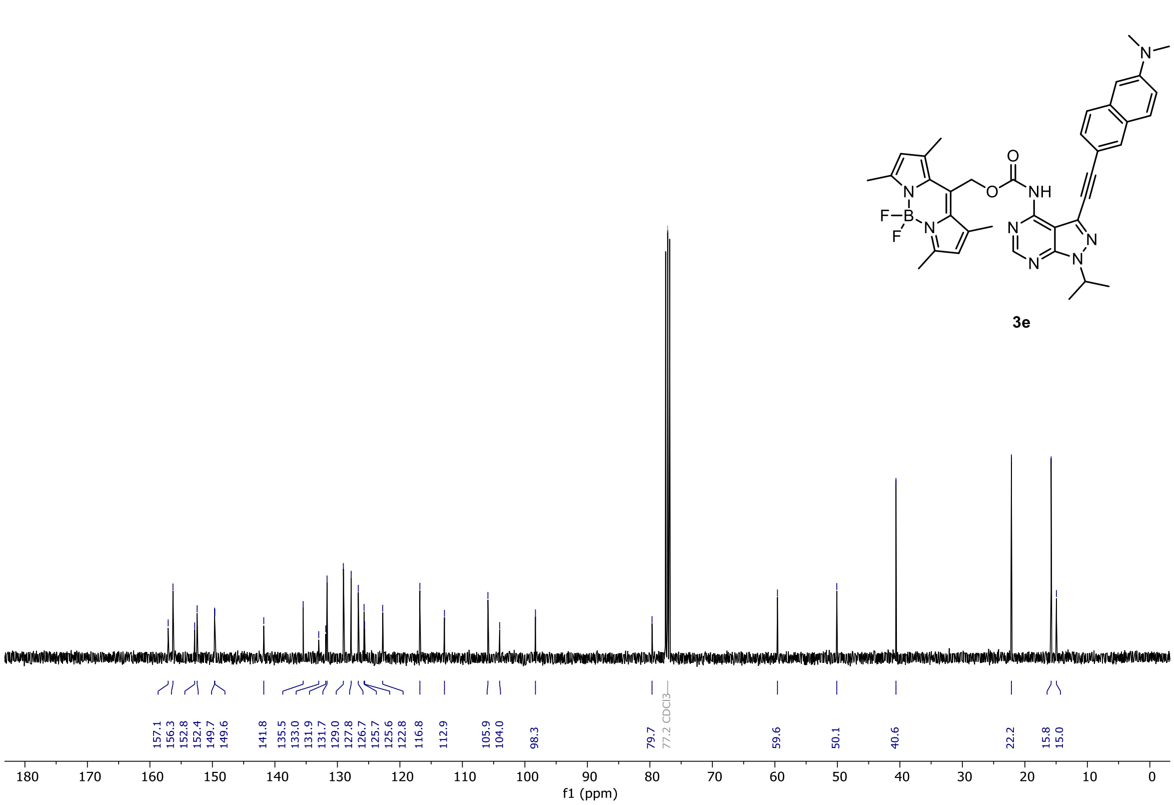

HSQC spectrum of **3e** ($CDCl_3$)

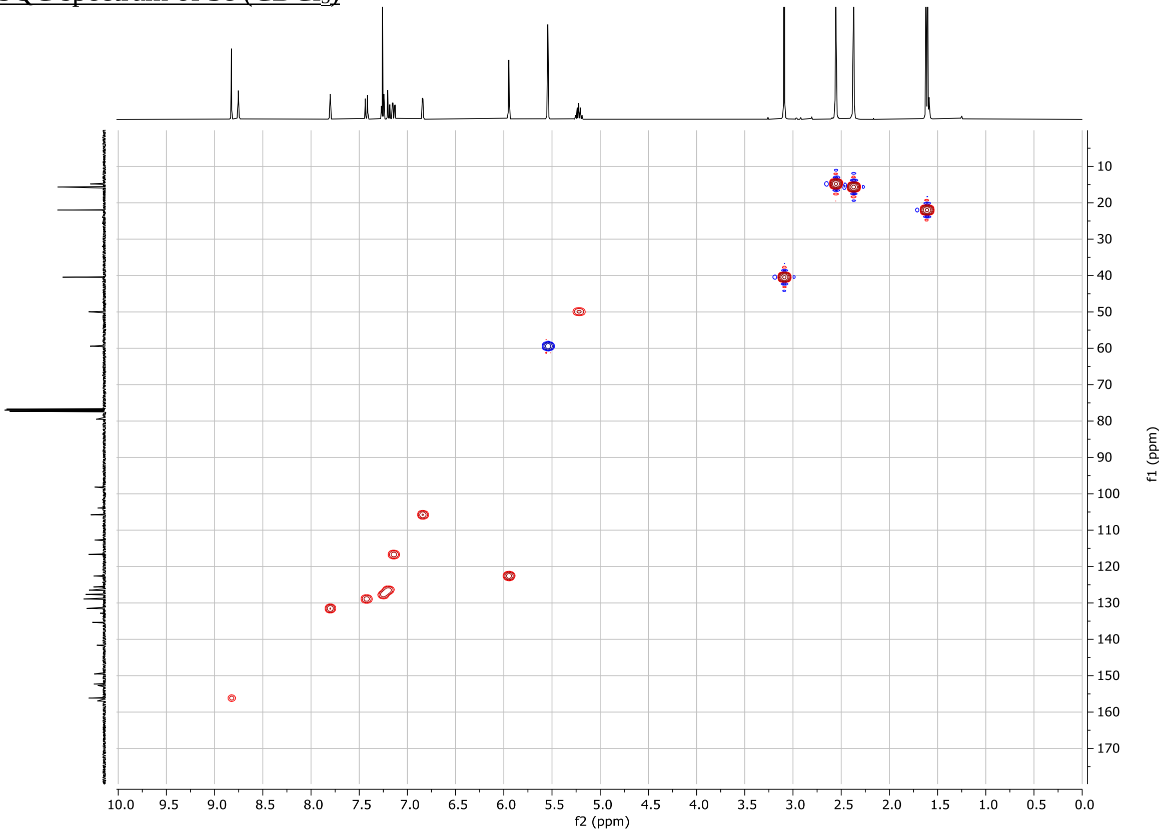

HMBC spectrum of **3e** ($CDCl_3$)

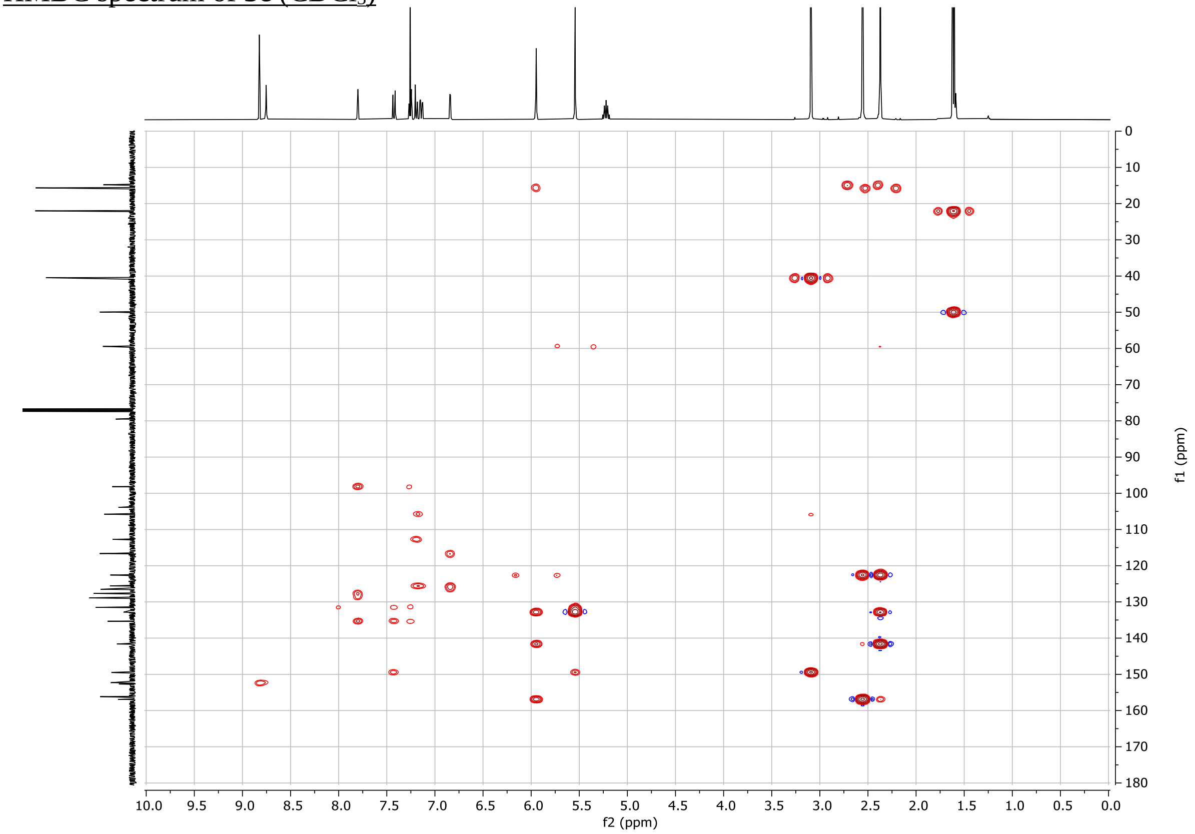

Photophysical Characterisation of Compounds 4a, 4b and 3c–e.

The DMSO used for photophysical characterisation was of spectroscopic grade. The aqueous solution used for photophysical characterisation consisted of 10% DMSO in mQ-water, air-equilibrated. Ground state absorption spectra were collected using a Varian CaryBio 50 UV/vis spectrophotometer. All steady-state fluorescent measurements were recorded on a SPEX Fluorolog-3 spectrofluorometer (JY Hariba). Samples were recorded in a macro quartz cuvette (light path = 10 × 10 mm) or for the titration experiments, in a reduced volume cuvette (v = 45 µL, light path = 3 × 3 mm). Fluorescence quantum yields were determined using 9,10-diphenylanthracene (DPA) in cyclohexane (Φ_F = 0.97) as a reference. All quantum yield values were corrected for the solvent refractive index (cyclohexane = 1.4262, 10% DMSO in H_2O = 1.347). For decaging reactions, samples were irradiated outside of the spectrophotometer in a macro quartz cuvette (light path = 10 × 10 mm). The light sources employed for irradiation were LEDs (LED Engin) centered around 365 nm (LZ1-10UV100, FWHM = 12 nm), 405 nm (LZ1-10UB00-00U8, FWHM = 19 nm) and 523 nm (LZ1-10R200, FWHM = 40 nm). Cuvettes were positioned *ca* 6 cm from the irradiation source. The power of the LEDs was determined by measuring the light intensity at the cuvette using a hand-held light meter with a diameter of 1 cm. The decaging quantum yields were determined according to standard procedures using 4,4'-dimethylazobenze (DMAB)[6] and furylfulgide (Aberchrome 540™)[7] as actinometers. Photo-induced decaging of compounds with 365 or 405 nm were referenced to DMAB ($\phi_{EZ,365}$ = 0.18, $\phi_{EZ,405}$ = 0.29), whereas Aberchrome 540 was employed when irradiating at 523 nm ($\phi_{\text{closed to open},523}$ = 0.053). Samples and standards were irradiated under identical irradiation conditions and geometry. The molar absorption coefficients were used to correct the decaging quantum yields.

Monitoring Decaging Reactions using UV/vis Absorption Spectroscopy.

Decaging reactions were evaluated in aqueous solution (*ca* 10 µM, 10% DMSO in H_2O). Samples were irradiated with LEDs at 365 nm, 405 nm or 523 nm depending on the absorption spectrum of the caged substrate. The reactions were monitored by recording absorption spectra at regular intervals.

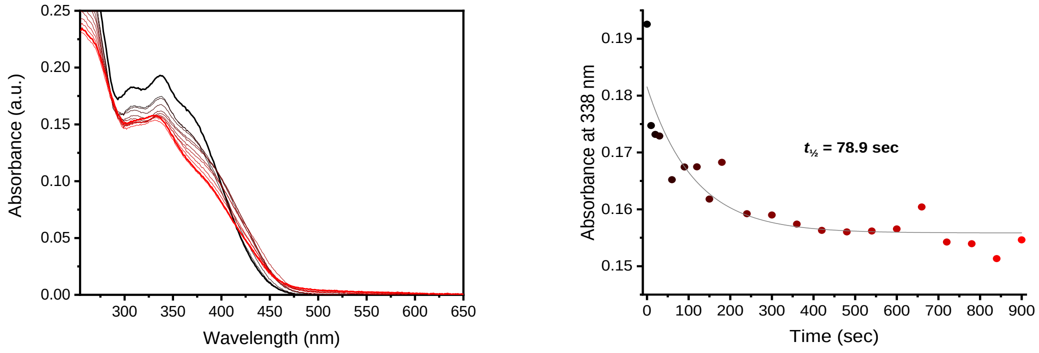

Fig. S1 (*left*) Absorption spectra of **4a** in aqueous solution before irradiation (black) and after irradiation at 365 nm (red, power = 33 mW/cm^2); (*right*) Changes in absorbance at 338 nm upon irradiation at 365 nm (0–900 seconds).

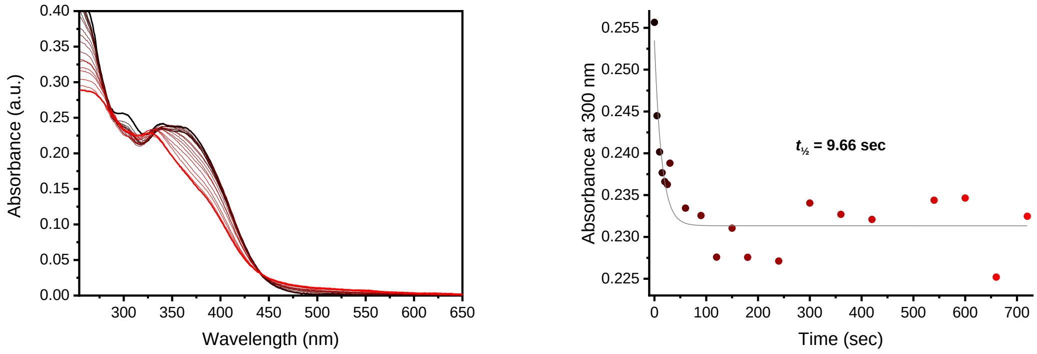

Fig. S2 (*left*) Absorption spectra of **4b** in aqueous solution before irradiation (black) and after irradiation at 365 nm (red, power = 35 mW/cm^2); (*right*) Changes in absorbance at 300 nm upon irradiation at 365 nm (0–700 seconds).

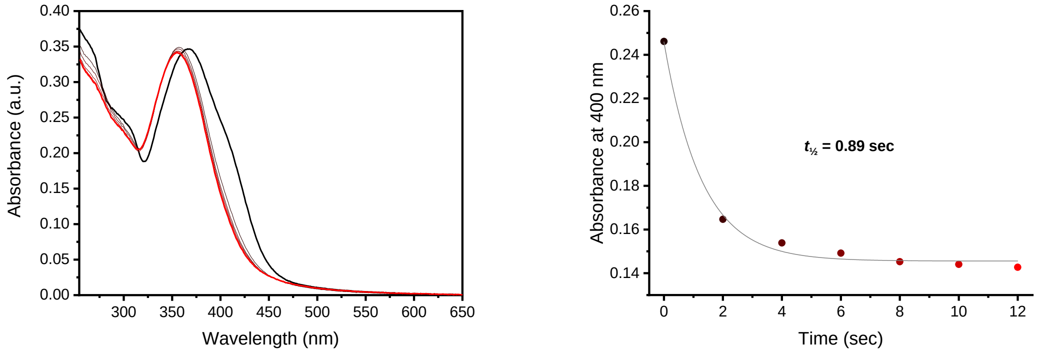

Fig. S3 (*left*) Absorption spectra of **3c** in aqueous solution before irradiation (black) and after irradiation at 365 nm (red, power = 34 mW/cm^2); (*right*) Changes in absorbance at 400 nm upon irradiation at 365 nm (0–12 seconds).

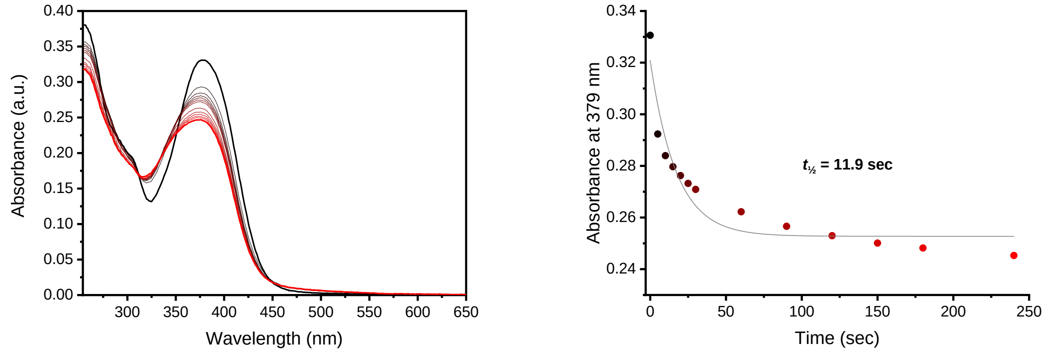

Fig. S4 (*left*) Absorption spectra of **3d** in aqueous solution before irradiation (black) and after irradiation at 405 nm (red, power = 27 mW/cm^2); (*right*) Changes in absorbance at 379 nm upon irradiation at 405 nm (0–240 seconds).

Photostability of Compound 1.

The stability of compound **1** towards irradiation with LEDs at 365 nm, 405 nm and 523 nm in both aqueous solution (*ca* 10 µM, 20% DMSO/H_2O) and DMSO (*ca* 10 µM) was monitored by recording absorption spectra at regular intervals.

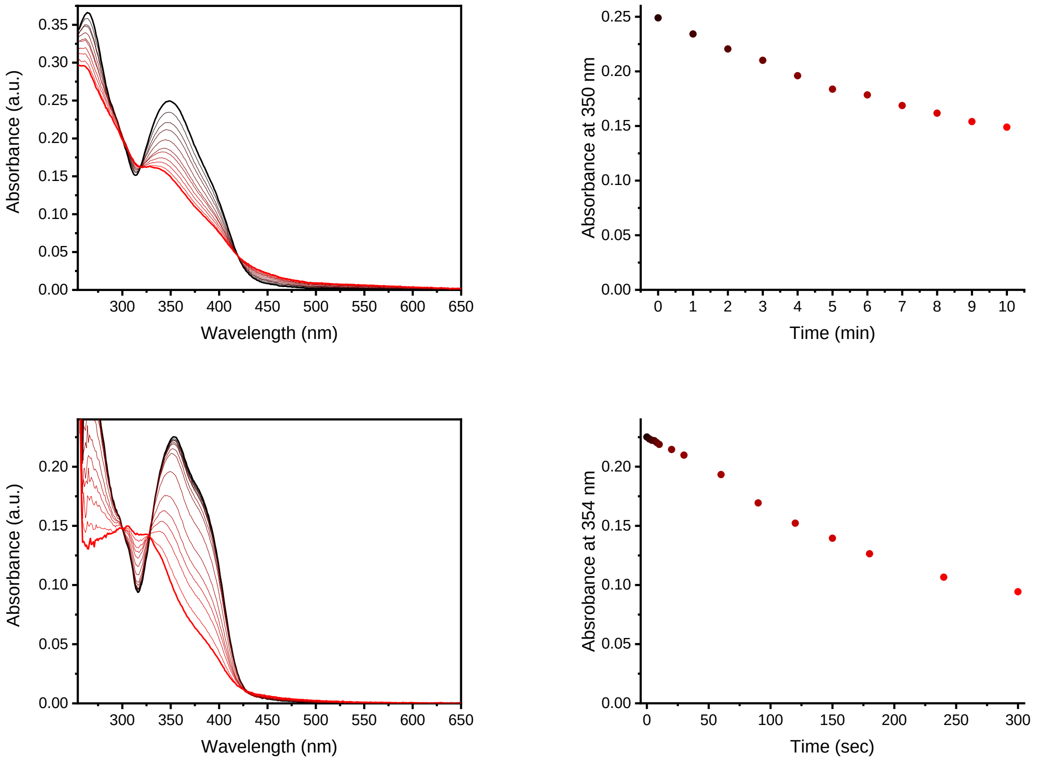

Fig. S5 (*top left*) Absorption spectra of **1** in aqueous solution before irradiation (black) and after irradiation at 365 nm (red, power = 28 mW/cm^2); (*top right*) Changes in absorbance at 350 nm upon irradiation at 365 nm (0–10 minutes); (*bottom left*) Absorption spectra of **1** in DMSO before irradiation (black) and after irradiation at 365 nm (red, power = 31 mW/cm^2); (*bottom right*) Changes in absorbance at 354 nm upon irradiation at 365 nm (0–300 seconds).

Significant photodecomposition of compound **1** is observed when irradiated at 365 nm in both aqueous solution and DMSO.

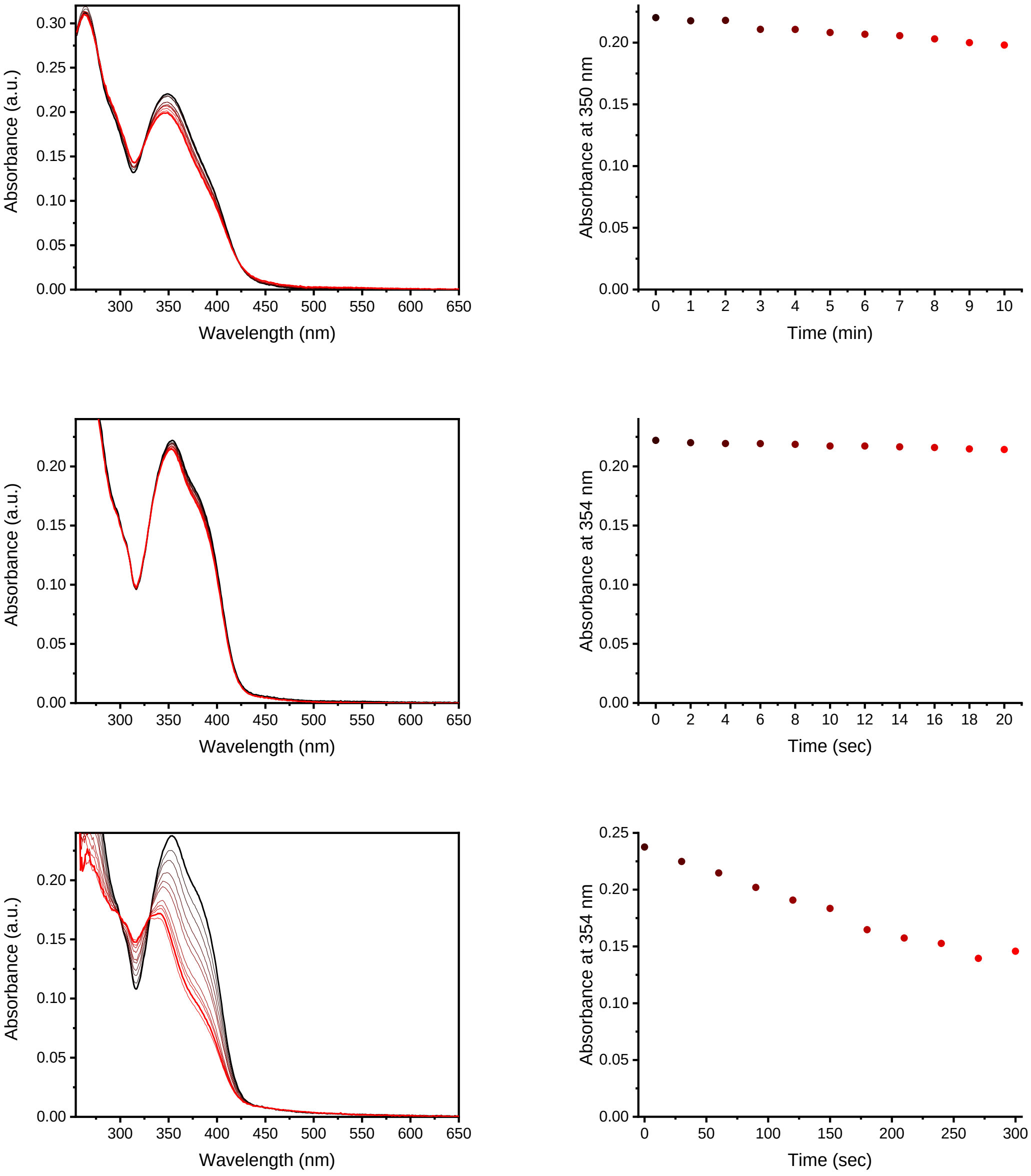

Fig. S6 (*top left*) Absorption spectra of **1** in aqueous solution before irradiation (black) and after irradiation at 405 nm (red, power = 27 mW/cm^2); (*top right*) Changes in absorbance at 350 nm upon irradiation at 405 nm (0–10 minutes); (*middle left*) Absorption spectra of **1** in DMSO before irradiation (black) and after irradiation at 405 nm (red, power = 24 mW/cm^2); (*middle right*) Changes in absorbance at 354 nm upon irradiation at 405 nm (0–20 seconds); (*bottom left*) Absorption spectra of **1** in DMSO before irradiation (black) and after irradiation at 405 nm (red, power = 24 mW/cm^2); (*bottom right*) Changes in absorbance at 354 nm upon irradiation at 405 nm (0–300 seconds).

In aqueous solution, photodecomposition of compound **1** is not observed until after irradiation at 405 nm for 3 minutes. Photodecomposition of compound **1** when irradiated at 405 nm appears to occur more rapidly in DMSO.

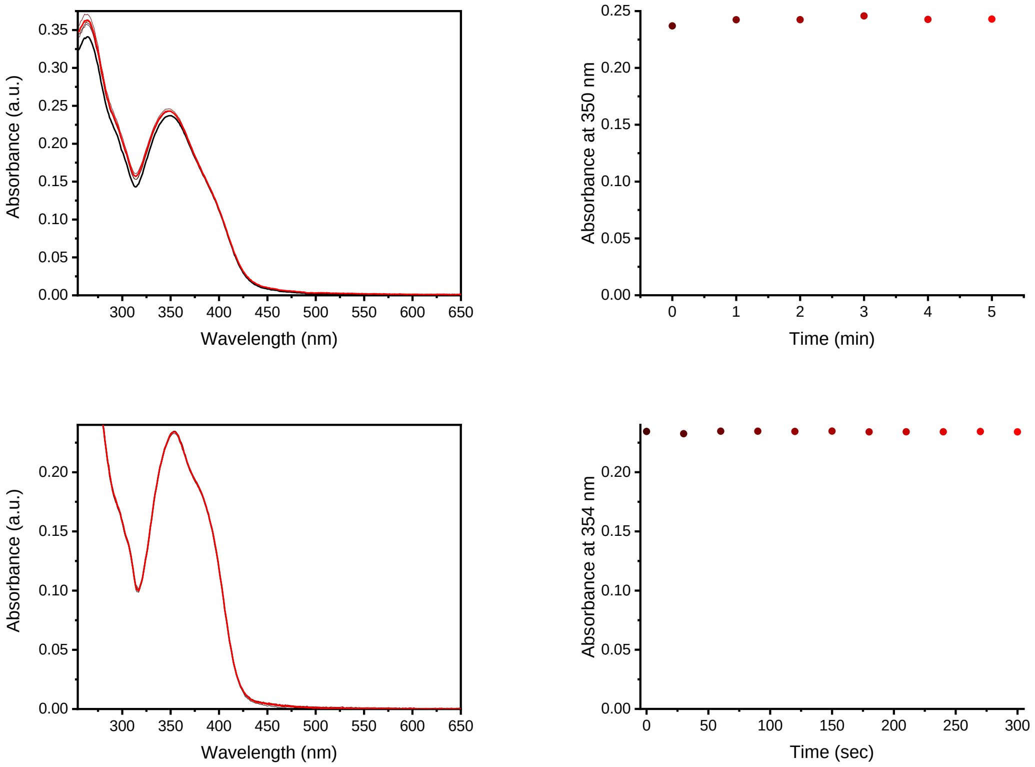

Fig. S7 (*top left*) Absorption spectra of **1** in aqueous solution before irradiation (black) and after irradiation at 523 nm (red, power = 6 mW/cm^2); (*top right*) Changes in absorbance at 350 nm upon irradiation at 523 nm (0–5 minutes); (*bottom left*) Absorption spectra of **1** in DMSO before irradiation (black) and after irradiation at 523 nm (red, power = 6 mW/cm^2); (*bottom right*) Changes in absorbance at 354 nm upon irradiation at 523 nm (0–300 seconds).

No photodecomposition of compound **1** is observed when irradiated at 523 nm. This result is expected, as compound **1** does not absorb light at this wavelength.

Thermal Stability of Compounds 1, 4a, 4b and 3c–e in Aqueous Solution (10% DMSO in H_2O) and DMSO.

Thermal stability of compound **1** and caged substrates were monitored by recording absorption spectra at regular intervals over the course of 24 hours at a concentration of *ca* 10 µM (unless stated otherwise). During this time, samples were kept in the dark at 21 °C.

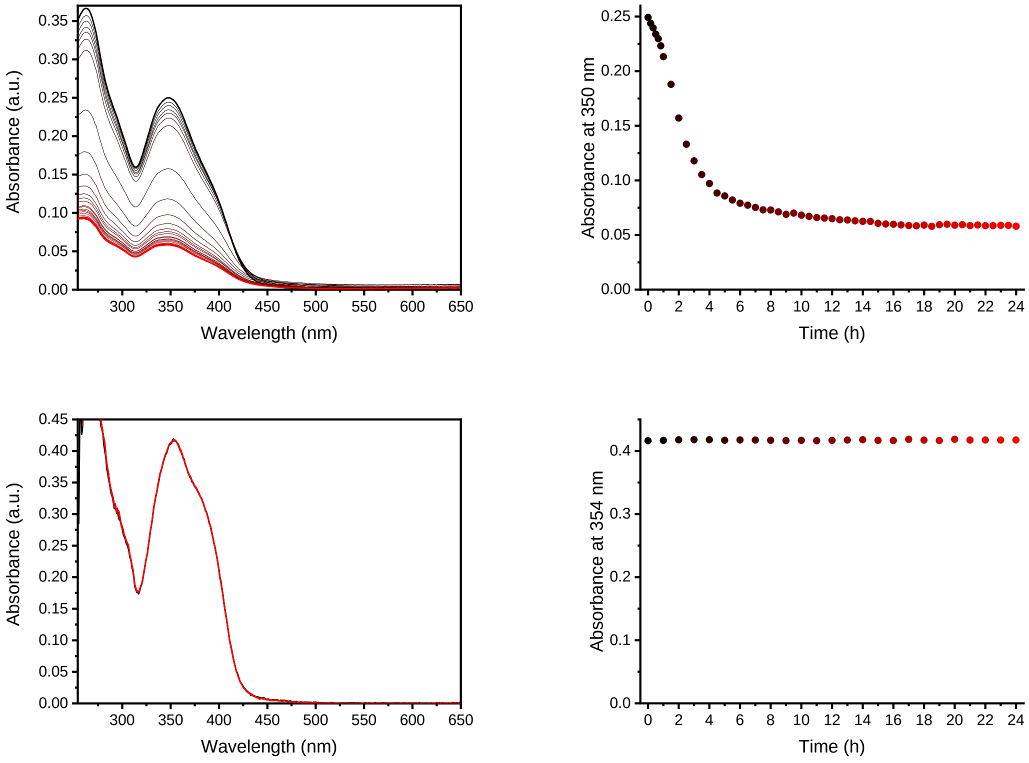

Fig. S8 (*top left*) Absorption spectra of compound **1** in aqueous solution (10% DMSO in H_2O) at t = 0 h (black) and after t = 24 h (red) at 21 °C; (*top right*) Absorbance at 350 nm as a function of time; (*bottom left*) Absorption spectra of compound **1** in DMSO (*ca* 15 µM) at t = 0 h (black) and after t = 24 h (red) at 21 °C; (*bottom right*) Absorbance at 354 nm as a function of time.

In aqueous solution, significant spectral changes are observed due to poor solubility of compound **1** under these conditions. No signs of degradation of compound **1** was detected in DMSO.

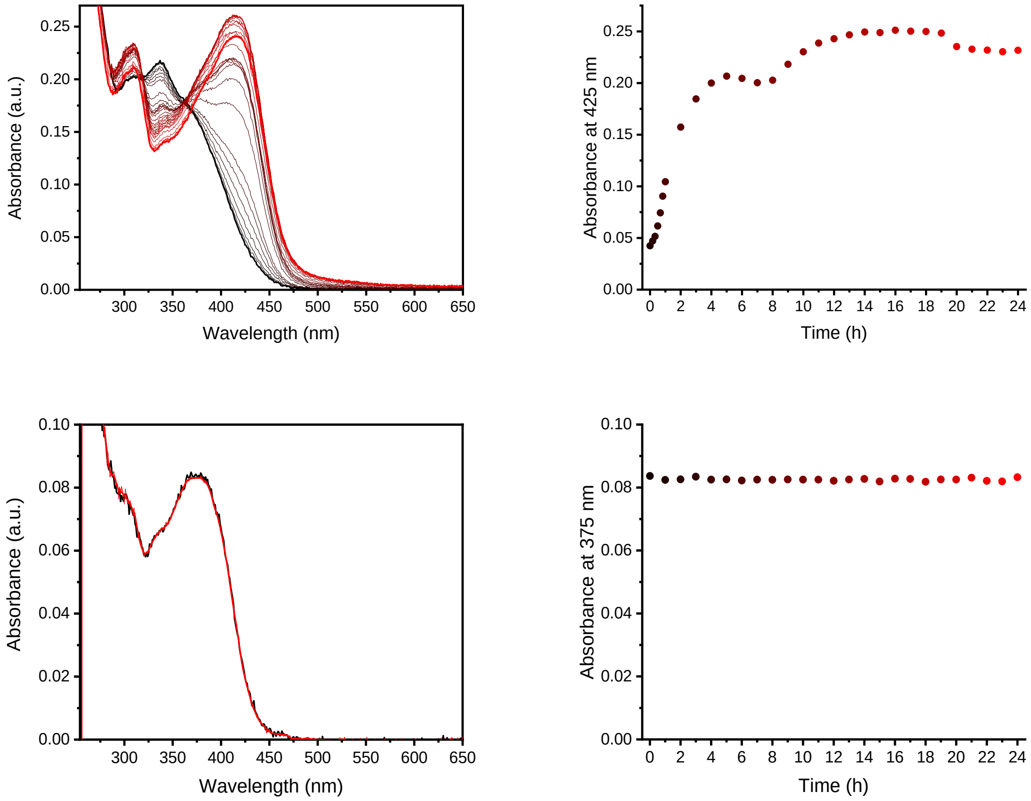

Fig. S9 (*top left*) Absorption spectra of compound **4a** in aqueous solution (10% DMSO in H_2O) at t = 0 h (black) and after t = 24 h (red) at 21 °C; (*top right*) Absorbance at 425 nm as a function of time; (*bottom left*) Absorption spectra of compound **4a** in DMSO (*ca* 3 µM) at t = 0 h (black) and after t = 24 h (red) at 21 °C; (*bottom right*) Absorbance at 375 nm as a function of time.

In aqueous solution, significant spectral changes are observed due to poor thermal stability of compound **4a** under these conditions. No signs of degradation of compound **4a** were detected in DMSO.

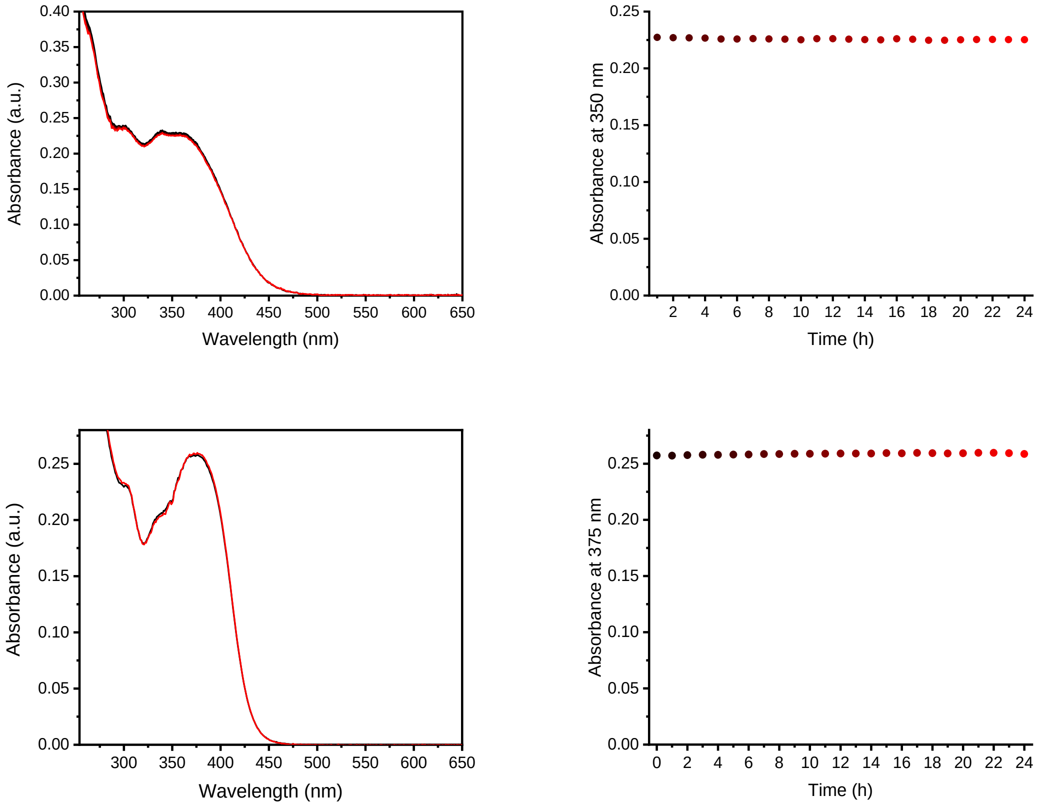

Fig. S10 (*top left*) Absorption spectra of compound **4b** in aqueous solution (10% DMSO in H_2O) at t = 0 h (black) and after t = 24 h (red) at 21 °C; (*top right*) Absorbance at 350 nm as a function of time; (*bottom left*) Absorption spectra of compound **4b** in DMSO at t = 0 h (black) and after t = 24 h (red) at 21 °C; (*bottom right*) Absorbance at 375 nm as a function of time.

No signs of degradation of compound **4b** were detected under the specified conditions.

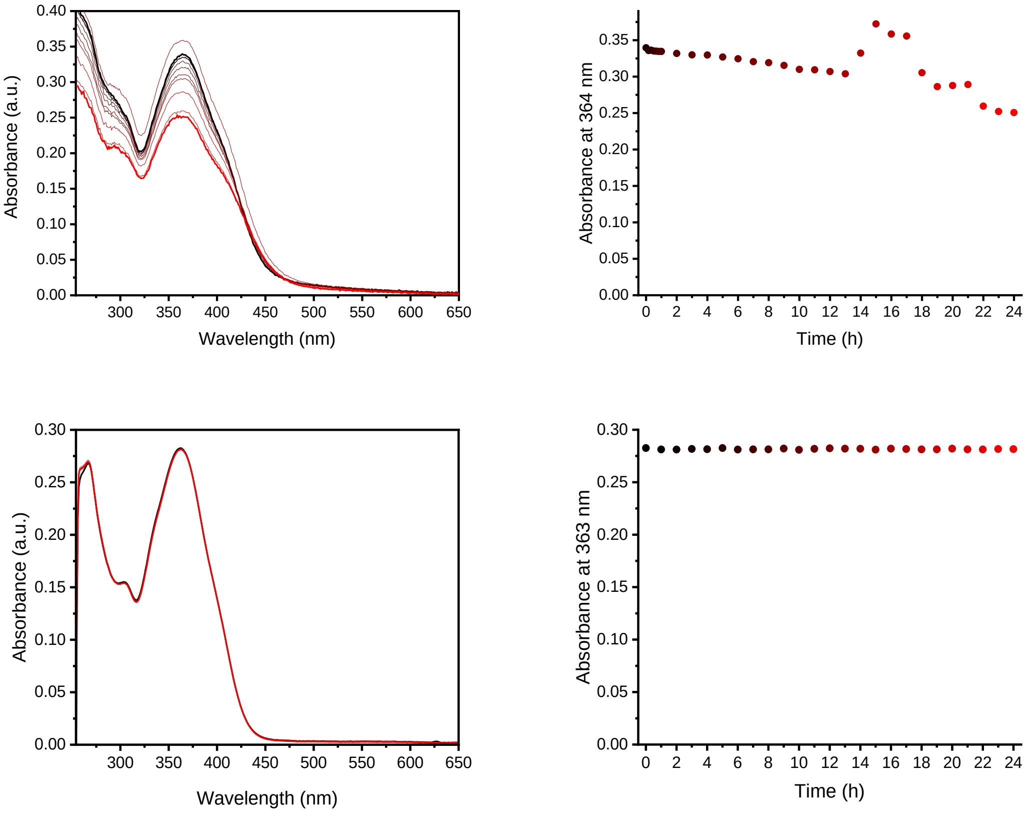

Fig. S11 (*top left*) Absorption spectra of compound **3c** in aqueous solution (10% DMSO in H_2O) at t = 0 h (black) and after t = 24 h (red) at 21 °C; (*top right*) Absorbance at 364 nm as a function of time; (*bottom left*) Absorption spectra of compound **3c** in DMSO at t = 0 h (black) and after t = 24 h (red) at 21 °C; (*bottom right*) Absorbance at 363 nm as a function of time.

In aqueous solution, significant spectral changes are observed due to poor solubility of compound **3c** under these conditions.

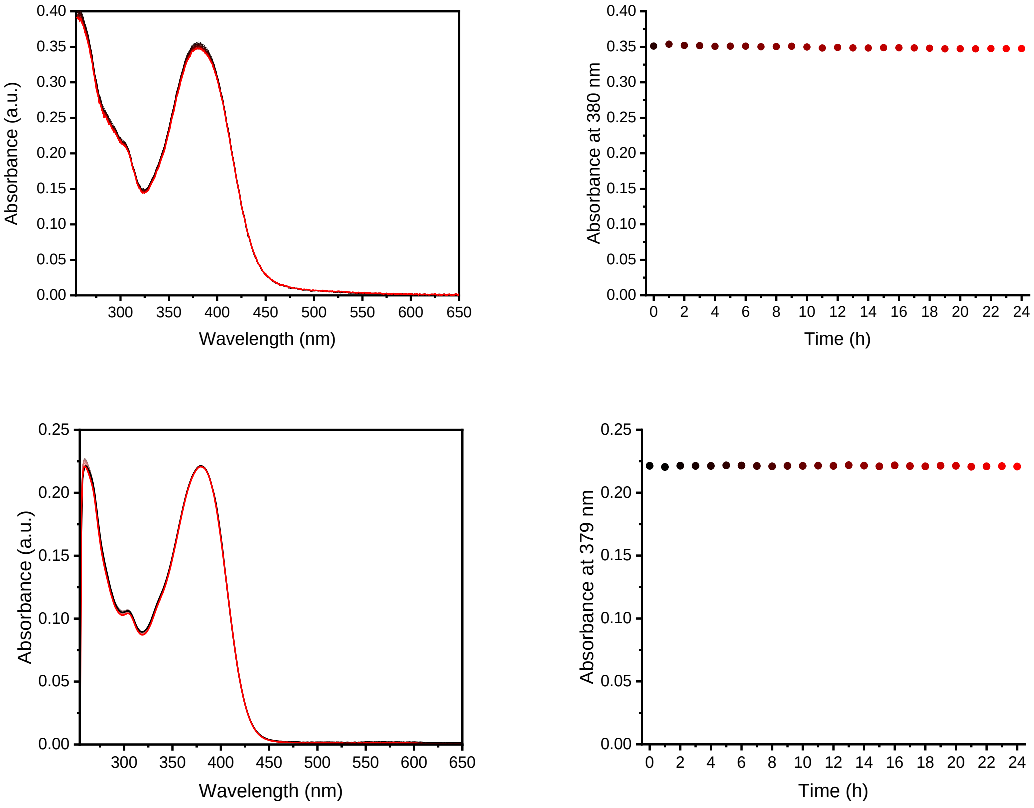

Fig. S12 (*top left*) Absorption spectra of compound **3d** in aqueous solution (10% DMSO in H_2O) at t = 0 h (black) and after t = 24 h (red) at 21 °C; (*top right*) Absorbance at 380 nm as a function of time; (*bottom left*) Absorption spectra of compound **3d** in DMSO (*ca* 5 µM) at t = 0 h (black) and after t = 24 h (red) at 21 °C; (*bottom right*) Absorbance at 379 nm as a function of time.

No signs of degradation of compound **3d** were detected under the specified conditions.

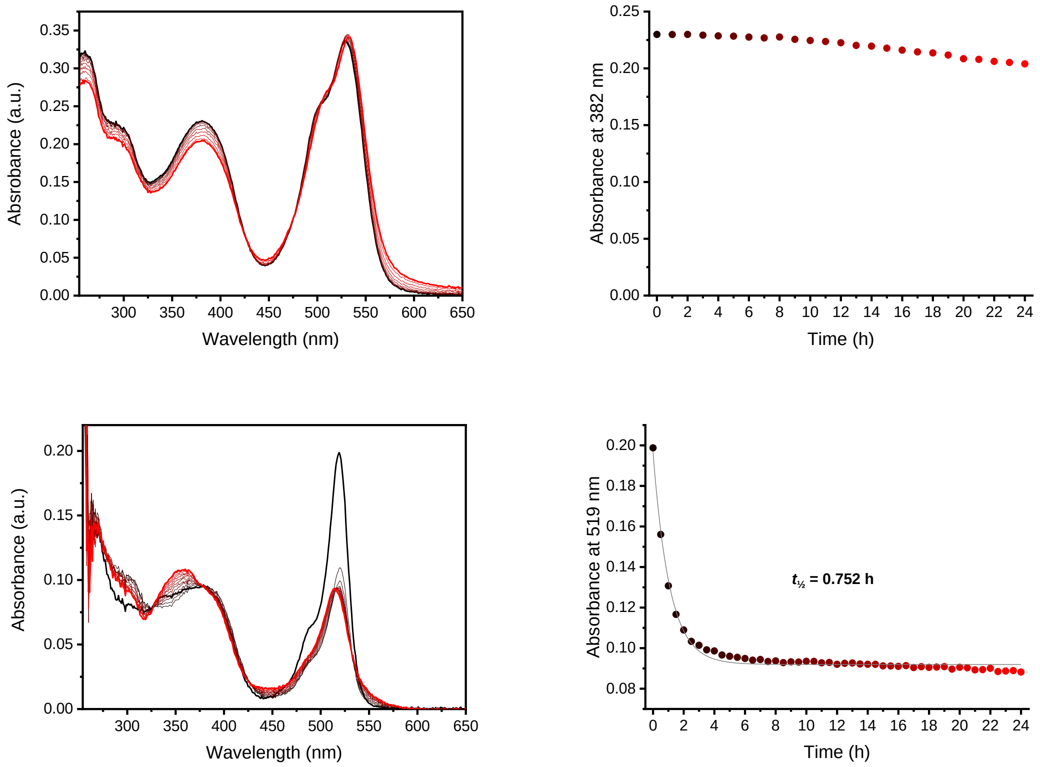

Fig. S13 (*top left*) Absorption spectra of compound **3e** in aqueous solution (*ca* 5 µM, 10% DMSO in H_2O) at t = 0 h (black) and after t = 24 h (red) at 21 °C; (*top right*) Absorbance at 382 nm as a function of time; (*bottom left*) Absorption spectra of compound **3e** in DMSO (*ca* 3 µM) at t = 0 h (black) and after t = 24 h (red) at 21 °C; (*bottom right*) Absorbance at 519 nm as a function of time.

In aqueous solution, small spectral changes are observed due to the solubility of compound **3e** under these conditions. Poor thermal stability of compound **3e** is observed in DMSO. Decaging occurs via thermal means with a half-life of 0.752 hours.

Absorption and Emission Spectra of Compounds 3c–e.

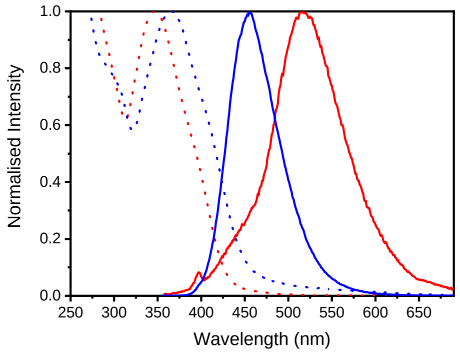

Fig. S14 Normalised absorption (dashed) and emission (solid) spectra of compound **1** (red) and compound **3c** (blue) in aqueous solution (10% DMSO in H_2O).

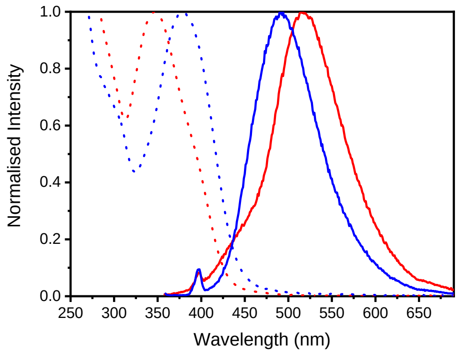

Fig. S15 Normalised absorption (dashed) and emission (solid) spectra of compound **1** (red) and compound **3d** (blue) in aqueous solution (10% DMSO in H_2O).

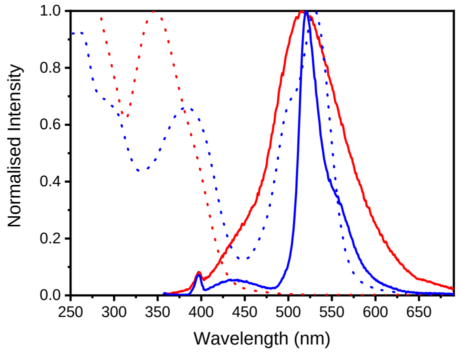

Fig. S16 Normalised absorption (dashed) and emission (solid) spectra of compound **1** (red) and compound **3e** (blue) in aqueous solution (10% DMSO in H_2O).

Spectroscopic Data for Decaged Products S12, S16 and S20.

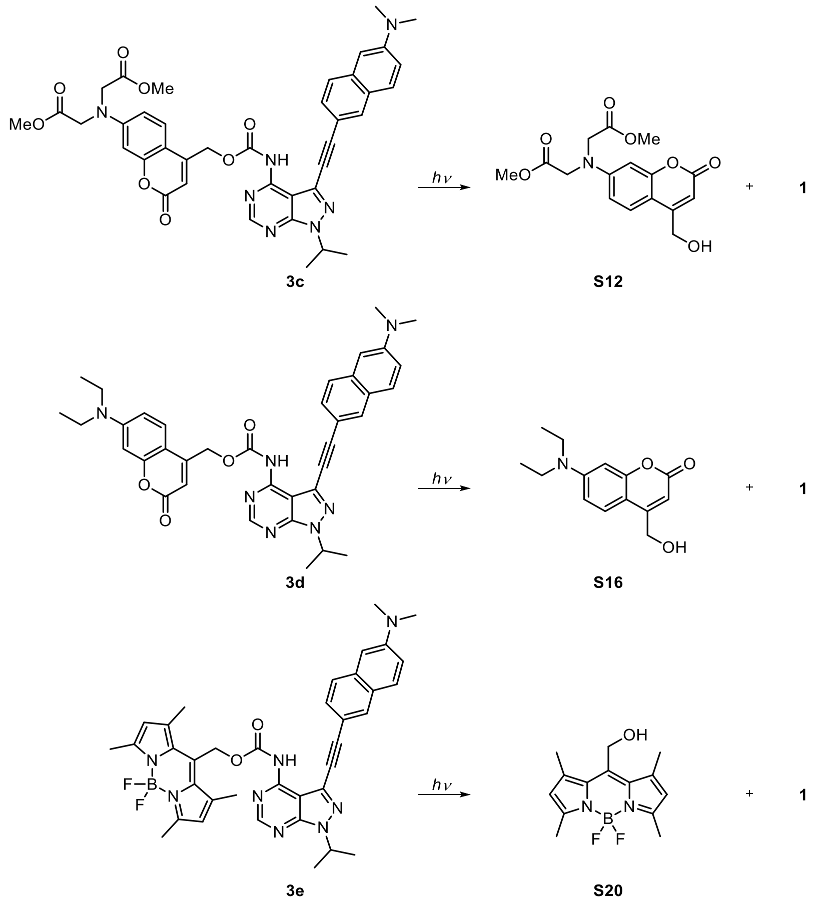

Fig. S17 Structure of decaged products formed during decaging reaction.

Table S1 Spectroscopic data for decaged products **S12**, **S16** and **S20** in aqueous solution (10% DMSO in H_2O).

Compound	λ_{abs} (nm)[a]	λ_{em} (nm)[b]	Stokes Shift (cm^{-1})	φ_F[c]
**S12**	355	453	6,094	0.97[d]
**S16**	387	491	5,473	0.07[e]
**S20**	518	530	437	0.81[f]

[a] Wavelength of absorption maximum. [b] Wavelength of emission maximum. [c] Fluorescence quantum yields were determined by taking 1,9-diphenylanthracene (DPA) in cyclohexane (ϕ_F = 0.97) as a reference. [d] Excitation wavelength at 350 nm. [e] Excitation wavelength at 385 nm. [f] Excitation wavelength at 490 nm.

Absorption and Emission Spectra of Compounds S12, S16 and S20.

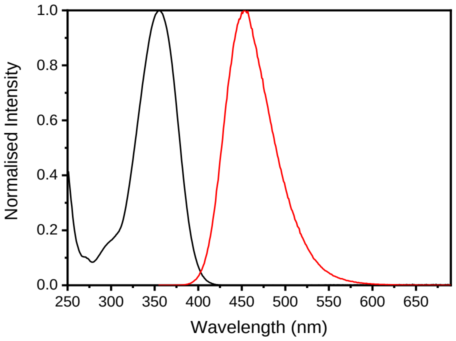

Fig. S18 Normalised absorption (black) and emission (red) spectra of decaged product **S12** in 10% DMSO/H_2O.

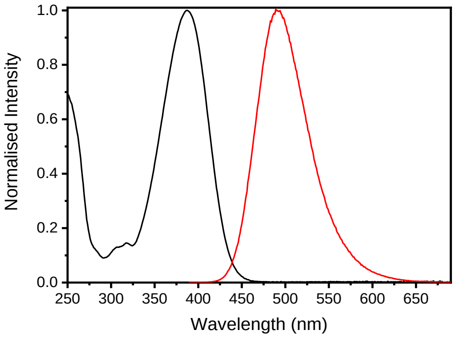

Fig. S19 Normalised absorption (black) and emission (red) spectra of decaged product **S16** in 10% DMSO/H_2O.

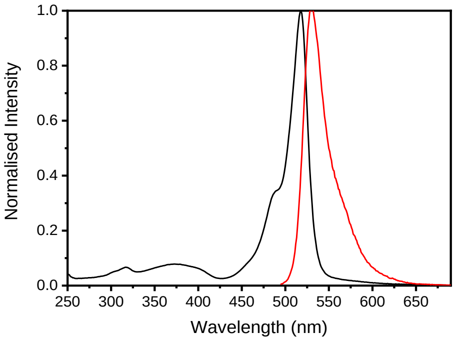

Fig. S20 Normalised absorption (black) and emission (red) spectra of decaged product **S20** in 10% DMSO/H_2O.

Cyclic Voltammetry.

To evaluate the possibility of photoinduced electron transfer (PET) in the dyads, we employed Rehm-Weller equation:

$$\Delta G = E_{ox} - E_{red} - E^* + C$$

where E_{ox} represents the oxidation potential of the fluorescent caging moiety, E_{red} is the reduction potential of the inhibitor, E^* is the excitation energy, and C is the Coulomb constant. The oxidation potentials for electron donors **S12**, **S16**, and **S20** were estimated in 0.93, 0.44, and 0.84 eV, respectively. The reduction potential for the inhibitor **1**, representing the electron acceptor, can be read as -0.44 eV. In relation to the excitation energy, this was determined in 3.40, 3.06 and 2.37 eV for dyads **3c–e**. According to these data, and taking the Coulomb constant as 0, the driving force for PET was approximated in $\Delta G \approx$ -2.03, -3.06 and -1.09 eV for dyads **3c–e**.

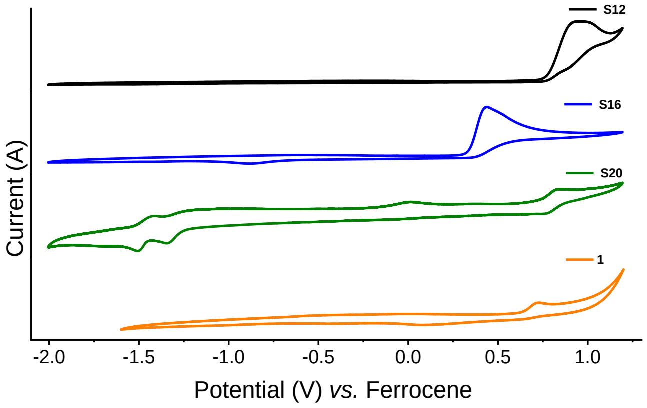

Fig. S21 Cyclic voltammograms showing oxidation and reduction of model compounds **S12** (black), **S16** (blue), **S20** (green) and **1** (orange). Measurements were collected in 0.10 M $N(n\text{-}Bu)_4PF_6$ ACN solution for **S12**, **S16** and **S20**, and in 0.10 M $N(n\text{-}Bu)_4PF_6$ DMSO solution for **1**. The voltammograms were collected at 0.20 V/s using a three-electrode system. Potentials are referenced to SCE *via* a ferrocene external standard.

Monitoring Decaging Reactions using Fluorescence Spectroscopy.

Decaging reactions were evaluated in aqueous solution (10% DMSO in H_2O). Samples were irradiated with LEDs at 365 nm, 405 nm or 523 nm depending on the absorption spectrum of the caged substrate. The reactions were monitored by recording emission spectra at regular intervals, employing 350 nm as the excitation wavelength.

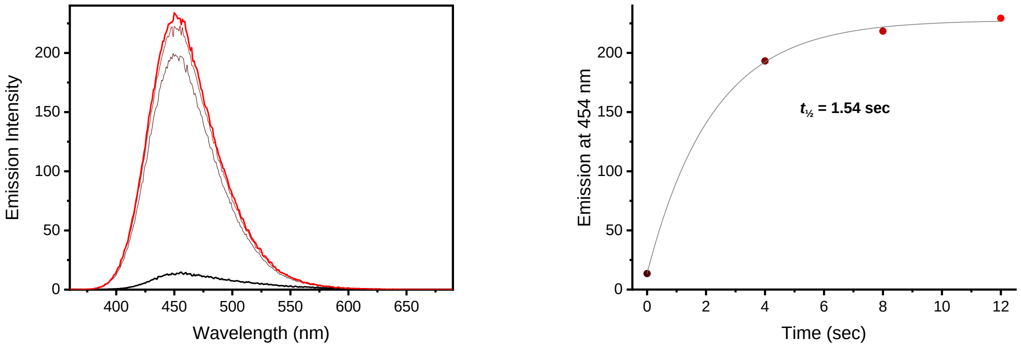

Fig. S22 (*left*) Emission spectra of **3c** in aqueous solution before irradiation (black) and after irradiation at 365 nm (red, power = 34 mW/cm^2), employing 350 nm as the excitation wavelength; (*right*) Changes in emission at 454 nm upon irradiation at 365 nm (0–12 seconds).

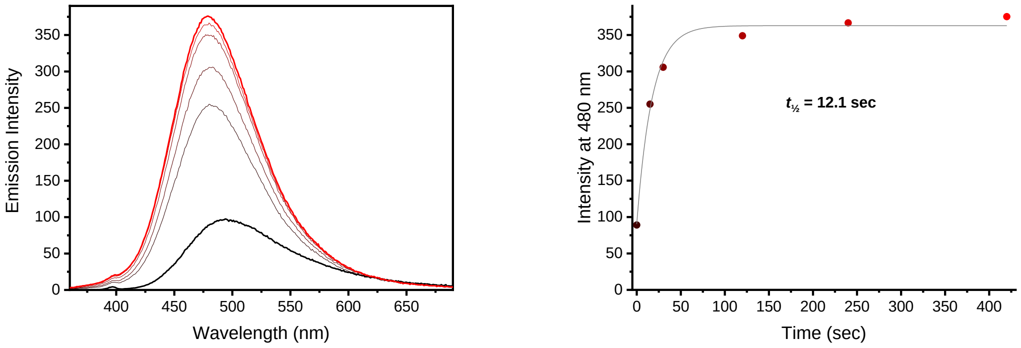

Fig. S23 (*left*) Emission spectra of **3d** in aqueous solution before irradiation (black) and after irradiation at 405 nm (red, power = 27 mW/cm^2), employing 350 nm as the excitation wavelength; (*right*) Changes in emission at 480 nm upon irradiation at 405 nm 0–420 seconds.

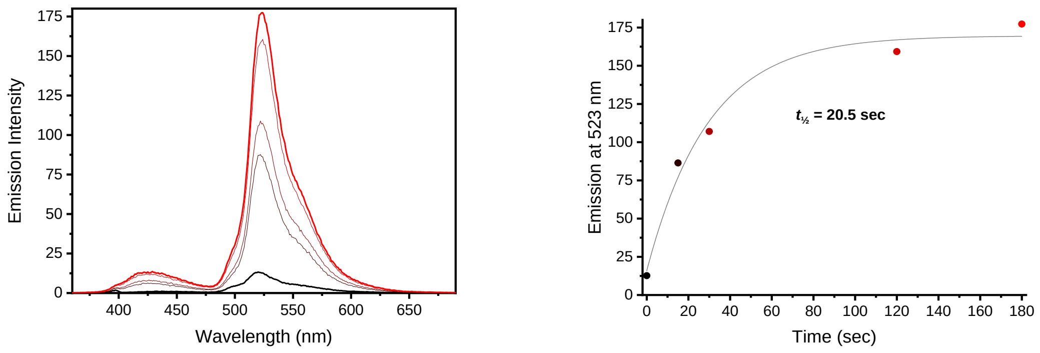

Fig. S24 (*left*) Emission spectra of **3e** in aqueous solution before irradiation (black) and after irradiation at 523 nm (red, power = 8 mW/cm^2), employing 350 nm as the excitation wavelength; (*right*) Changes in emission at 523 nm upon irradiation at 523 nm 0–180 seconds.

Monitoring Decaging Reactions using HPLC.

Decaging reactions were evaluated in aqueous solution (20% DMSO in H_2O). Samples were irradiated with LEDs at 365 nm, 405 nm or 523 nm depending on the absorption spectrum of the caged substrate. At regular intervals, an aliquot was drawn and analysed using HPLC. Note that data was not recorded for compound **3c** due to issues with solubility.

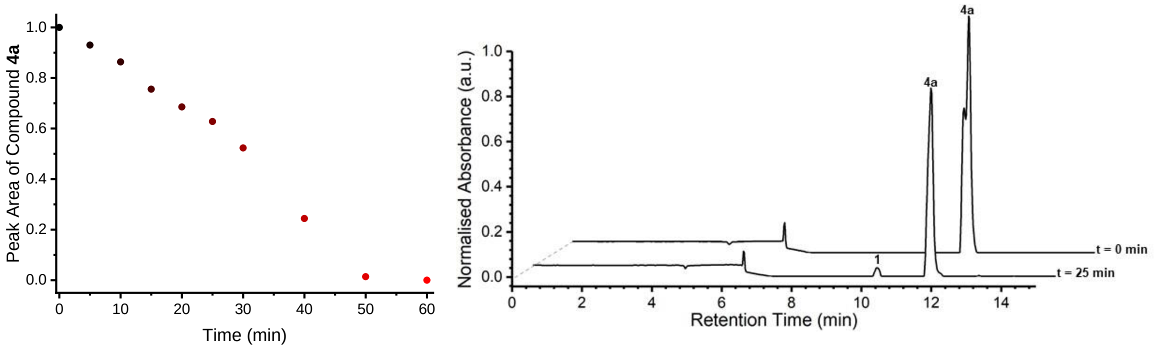

Fig. S25 Light-induced decaging of **4a**. (*left*) An aqueous solution (20% DMSO/H_2O) containing **4a** (100 μM) was successively subjected to t = 0, 5, 10, 15, 20, 25, 30, 40, 50 and 60 min of 365 LED light (power = 32 mW/cm^2). After each period, an aliquot was drawn and analysed using HPLC with UV detection (350 nm); (*right*) Overlaid HPLC traces (monitored at 350 nm) of an aqueous solution (20% DMSO/H_2O) containing **4a** (100 μM) before and after irradiation (365 LED light, power = 32 mW/cm^2) for 25 min.

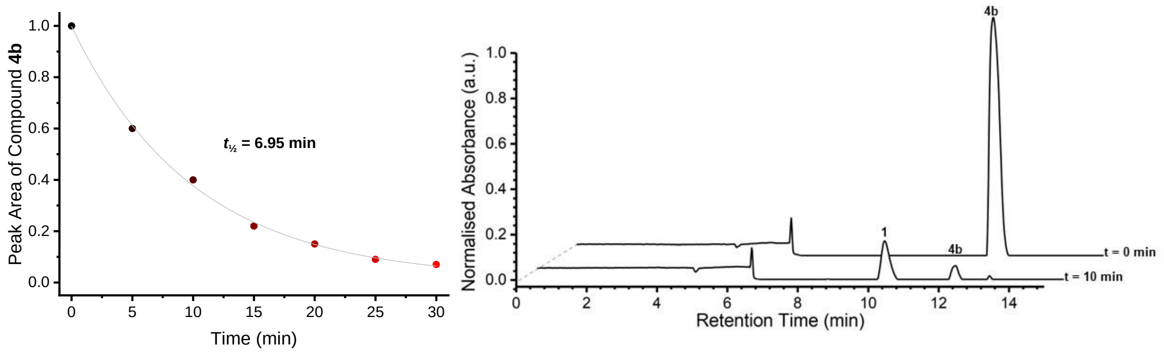

Fig. S26 Light-induced decaging of **4b**. (*left*) An aqueous solution (30% DMSO/H_2O) containing **4b** (100 μM) was successively subjected to t = 0, 5, 10, 15, 20, 25 and 30 min of 365 LED light (power = 32 mW/cm^2). After each period, an aliquot was drawn and analysed using HPLC with UV detection (350 nm); (*right*) Overlaid HPLC traces (monitored at 350 nm) of an aqueous solution (30% DMSO/H_2O) containing **4b** (100 μM) before and after irradiation (365 LED light, power = 32 mW/cm^2) for 10 min.

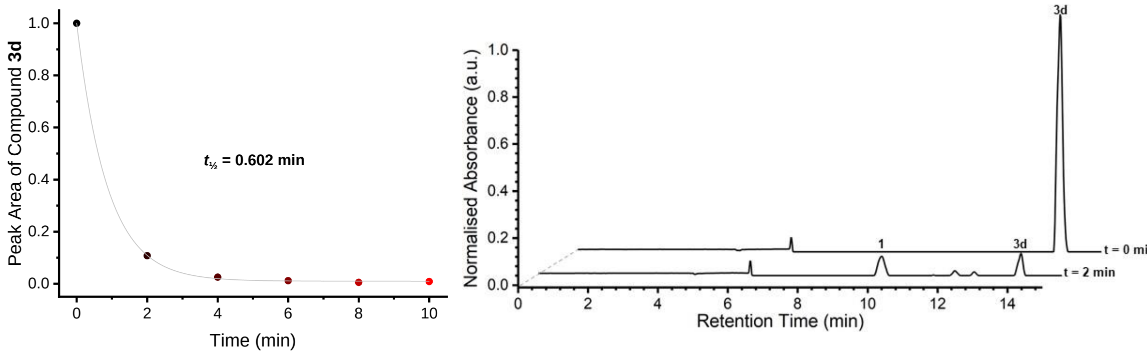

Fig. S27 Light-induced decaging of **3d**. (*left*) An aqueous solution (30% DMSO/H_2O) containing **3d** (100 μM) was successively subjected to t = 0, 2, 4, 6, 8 and 10 min of 405 LED light (power = 27 mW/cm^2). After each period, an aliquot was drawn and analysed using HPLC with UV detection (350 nm); (*right*) Overlaid HPLC traces (monitored at 350 nm) of an aqueous solution (30% DMSO/H_2O) containing **3d** (100 μM) before and after irradiation (405 LED light, power = 27 mW/cm^2) for 2 min.

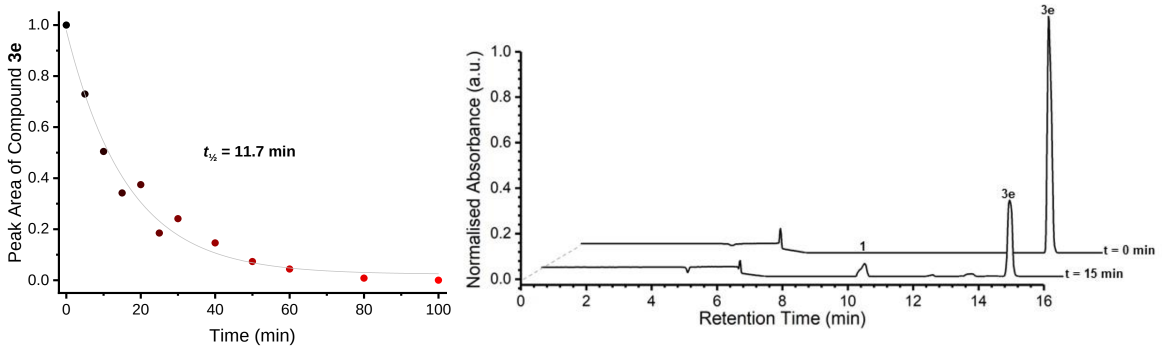

Fig. S28 Light-induced decaging of **3e**. (*left*) An aqueous solution (20% DMSO/H_2O) containing **3e** (100 μM) was successively subjected to t = 0, 5, 10, 15, 20, 25, 30, 40, 50, 60, 80 and 100 min of 523 LED light (power = 8 mW/cm^2). After each period, an aliquot was drawn and analysed using HPLC with UV detection (350 nm); (*right*) Overlaid HPLC traces (monitored at 350 nm) of an aqueous solution (20% DMSO/H_2O) containing **3e** (100 μM) before and after irradiation (523 LED light, power = 8 mW/cm^2) for 15 min.

IC_{50} Determination of LCK Inhibitor 1 and Caged Inhibitors 3c–e.

The IC_{50} values of LCK inhibitor (compound **1**) and caged LCK inhibitors (compounds **4a**, **4b** and **3c–e**) were determined using ADP-Glo^{TM} Kinase Assay by Promega. In this assay, the luminescence signal is proportional to the amount of ADP produced during the kinase reaction and is therefore proportional to kinase activity.

The caged compounds were dissolved in DMSO (5 mM) and then serially diluted (concentration gradients ranging from 5 mM to 30 nM). The ADP-Glo^{TM} Kinase Assay (Promega, V9101) for LCK (Promega, V2691) was carried out according to the manufacturer's protocol (Promega protocol TM313), with white 384-well low volume plates (Corning, Cat. # 3824), 5 µL total reaction volume, and in triplicates for each compound. Kinase reactions were run in kinase buffer (40 mM Tris (pH 7.5), 20 mM $MgCl_2$, 0.1 mg/mL BSA, 60 µM DTT, 2 mM $MnCl_2$) with 10 µM ATP, 10 ng LCK kinase, and 0.24 mg/mL substrate (Poly(Glu4,Tyr1)). All the serially diluted samples (in DMSO) were further diluted (1:10) by kinase buffer to prepare 5× inhibitor stock solutions. Then 1 µL of each 5× inhibitor stock solution (including DMSO control solution for each inhibitor) were added into 384-well plates, followed by addition of LCK kinase and the mixture was incubated at room temperature for 30 min. The kinase reaction was initiated by adding the LCK substrate working solution. The mixture was incubated at room temperature for 60 min. The reaction was terminated with 5µL of ADP-Glo™ Reagent and incubated at room temperature for 40 minutes. Then 10 µL Promega Kinase Detection reagent was added to each well and the mixture incubated at room temperature for another 40 min. The luminescence signal was recorded by a plate reader (Molecular Devices SpectraMax iD5, whole wavelength, integration time: 1 second, read from 1 mm above plate). Kinase activities were calculated in percent of DMSO control and plotted against the logarithm of inhibitor concentration. Data points were means of triplicates with standard deviations as error bars. Sigmoidal dose-response fitting (log_{10}(inhibitor) vs. Activity–variable slope) were performed by GraphPad Prism7.0 to give the IC_{50} values.

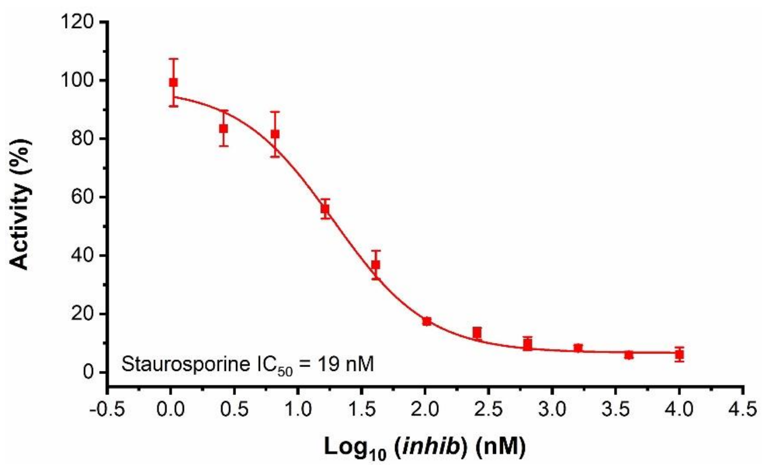

Fig. S29 IC_{50} data for Staurosporine against LCK.

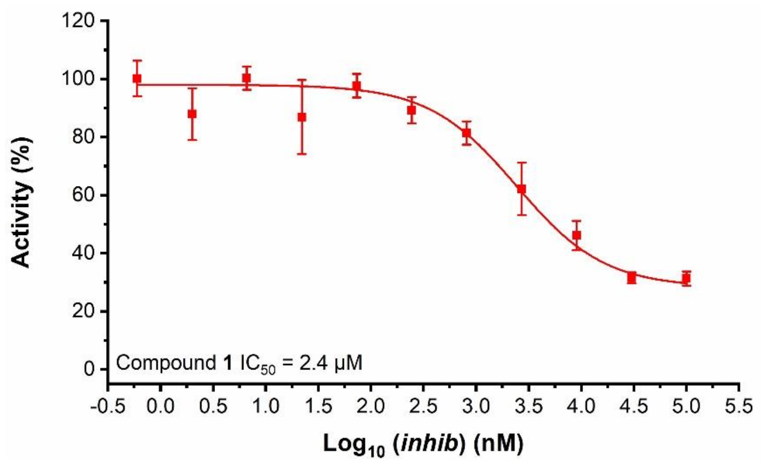

Fig. S30 IC_{50} data for compound **1** against LCK.

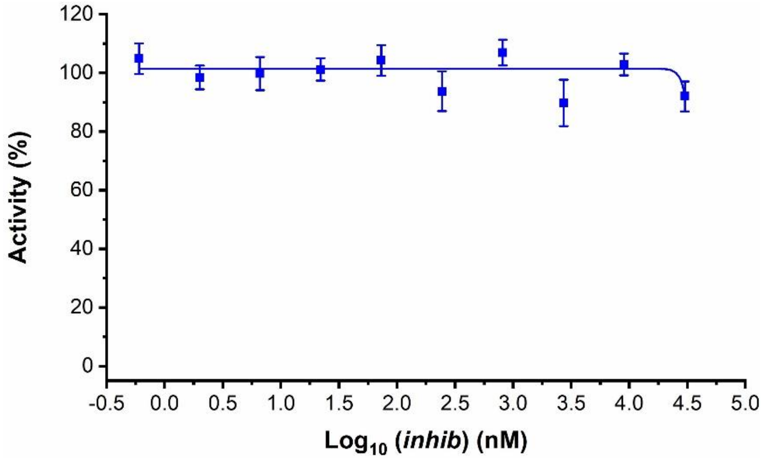

Fig. S31 IC_{50} data for compound **3c** against LCK.

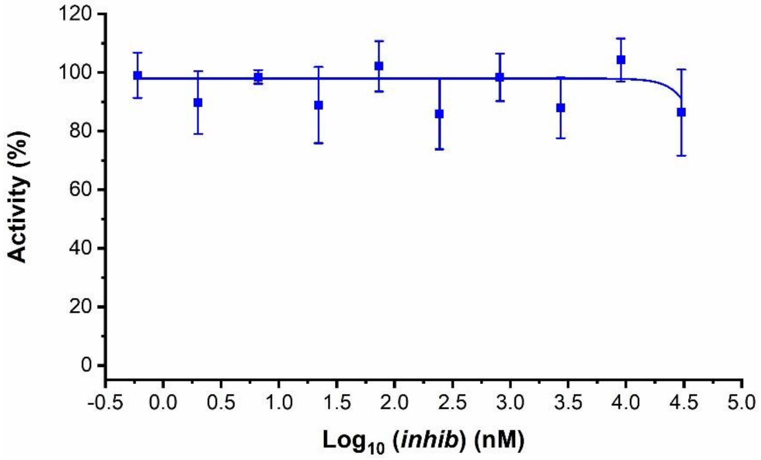

Fig. S32 IC_{50} data for compound **3d** against LCK.

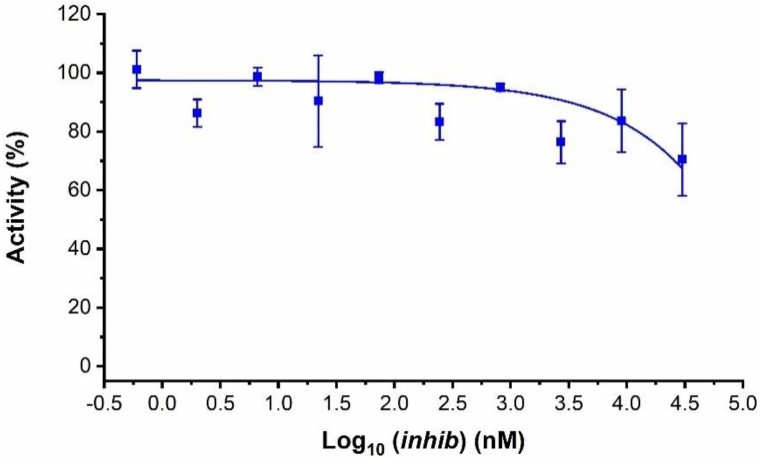

Fig. S33 IC_{50} data for compound **3e** against LCK.

Titration Experiments.

Titration experiments with the LCK protein were conducted by administering aliquots of a stock solution of compound **3e** to a reduced cuvette containing the protein. In order to eliminate the effects of dilution during titrations, the titrant solution also contained the protein (*i.e.* the host) at the same concentration as the receiving host solution. An emission spectrum was recorded 15 minutes after each addition.

Cellular Experiments.

Isolation of Cells from Peripheral Blood:

Peripheral blood mononuclear cells (PBMCs) were isolated from buffy coats. Samples were first set on 1% dextran for red blood cell sedimentation, after which mono- and polymorphonuclear cells were separated using density gradient centrifugation.[8] NK cells were isolated from PBMCs using NK isolation kit (Miltenyi Biotech, Bergisch Gladbach, Germany) according to the manufacturer's protocol.

Cell Lines:

Human erythroleukemia K562 cells (CCL-2243, ATCC, Virginia, USA) were modified using CRISPR-Cas9 genome editing to knock-out the expression of the three NK cell receptor ligands B7-H6, PVR and Nectin-2. The triple knock-out (tKO) K562 cells were maintained in Iscove's modified Dulbecco's medium (IMDM; Thermo Fisher Scientific) supplemented with 1% Penicillin/Streptomycin (Life Technologies), 10% fetal calf serum, 2mM L-glutamine and 1mM Na-pyruvate (all Gibco).

Cellular Uptake and Intracellular Decaging:

PBMCs were incubated with caged LCK inhibitor at 0.5, 1 or 5 µM for indicated time, after which cells were exposed to light at indicated wavelength and time using a LED-array or kept in the dark. Inhibitor uptake and decaging was measured using a 5-laser BD LSR Fortessa flow cytometer, where the LCK inhibitor was excited with a 355 nm UV-laser, and signal was detected at 425–475 and 500–530 nm.

Inhibition of Cellular Function:

NK cells activated overnight in IL-2 (500 IU/ml; PeproTech) were incubated with caged LCK inhibitors and either exposed to light using a LED-array (as specified for each compound) or kept in the dark. tKO K562 cells, stained with Cell Trace™ CFSE Cell proliferation kit (Invitrogen), were added to the NK cells, and cells were incubated in the presence of CD107a-PE antibody (BD Biosciences) for 3 h at

37 °C, 5% CO_2. The assay was performed in complete medium (RPMI supplemented with 10% FCS and 1% Penicillin/Streptavidin). Cells were stained with LIVE/DEAD® Fixable Near IR Dead stain kit (Invitrogen) and analyzed on a BD LSR Fortessa flow cytometer.

Statistics:

To analyze the impact of irradiation on NK cell degranulation and cytotoxicity, a two-way ANOVA was used, with irradiation or no irradiation and inhibitor concentrations as variables. Statistical significance was defined $p<0.05$. Statistical test was carried out with Graphpad Prism software.

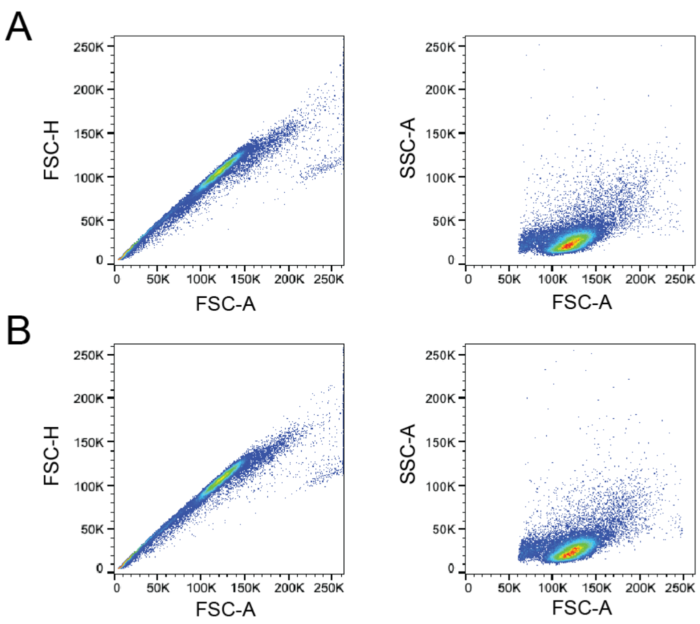

Fig. S34 Cellular morphology after exposure to caged LCK inhibitor **3c**. Flow cytometry data showing (A) forward scatter height *vs* area (*left*) and side scatter are *vs* forward scatter height (*right*) of PBMCs in the absence of caged inhibitor and; (B) forward scatter height *vs* area (*left*) and side scatter are *vs* forward scatter height (*right*) of PBMCs after treatment with caged inhibitor **3c** for 1 h.

Table S2 Wavelength and irradiation time used to decage compounds **3c–e** in cellular assays.

Compound	Decaging Wavelength	Time (sec)
**3c**	365 nm	100
**3d**	405 nm	100
**3e**	523 nm	350

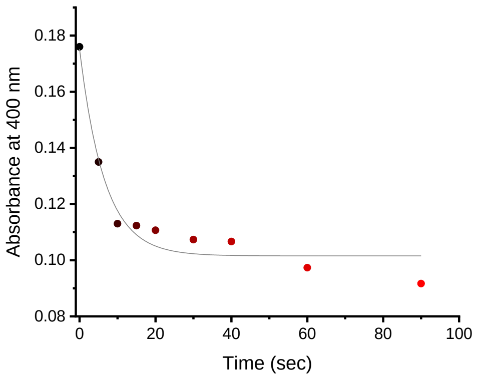

Fig. S35 Decaging of LCK inhibitor **3c** dissolved in cellular medium. Changes in absorbance at 400 nm upon irradiation at 365 nm (0–90 seconds).

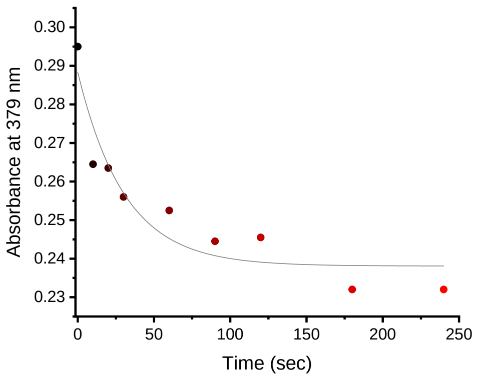

Fig. S36 Decaging of LCK inhibitor **3d** dissolved in cellular medium. Changes in absorbance at 379 nm upon irradiation at 405 nm (0–240 seconds).

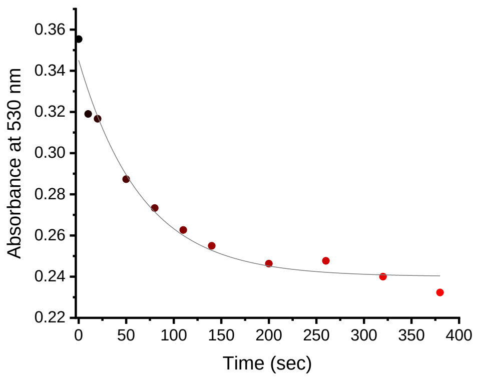

Fig. S37 Decaging of LCK inhibitor **3e** dissolved in cellular medium. Changes in absorbance at 530 nm upon irradiation at 523 nm (0–380 seconds).

Fluorescence Bioimaging Experiments.

Intracellular fluorescence and distribution following incubation with compounds **3c–e** in NK cells were analysed using an inverted Carl Zeiss LSM 880 Airyscan and a C-Apochromat 40×/1.2 W Corr FCS M27 objective. These compounds, as later specifically stated, were visualised upon excitation at different wavelengths (405 or 488 nm) in combination with appropriate filters (BP495-550+LP620 or BP420-480+LP605, according to their photophysical properties). Brightfield images were captured independently using 633 nm laser as light source. Colocalisation experiments were performed by adding CellMask^{TM} Deep Red (Thermofisher Scientific Cat. No. C10046; the used concentration was 0.5×, this prepared from 1000× purchased solution and according to the provider instructions). CellMask^{TM} Deep Red was sequentially visualised upon excitation at 633 nm. Optimal conditions were maintained throughout the imaging process with an integrated microscope enclosure keeping cells at 37 °C and 5% CO_2.

NK cells were isolated from human healthy donors as indicated in the previous section, and then kept in complete medium (RPMI + 10% FCS + 1% PEST) at 37 °C in a humidified environment with 5% CO_2. For microscopy experiments, NK cells were transferred to a 96-well plate and different conditions were applied, as explained in the step-by-step procedures prior to the bioimaging. Image processing was done by using ZEN software from Zeiss, and co-localisation analysis was performed with ImageJ with JACoP toolbox.[9]

NK Cells Irradiated with Light

NK cells were first incubated with **3e-c** (final concentration :5 µM) in complete medium, washed by centrifugation (350×g, 3 min, 4 °C), resuspended in E-buffer, and irradiated with different lights and time according to Table 2. After the exposition to light, NK cells were stained with CellMask^{TM} Deep Red as stated before, newly washed by centrifugation, resuspended in E-buffer, and taken to glass-bottomed micro-Insert 4 Well in µ-dish 35 mm dishes (Ibidi) suitable for optical microscopy.

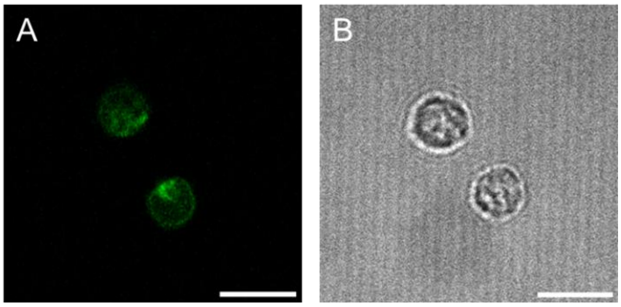

Fig. S38 (A) Fluorescence image of NK cells treated with **3c** upon exposure to 405 nm irradiation (green, λ_{exc} = 405 nm; see above for more details on light treatment before bioimaging), and (B) corresponding brightfield image. Scale bar: 10 µm.

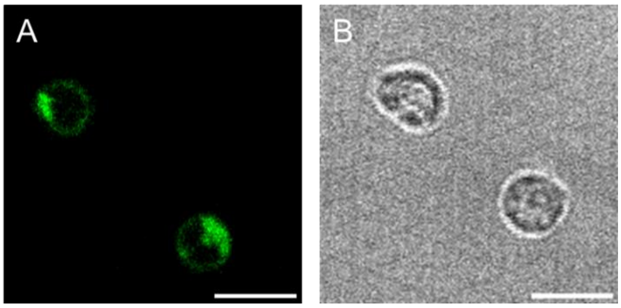

Fig. S39 (A) Fluorescence image of NK cells treated with **3d** upon exposure to 405 nm irradiation (green, λ_{exc} = 405 nm; see above for more details on light treatment before bioimaging), and (B) corresponding brightfield image. Scale bar: 10 µm.

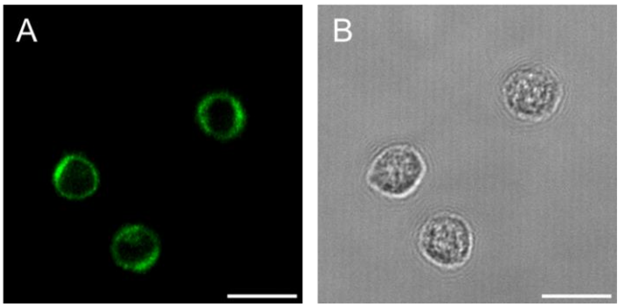

Fig. S40 (A) Fluorescence image of NK cells treated with **3e** upon exposure to 523 nm irradiation (green, λ_{exc} = 532 nm), and (B) corresponding brightfield image. Scale bar: 10 µm.

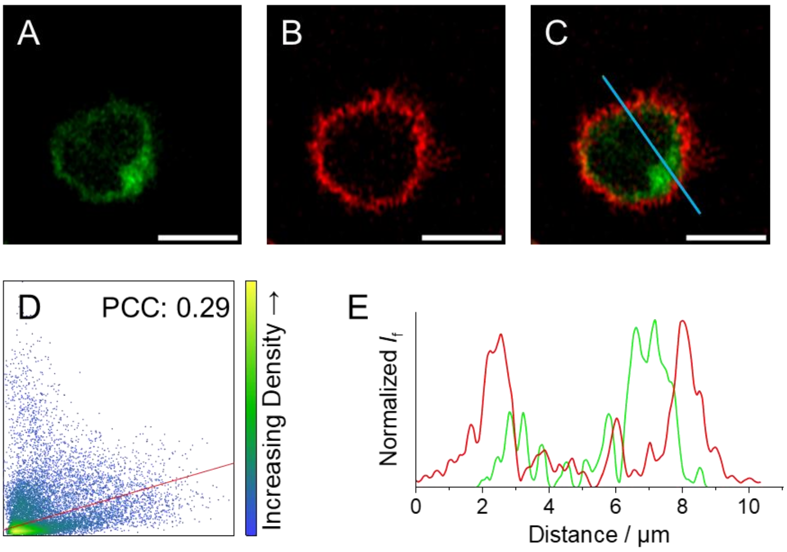

Fig. S41 Fluorescence images of NK cells incubated with (A) **3c** upon exposure to 365 nm light (green, λ_{exc} = 405 nm; see above for more details on light treatment before bioimaging) and (B) CellMask^{TM} Deep Red (red, λ_{exc} = 633 nm). (C) Colocalisation-merged images of **3c** and CellMask^{TM} Deep Red. (D) Correlation plot and PCC for **3c** and CellMask^{TM} Deep Red images. (E) Emission intensity profile plots along the blue line for the different compounds under study. Scale bar: 5 µm.

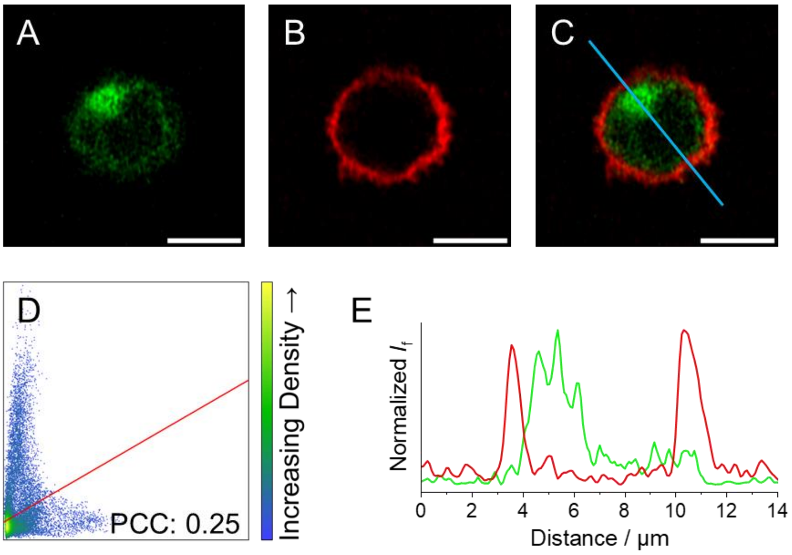

Fig. S42 Fluorescence images of NK cells incubated with (A) **3d** upon exposure to 405 nm light (green, λ_{exc} = 405 nm; see above for more details on light treatment before bioimaging) and (B) CellMask^{TM} Deep Red (red, λ_{exc} = 633 nm). (C) Colocalisation-merged images of **3d** and CellMask^{TM} Deep Red. (D) Correlation plot and PCC for **3d** and CellMask^{TM} Deep Red images. (E) Emission intensity profile plots along the blue line for the different compounds under study. Scale bar: 5 µm.

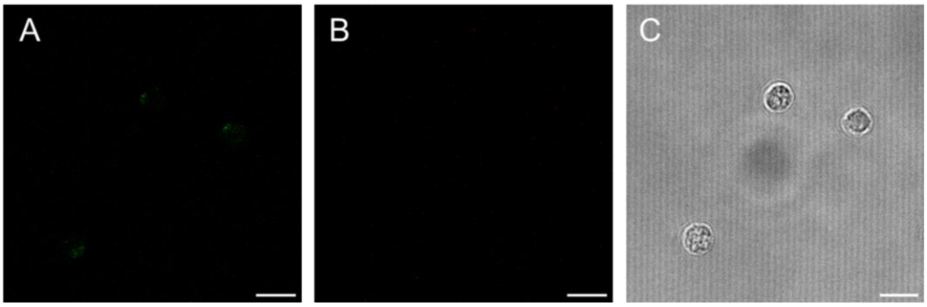

Fig. S43 Fluorescence images of control NK cells after irradiation with 405 nm light and without addition of any compound under (A) excitation at 405 nm (conditions optimised for visualisation of **3c**), and (B) excitation at 633 nm. (C) Corresponding brightfield image. Scale bar: 10 µm. *Note that different 405 nm laser powers were employed for the visualisation of compounds **3c** and **3d**.*

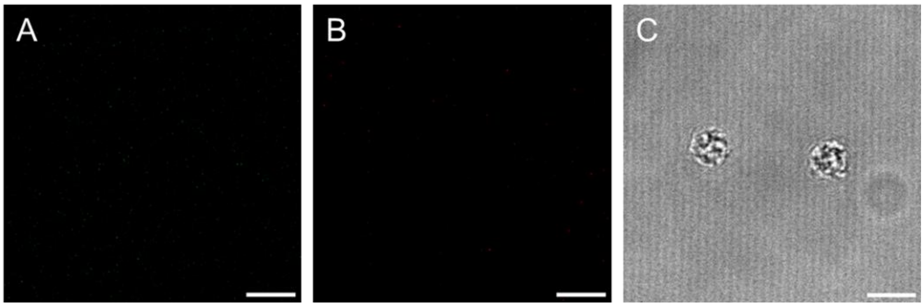

Fig. S44 Fluorescence images of control NK cells after irradiation with 405 nm light and without addition of any compound under (A) excitation at 405 nm (conditions optimised for visualisation of **3d**), and (B) excitation at 633 nm. (C) Corresponding brightfield image. Scale bar: 10 µm. *Note that different 405 nm laser powers were employed for the visualisation of compounds **3c** and **3d**.*

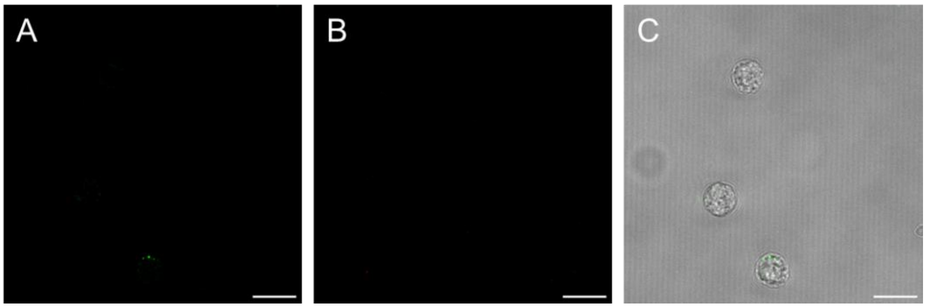

Fig. S45 Fluorescence images of control NK cells after irradiation with 523 nm light and without addition of any compound under (A) excitation at 523 nm, and (B) excitation at 633 nm. (C) Corresponding brightfield image. Scale bar: 10 µm.

NK Cells Not Irradiated with Light

NK cells were first incubated with **3e-c** (final concentration: 5 µM) in complete medium, washed by centrifugation (350×g, 3 min, 4 °C), and resuspended in E-buffer. Later, NK cells were stained with CellMask^{TM} Deep Red as stated before, newly washed by centrifugation, resuspended in E-buffer, and taken to glass-bottomed micro-Insert 4 Well in µ-dish 35 mm dishes (Ibidi) suitable for optical microscopy.

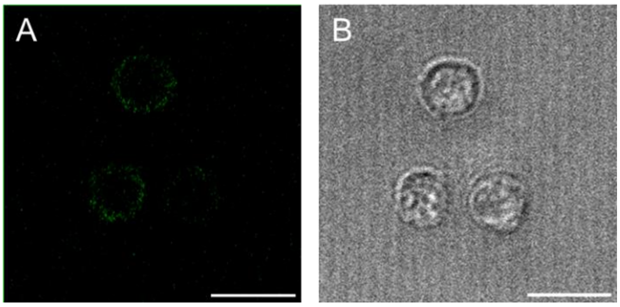

Fig. S46 (A) Fluorescence image of NK cells treated with **3c** (green, λ_{exc} = 405 nm), and (B) corresponding brightfield image. Scale bar: 10 µm.

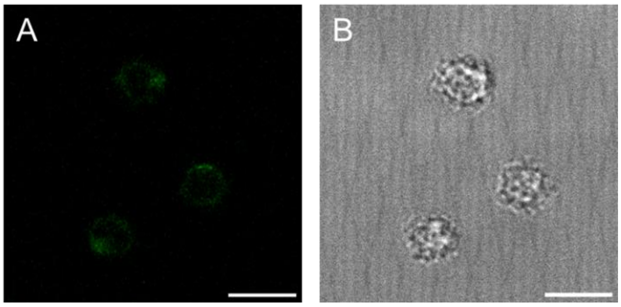

Fig. S47 (A) Fluorescence image of NK cells treated with **3d** (green, λ_{exc} = 405 nm), and (B) corresponding brightfield image. Scale bar: 10 µm.

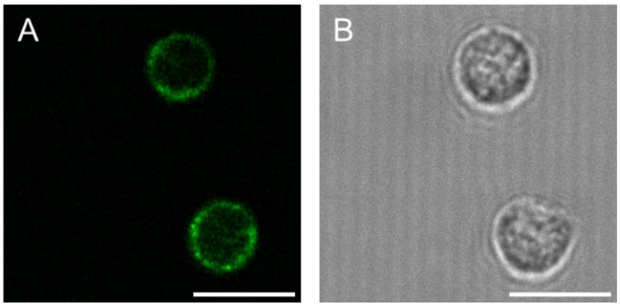

Fig. S48 (A) Fluorescence image of NK cells treated with **3e** (green, λ_{exc} = 532 nm), and (B) corresponding brightfield image. Scale bar: 10 µm.

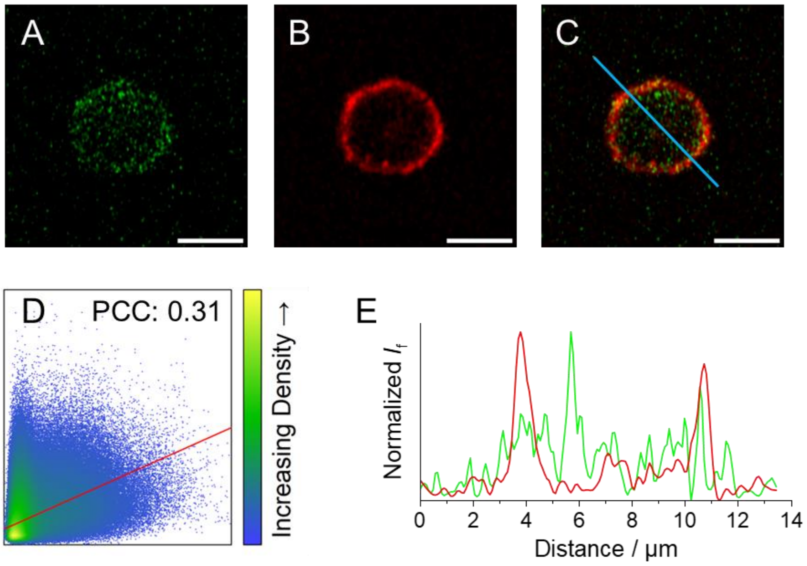

Fig. S49 Fluorescence images of NK cells incubated with (A) **3c** (green, λ_{exc} = 405 nm) and (B) CellMask™ Deep Red (red, λ_{exc} = 633 nm). (C) Colocalisation-merged images of **3c** and CellMask™ Deep Red. (D) Correlation plot and PCC for **3c** and CellMask™ Deep Red images. (E) Emission intensity profile plots along the blue line for the different compounds under study. Scale bar: 5 µm. *Note that **3c** fluorescence intensity is very low in this case.*

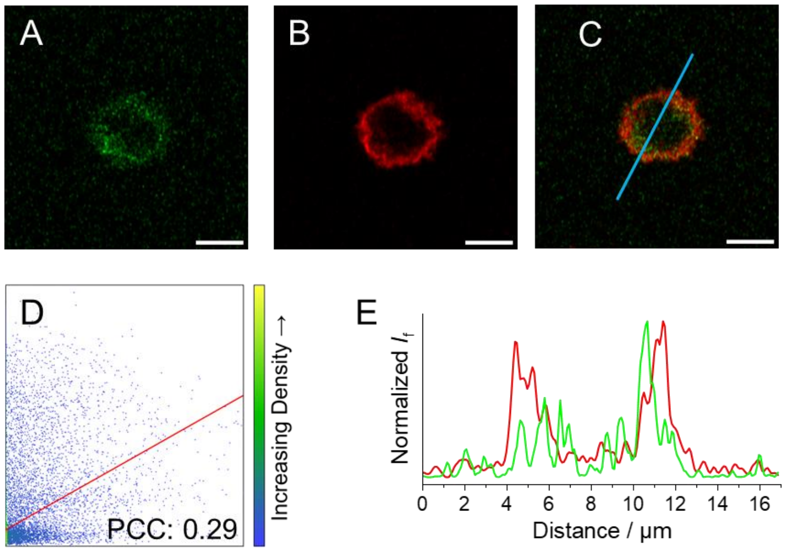

Fig. S50 Fluorescence images of NK cells incubated with (A) **3d** (green, λ_{exc} = 405 nm) and (B) CellMask^{TM} Deep Red (red, λ_{exc} = 633 nm). (C) Colocalisation-merged images of **3d** and CellMask^{TM} Deep Red. (D) Correlation plot and PCC for **3d** and CellMask^{TM} Deep Red images. (E) Emission intensity profile plots along the blue line for the different compounds under study. Scale bar: 5 µm. *Note that **3d** fluorescence intensity is very low in this case. Then, there is an apparent coincidence in the line profiles between the signals. However, the visual inspection of (C) and the PCC indicate there is no such agreement.*

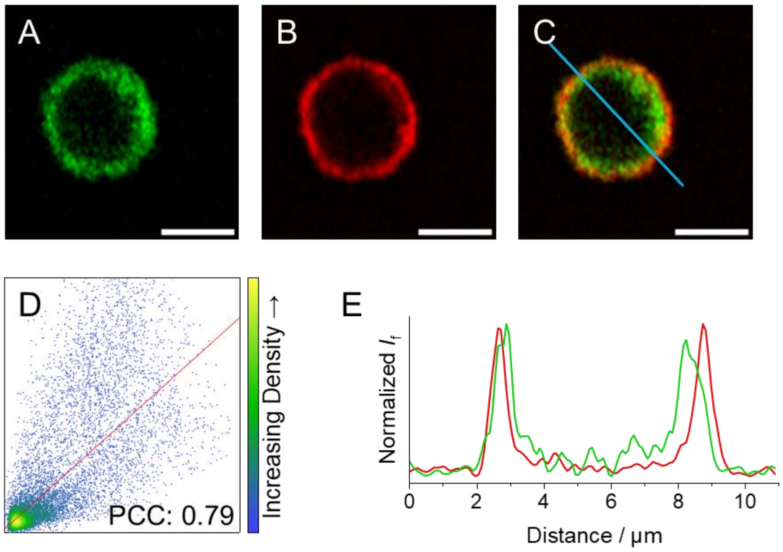

Fig. S51 Fluorescence images of NK cells incubated with (A) **3e** (green, λ_{exc} = 532 nm) and (B) CellMask^{TM} Deep Red (red, λ_{exc} = 633 nm). (C) Colocalisation-merged images of **3e** and CellMask^{TM} Deep Red. (D) Correlation plot and PCC for **3e** and CellMask^{TM} Deep Red images. (E) Emission intensity profile plots along the blue line for the different compounds under study. Scale bar: 5 µm.

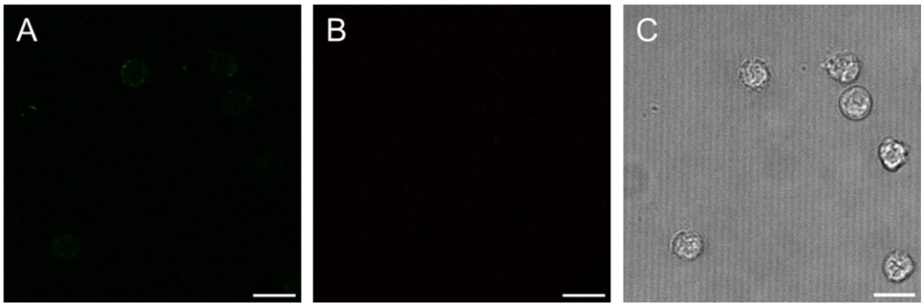

Fig. S52 Fluorescence images of control NK cells without addition of any compound under (A) excitation at 405 nm (conditions optimised for visualisation of **3c**), and (B) excitation at 633 nm. (C) Corresponding brightfield image. Scale bar: 10 µm. *Note that different 405 nm laser powers were employed for the visualisation of compounds **3c** and **3d**.*

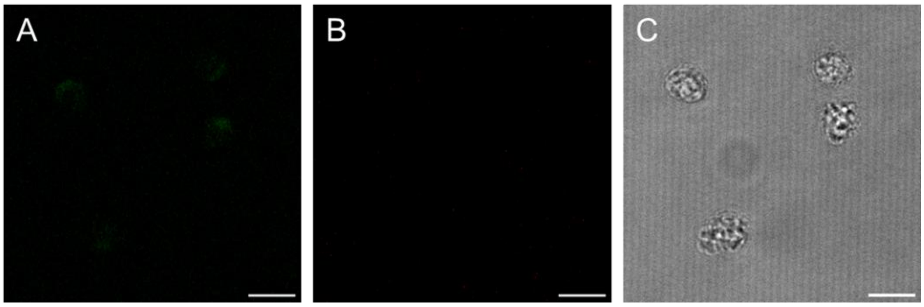

Fig. S53 Fluorescence images of control NK cells without addition of any compound under (A) excitation at 405 nm (conditions optimised for visualisation of **3d**), and (B) excitation at 633 nm. (C) Corresponding brightfield image. Scale bar: 10 µm. *Note that different 405 nm laser powers were employed for the visualisation of compounds **3c** and **3d**.*

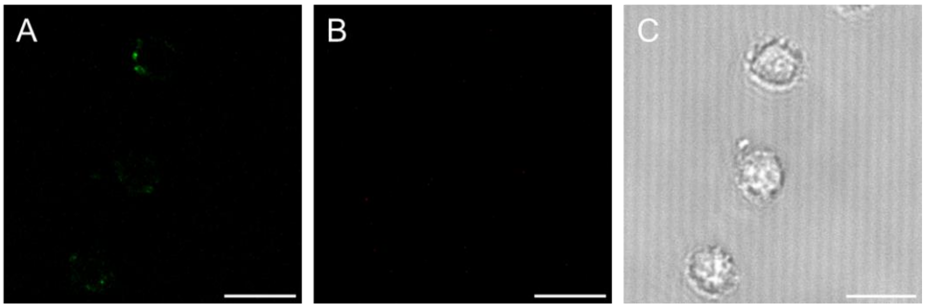

Fig. S54 Fluorescence images of control NK cells without addition of any compound under (A) excitation at 523 nm, and (B) excitation at 633 nm. (C) Corresponding brightfield image. Scale bar: 10 µm.

Corrected Total Cell Fluorescence (CTCF) was calculated by using ImageJ[9] and according to the next equation:

$$CTCF = Integrated\ Density - (Area\ of\ selected\ cell \times Mean\ fluorescence\ of\ background\ readings)$$

For the calculation of CTCF, three-dimensional images or Z-stacks were reduced to bidimensional images by arithmetic sum of the fluorescence intensity of the different slices. CTCF was expressed as the average of measuring various cells. As for the background readings, equal number of ROIs were drawn in the corresponding pictures. To give a clearer vision of the light impact on the photocleaving process, the intensity was normalised to 1 for each case according to the fluorescence observed in cells that were kept in the dark. This allows one to observe how efficient was the release by light treatment for each caged compound.

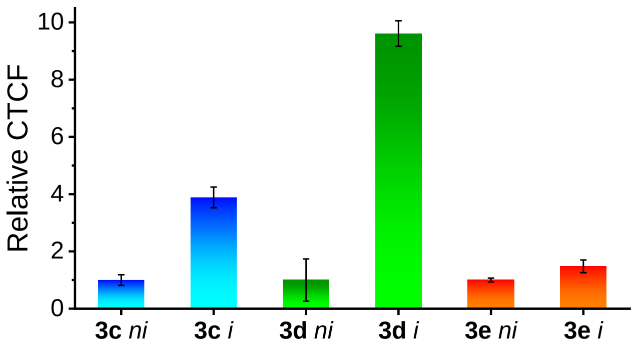

Fig. S55 Relative CTCF analysis for each compound under the different exposed conditions. *ni* and *i* indicate if cells were not irradiated or irradiated with light in the presence of the different compounds, respectively.

References

[1] C. L. Fleming, P. A. Sandoz, T. Inghardt, B. Önfelt, M. Grøtli and J. Andréasson, *Angew. Chem. Int. Ed.*, 2019, **58**, 15000–15004.

[2] K. Wada, J.-Y. Lee, H.-Y. Hung, Q. Shi, L. Lin, Y. Zhao, M. Goto, P.-C. Yang, S.-C. Kuo, H.-W. Chen and K.-H. Lee, *Bioorg. Med. Chem.*, 2015, **23**, 1507–1514.

[3] T. Weinrich, M. Gränz, C. Grünewald, T. F. Prinser and M. W. Göbel, *Eur. J. Org. Chem.*, 2017, 491–496.

[4] P. T. Wong, E. W. Roberts, S. Tang, J. Mukherjee, J. Cannon, A. J. Nip, K. Corbin, M. F. Krummel and S. K. Choi, *ACS Chem. Biol.*, 2017, **12**, 1001–1010.

[5] a) K. Krumova and G. Cosa, *J. Am. Chem. Soc.*, 2010, **132**, 17560–17569; b) K. Sitkowska, B. L. Feringa and W. Szymański, *J. Org. Chem.*, 2018, **83**, 1819–1827.

[6] L. Casimiro, L. Andreoni, J. Groppi, A. Credi, R. Métivier and S. Silvi, *Photochem. Photobiol. Sci.*, 2022, **21**, 825–833.

[7] a) H. G. Heller and J. R. Langan, *J. Chem. Soc., Perkin Trans. 2*, 1981, 341–343; b) Y. Yokoyama, H. Hayata, H. Ito and Y. Kurita, *Bull. Chem. Soc. Jpn.*, 1990, **63**, 1607–1610; c) A. P. Glaze, H. G. Heller and J. Whittall, *J. Chem. Soc., Perkin Trans. 2*, 1992, 591–594; d) P. Boule and J. F. Pilichowski, *J. Photochem. Photobiol. A*, 1993, **71**, 51–53.

[8] a) A. Böyum, *Scand. J. Clin. Lab. Invest., Suppl.*, 1968, **97**, 77–89; b) A. Bøyum, D. Løvhaug, L. Tresland and E. M. Nordlie, *Scand. J. Immunol.*, 1991, **34**, 697–712.

[9] a) C. A. Schneider, W. S. Rasband and K. W. Eliceiri, *Nat. Methods*, 2012, **9**, 671–675; b) S. Bolte, F. P. Cordelières, *J. Microsc.*, 2006, **224**, 213–232.
